# MRSA Strains in Nepalese Rhesus Macaques (*Macaca mulatta*) and Their Environment

**DOI:** 10.3389/fmicb.2019.02505

**Published:** 2019-11-05

**Authors:** Marilyn C. Roberts, Prabhu Raj Joshi, Stefan Monecke, Ralf Ehricht, Elke Müller, Darius Gawlik, Saroj Paudel, Mahesh Acharya, Sankalpa Bhattarai, Sujana Pokharel, Reshma Tuladhar, Mukesh K. Chalise, Randall C. Kyes

**Affiliations:** ^1^Department of Environmental and Occupational Health, School of Public Health, University of Washington, Seattle, WA, United States; ^2^Nepalese Farming Institute, Kathmandu, Nepal; ^3^Leibniz Institute of Photonic Technology, Jena, Germany; ^4^InfectoGnostics – Research Campus Jena, Jena, Germany; ^5^Institute for Medical Microbiology and Hygiene, Medical Faculty “Carl Gustav Carus”, Technische Universität Dresden, Dresden, Germany; ^6^PTC Phage Technology Center GmbH, Bönen, Germany; ^7^Central Department of Microbiology, Tribhuvan University, Kirtipur, Nepal; ^8^Nepal Biodiversity Research Society, Central Department of Zoology, Tribhuvan University, Kirtipur, Nepal

**Keywords:** Nepal, environment, human, MRSA, *Macaca mulatta*, multidrug resistance

## Abstract

This study looked at 227 saliva samples from Rhesus macaques (*Macaca mulatta*) and 218 samples from the surrounding environments. From these samples, MRSA isolates were collected from Rhesus saliva samples (*n* = 13) and environmental samples (*n* = 19) near temple areas in Kathmandu, Nepal. For comparison, selected MRSA isolates (*n* = 5) were obtained from patients with wound infections from a Kathmandu hospital. All isolates were characterized using Abbott StaphyType^®^ DNA microarrays. Eighteen isolates (62%) from monkeys (*n* = 4; 31%) and environmental samples (*n* = 14; 74%), were CC22-MRSA-IV. Most (*n* = 16) of them carried both, the PVL locus and toxic shock toxin gene (*tst1*), an unusual combination which is the same as in previously characterized strain from Nepalese macaques and pigs. The five human isolates also belonged to that strain type. Eight monkey MRSA isolates were CC361-MRSA-IV. One MRSA from a monkey and one from an environmental sample, were CC88-MRSA-V. Other environmental MRSA included one each, CC121-MRSA-VT, and CC772 -MRSA-V. Two were CC779-MRSA-VT, potentially a novel clone. All MRSA carried the *blaZ* gene. The *aacA–aphD*, *dfrA*, and *erm* (C) genes were very common in isolates from all sources. One macaque MRSA carried the resistance genes *aphA3* and *sat*, neither previously identified in primate MRSA isolates. This current study suggests that humans could be a potential source of the MRSA in the macaques/environment and transmission may be linked to humans feeding the primates and/or living in close proximity to each other.

## Introduction

In Soge et al. (2016), we characterized MRSA isolates from 596 *Macaca* spp. nasals samples in a United States Primate Center. A total of 105 samples was MRSA positive. This included isolates of a novel, previously uncharacterized sequence type (ST3268) and ST188 which is a rare ST in the United States being found primarily from people in South-East Asia (Soge et al., 2016). The genetically highly related ST188 isolates, were isolated from humans working with the animals and from composite primate environmental facility samples. The isolates were characterized by PCR, multilocus sequence typing (MLST) and by whole genome sequencing and SNP analysis. Other isolates from the same Primate Center were characterized by PFGE analysis and by Abbott StaphyType^®^ DNA microarrays (Roberts et al., 2018a). The data suggested an importation of the MRSA strains together with the primates from outside rather than an introduction by local staff members.

It was therefore of interest to look at wild *Macaca* spp. to determine if ether ST188 or ST3268 could be identified in these animals species suggesting a possible source for the outbreaks in the United States Primate Centers.

More recently we have published a study on the characterization of MRSA isolates cultured from 59 wild rhesus macaques (*Macaca mulatta*) saliva samples collected in Roberts et al. (2018b). The animals were living in and around temple areas of the Kathmandu valley in Nepal, where human-macaque interaction is common. In this preliminary study, we found four (6.8%) MRSA with three sequence type (ST) 22 SCC*mec* type IV and one ST239 SCC*mec* type III. ST22-MRSA-IV (EMRSA-15) is a pandemic MRSA isolated both in hospital and community settings around the world although there are also other strains with different SCC*mec* IV subtypes and other toxin genes profiles (Senok et al., 2016). ST22 (clonal complex, CC22) SCC*mec* IV was first identified in Nepal from hospitalized patients in 2012 (Pokhrel et al., 2016) although other studies did not identify CC22 in hospital samples (Joshi et al., 2017). The data from the previous work lead us to hypothesize that humans were a likely source of the ST22-MRSA and ST239-MRSA isolates in the wild Nepalese macaques.

To our best knowledge, five studies from Nepal characterized MRSA in hospital settings obtained from both, patients or healthcare workers (Ansari et al., 2014; Pahadi et al., 2014; Shrestha et al., 2014; Pokhrel et al., 2016; Khatri et al., 2017). Isolation rates varied from 14.6 to 69%. Some identified ST22 and other ST types while in some studies STs were not determined (Shrestha et al., 2014; Joshi et al., 2017).

We proposed that a larger study looking at samples from more primates, their environment, and from hospitalized Nepalese patients during the same year could provide further evidence of shared MRSA isolates in Nepal. In addition, it also could provide information on a possible strain importation into Nepal from other countries. The aim of the current study was to determine if the MRSA isolates with the same CC from the primates, and the environment carried the same virulence genes and specific antibiotic resistance genes and were related to other CC previously characterized (Monecke et al., 2011, 2016, 2018; Roberts et al., 2018b).

In this study, we were able to take 227 samples from *M. mulatta* and 218 samples from the surrounding environments from five areas including the Bajrayogini temple site which is outside the Kathmandu valley. In addition, five randomly selected recent MRSA isolates from wound sites from different patients in one Kathmandu hospital were collected. We used a DNA microarray based assay to differentiate all the MRSA isolates as previously described (Monecke et al., 2011). This was done to compare isolates clonal complex, identify SCC*mec* elements, the presence or absence of difference virulence genes like Panton–Valentine leukocidin (PVL) and carriage of other specific clinically important staphylococcal genes (Monecke et al., 2011). We felt this was the easiest, fastest and most cost effective method for characterization of many different genes since previously we have found that this DNA microarray analysis correlates with PFGE analysis, antimicrobial susceptibility testing, and MLST typing in MRSA isolated from *Macaca* spp. from a United States Primate Center (Roberts et al., 2018a) and from MRSA isolated from swine herds and wild primate in Nepal (Roberts et al., 2018b).

## Materials and Methods

### *Macaca mulatta* Collection

A total of 227 saliva samples from wild rhesus macaques (*M. mulatta*) living in and around temple areas of the Kathmandu valley, where human-macaque interaction is common, were collected on February 2018. This included all animals that took swabs and chewed them. All swabs were collected right after they were discarded with one swab/animal as previously described (Soge et al., 2016). The areas; Bajrayogini, Nilbarahi, Pashupatinath, Swayambhu, and Thapathali were sampled with Bajrayogini being the farthest site from the city of Kathmandu. The collection technique involved an adaptation of the non-invasive oral sampling method described by Evans and Roberts using SalivaBio Children’s Swabs (Salimetrics LLC, State College, PA, United States) (Evans et al., 2015; Roberts et al., 2018b). Swabs were soaked in a sterile glucose solution (10% w/v) and thrown to the macaques. A new pair of disposable gloves was used before taking the swab out of the tube and providing it to the monkey. After chewing for several seconds/minutes, the monkey realized the swab was not food and discarded it. The swab was then collected by a second person using sterile gloves who stored it individually in a new tube that had been labeled. The storage tube contained enrichment broth Bacto^®^ m Staphylococcus Broth (Difco Laboratories, Sparks, MD, United States) that was to prevent fungal growth, supplemented with a final concentration of 75 mg/L of polymyxin B, 0.01% potassium tellurite and either with or without 12.5 mg/L nystatin (Sigma-Aldrich, St. Louis, MO, United States). The tubes were returned to the laboratory the same day and incubated at 37°C until turbid (24–96 h) as previously described (Roberts et al., 2011a). The broth was streaked for isolation onto mannitol salt agar plates (HiMedia Laboratories, Mumbai, India) and yellow colonies were sub-cultured onto blood agar plates (HiMedia Laboratories, Mumbai, India). Colonies that had β-hemolysis were verified as *Staphylococcus aureus* as described below.

### Environmental Samples

Environmental surface samples (*n* = 218) were collected in July 2018 from Bajrayogini, Nilbarahi, Pashupatinath, Swayambhu, and Thapathali. High touch surfaces were selected at the temple sites, which were areas that the primates often touched. Solid surfaces were swabbed with sponges to collect the bacteria on the surfaces as previously described (Michael et al., 2016).

### Human Samples

Five random isolates from unrelated patients with wound infections were obtained for comparison. Beside their methicillin resistance, nothing was previously known about these isolates. Ethical approval of the clinical MRSA isolates was obtained from the Kist Medical College and Teaching Hospital, Imadol, Lalitpur, Nepal (IRC No. 00100/016/017).

### Ethics Statement Primates

The research protocol for the sampling of free-ranging primates in the Kathmandu area was approved by the Kathmandu District Forest Office, Department of Forest under the Ministry of Forestry, Government of Nepal (Reference Letter Number: 074/074/1010). This research also complied with the animal use protocol for primates (#3143-04) approved by the Institutional Animal Care and Use Committee at the University of Washington, United States, and the American Society of Primatologists (ASP) Principles for the Ethical Treatment of Non-human Primates.

### Identification of MRSA From Primates and Environment

Colonies that showed β-hemolysis on blood agar plates were verified as *S. aureus* by Gram stain and with the Staphaurex test as previously described (Thermo Fisher Scientific Remel Products, Lenexa, KS, United States; Roberts et al., 2011b). MRSA isolates were initially identified by their ability to grow on Mueller-Hinton agar (HiMedia Laboratories, India) supplemented with 4 mg/L of oxacillin (HiMedia Laboratories, India). Identification as MRSA was confirmed using the Thermo Scientific PBP2’ latex agglutination test kit according to the manufacturer’s instructions (Thermo Fisher Scientific Remel Products, Lenexa, KS, United States; Roberts et al., 2011b).

### DNA Microarray Analysis

Microarrays were used to screen for a presence or absence of a multitude of genes, including antibiotic resistance markers, virulence factors, species-specific controls and typing markers. This allowed rapid detection of these genes and assigning isolates to clonal complexes and strains. It was not intended to study the expression of these genes.

The Abbott StaphyType^®^ DNA microarray based assay was used for all isolates as previously described (Monecke et al., 2011). This system has previously been used for a variety of studies on MRSA (Monecke et al., 2011, 2016, 2018; Kinnevey et al., 2012). The microarray typing includes 334 target sequences and ∼170 separate genes and allelic variants including species markers, SCC*mec*, capsule, *agr* group typing markers, common antibiotic resistance genes, toxins and microbial surface components recognizing adhesive matrix molecules (MSCRAMM) genes. The latter genes comprise among others *clfA* and *clfB* (encoding clumping factors A and B), *fnbA* and *fnbB* (encoding fibronectin binding proteins A and B), *fib* (encoding fibrinogen binding protein), *eno* (encoding laminin binding protein), and *cna* (encoding collagen binding protein), the gene products of which play a role in the initial attachment of bacteria to host tissue. The detailed protocol as well as the sequences of primers and probes have previously been published (Monecke et al., 2011).

Isolates were assigned to clonal complexes (CCs) by automated comparison of the microarray hybridization profiles to a large database of previously characterized isolates (Monecke et al., 2011). Representative isolates were characterized using a second microarray that facilitates subtyping of SCC*mec* elements (Monecke et al., 2016).

## Results

### Isolation of MRSA

From the 227 primate saliva samples, 13 (5.7%) were MRSA positive. Multiple positive primates were cultured in four of the five areas sampled: Bajrayogini (*n* = 4), Pashupatinath (*n* = 3), Swayambhu (*n* = 2), and Thapathali (*n* = 4). While 19 (8.7%) out of the 218 environmental samples were MRSA positive. They originated from all five areas sampled; Bajrayogini (*n* = 3), Nilbarahi (*n* = 2), Pashupati (*n* = 6), Swayambhu (*n* = 5), and Thapathali (*n* = 3). Five human MRSA isolates were included and all 37 MRSA isolates were further characterized.

### CC and SCC*mec* Typing

Typing data, geographic origins and sample sources/host species for all the MRSA in the current study are listed in Table 1 and in the Supplementary File. CC22 included monkey samples (*n* = 4; 31%) from Bajrayogini and Pashupatinath areas, environmental samples (*n* = 14; 74%) from Nibarahi, Pashupatinath, Swayambhu, and Thapathali areas, and human (*n* = 5) samples from Kathmandu hospital (Table 1). Twenty-three (64%) of the 37 MRSA were assigned to CC22. Twenty-one of them were essentially identical in carrying PVL and *tst1* genes. Representative isolates of that cluster yielded an SCC*mec* type IVa as previously found in MRSA from Nepalese macaques and swine, isolated in 2017 (Roberts et al., 2018b; see section “Discussion”). One isolate with *tst1* but without PVL also had the same SCC*mec* subtype; while one isolate with PVL alone was assigned to SCC*mec* type IVc.

**TABLE 1:** MRSA strains, sample types, and geographic background.

**Clonal Complex**	**Strain**	**Host/Sample type**	**Isolate number**	**Geographic origin within Nepal**
CC22	CC22-MRSA-IV (PVL+/tst1 +)	Rhesus	640, 657, 801,804	Pashupatinath (*n* = 2) Bajrayogini (*n* = 2)
		Environmental	1005,1027,2021, 2027, 2051, 3009, 3022, 4021, 4033, 5018, 5040, 5028, 5025-2	Thapathali (*n* = 2) Pashupatinath (*n* = 3) Nilbarahi (*n* = 2) Bajrayogini (*n* = 2) Swayambhu (*n* = 3)
		Human	21, 22, 23, 24, 25	Kathmandu (*n* = 5)
	CC22-MRSA-IV (*tst1*+)	Environmental	4007	Bajrayogini (*n* = 1)
	CC22-MRSA-IV (PVL+)	Environmental	5008	Swayambhu (*n* = 1)
CC88	CC88-MRSA-V	Rhesus	556	Swayambhu (*n* = 1)
	CC88-MRSA-V (PVL+)	Environmental	1007	Thapathali (*n* = 1)
CC121	CC121-MRSA-VT	Environmental	2039	Pashupatinath (*n* = 1)
CC361	CC361-MRSA-IV	Rhesus	106, 115, 117, 120, 505, 611, 807, 811	Thapathali (*n* = 4) Pashupatinath (*n* = 1) Bajrayogini (*n* = 2) Swayambhu (*n* = 1)
CC772	CC772-MRSA-V (PVL +), “Bengal Bay Clone”	Environmental	2043	Pashupatinath (*n* = 1)
CC779	CC779-MRSA-VT	Environmental	2006, 5025-1	Pashupatinath (*n* = 1) Swayambhu (*n* = 1)

Eight monkey MRSA isolates from Bajrayogini, Pashupatinath and Thapathali areas were CC361, SCC*mec* type IVa. One MRSA isolated from a monkey (Swayambhu) and another one from an environmental sample (Thapathali) were both CC88-MRSA-V but differed in PVL status (Table 1). The remaining environmental MRSA included one each, CC121-MRSA-VT (Pashupatinath), and CC772-MRSA-V (Pashupatinath), and two CC779 SCC*mec* type VT (Pashupatinath and Swayambhu areas) (Table 1).

### Antibiotic Resistance Genes

Details on resistance gene carriage and on SCC*mec* subtypes are shown in Table 2 and in the Supplementary File that includes the full hybridization patterns of all isolates. All 23 CC22 isolates carried the aminoglycoside resistance gene *aacA–aadD* as well as the trimethoprim resistance gene *dfrA*. Seventeen isolates carried the *erm* (C) gene (encoding macrolide/lincosamide resistance), while two MRSA from macaques, three MRSA from environmental samples and one human MRSA lacked it. The one macaque and environmental CC88 MRSA isolates both carried the *aacA–aphD* gene but only the macaque MRSA isolate carried the *aphA3* and *sat* resistance genes, neither previously identified in primate MRSA (Table 2).

**TABLE 2:** MRSA strains, SCC*mec* types, and resistance genes^a^.

**Strain**	**Host**	***N***	***mecA***	**SCC*mec* subtype^b^ (number subtyped)**	***blaZ***	***Erm* (C)**	***Msr* (A)**	***Mph* (B)**	***aacA-aphD***	***aadD***	***aphA3***	***sat***	***dfrA***
CC22-MRSA-IV (PVL+/*tst1*+)	Rhesus	4	4	IVa as in MW2 (1)	4	2	–	–	4	–	–	–	4
CC22-MRSA-IV (PVL+/*tst1*+)	Environment	12	12	IVa as in MW2 (1)	12	9	–	–	12	–	–	–	12
CC22-MRSA-IV (PVL+/*tst1*+)	Human	5	5	IVa as in MW2 (2)	5	4	–	–	5	–	–	–	5
CC22-MRSA-IV (*tst1* +)	Environment	1	1	IVa as in MW2 (1)	1	–	–	–	1	–	–	–	1
CC22-MRSA-IV (PVL)	Environment	1	1	IVc as in IS-105 (1)	1	–	–	–	1	1	–	–	1
CC88-MRSA-V	Rhesus	1	1	V as in Bengal Bay (1)	1	1	1	1	1	–	1	1	–
CC88-MRSA-V (PVL +)	Environment	1	1	V as in Bengal Bay (1)	1	1	–	–	1	–	–	–	–
CC121-MRSA-VT	Environment	1	1	VT as in GR1 (1)	1	1	–	–	1	–	–	–	–
CC361-MRSA-IV	Rhesus	8	8	IVa as in MW2 (1)	8	–	–	–	–	–	–	–	–
CC772-MRSA-V (PVL +)	Environment	1	1	V as in Bengal Bay (1)	1	–	1	1	1	–	1	1	–
CC779-MRSA-VT	Environment	2	2	VT as in GR1 (1)	2	2	–	–	2	–	–	–	–

*^*a*^The table shows only genes which were found at least once in this study. Genes which were *not* present in any of the study strains are: *mecC*, SCC*mec* XI, *erm* (A), *erm* (B), *lnu* (A), *mef* (A), *vat* (A), *vat* (B), *vga* (A), *vga* (A), *vgb* (A), *far1, fusC, mupR, tet* (K), *tet* (M), *cat, cfr, fexA, qacA, qacC, vanA, vanB*, and *vanZ*. ^*b*^Subtypes are referred to by designations of reference strains that yield identical SCC patterns on the arrays used. GenBank references for these reference strains are as follows: MW2, BA000033.2; IS-105, AHLR; Bengal Bay (CMFT1723), HF569096.1; GR1, AJLX.*

### Accessory and Virulence Factors

Among the 23 CC22 MRSA, 21 (91%) carried the PVL locus and the *tst1* gene encoding the toxic shock syndrome toxin. These included all monkey (*n* = 4) and human isolates and 12 (86%) out of 14 environmental isolates. For other virulence factors, see Table 3 and the Supplementary File.

**TABLE 3:** MRSA strains and virulence factors^a^.

**Strain**	**Host**	**N**	***tst1***	***sea***	***sea-N315***	***seb***	***sec* + *sel***	***egc***	***sek* + *seq***	**ORF CM 14**	**PVL**	***lukD* + *lukE***	***sak***	***chp***	***scn***	***etA***	***etD***	***edinB***	***cna***	***sasG***
CC22-MRSA-IV	Rhes	4	4	–	–	–	4	4	–	–	4	–	4	4	4	–	–	–	4	4
(PVL + /*tst1* +)	Env	12	12	–	–	–	12	12	–	–	12	–	10	10	10	–	–	–	12	12
	Hum	5	5	–	–	–	5	5	–	–	5	–	5	4	4	–	–	–	5	5
CC22-MRSA-IV (*tst1* +)	Env	1	1	–	–	–	1	1	–	–	–	–	–	–	–	–	–	–	1	1
CC22-MRSA-IV (PVL)	Env	1	–	–	–	–	–	1	–	–	1	–	1	1	1	–	–	–	1	1
CC88-MRSA-V	Rhes	1	–	–	1	–	–	–	1	–	–	1	1	1	1	–	–	–	–	1
CC88-MRSA-V (PVL +)	Env	1	–	1	1	–	–	–	1	–	1	1	1	1	1	–	–	–	–	1
CC121-MRSA-VT	Env	1	–	–	–	–	–	1	–	1	–	1	1	–	1	1	–	–	–	–
CC361-MRSA-IV	Rhes	8	1	–	–	8	–	8	8	–	–	8	8	–	8	2	–	–	–	8
CC772-MRSA-V (PVL +)	Env	1	–	1	–	–	1	1	–	1	1	–	–	–	1	–	–	–	1	1
CC779-MRSA-VT	Env	2	–	–	–	–	–	–	2	–	–	2	2	2	2	–	2	2	–	2

*^*a*^The table shows only genes which were found at least once in this study. Genes which were *not* present in any of the study strains include: *sea-*320E, *sed, see, seh, sej, ser, lukM* + *lukF*-P83, *etB, edinA, ed.**

## Discussion

### MRSA Isolates

In the current study, 23 (62%) MRSA isolates were CC22 SCC*mec* type IVa and included MRSA from monkey (*n* = 4; 31%), environmental (*n* = 14; 74%), and humans (*n* = 5 out of 5 isolates) samples. These were CC22 isolates previously found in Nepalese macaque (Roberts et al., 2018b). All four of the rhesus isolates were also PVL and *tst1* positive. While one environmental isolate (4007) was also *tst1* positive. The MRSA isolated from environmental samples were more diverse clonally and included CC22 which was most common identified clone but also CC88, CC121, CC772, and CC779 which is very rare (Lim et al., 2012). Of the five different clones, two (C22 and C88) were identified in both monkey and environmental samples.

### CC22-MRSA-IVa

The CC22 SCC*mec* type IV identified in the current study are widespread and common. Though PVL and *tst1* positive isolates are less common but are found in various geographic locations. For example, a study of toxin carriage and SCC*mec* subtypes revealed that there are several distinct strains (Senok et al., 2016) with different geographic distributions and possibly different clinical significance. This includes CC22-MRSA-IVh/j without additional toxin genes beside the *egc* locus, or with enterotoxin genes C and L. This strain is known as UK15 or, in Germany, as “Barnim Epidemic Strain” and it accounts for a majority of MRSA cases in several Western European countries. There is a SCC*mec* IVa strain that carries the toxic shock syndrome toxin gene *tst1*, “Gaza Epidemic Strain,” observed in the Mediterranean, in the Middle East and among people with travel histories pointing into that direction. There are PVL positive strains with SCC*mec* IVc elements (common in the Middle East) or other subtypes (sporadic in Western Europe). Finally there is a multitude of strains with SCC*mec* IV derived composite elements that also include *fusC* or ACME (Scicluna et al., 2010; Shore et al., 2011).

It was surprising to detect in the present study a high number of CC22-MRSA-IVa with PVL and *tst1*. Strains harboring both toxins together are generally rare (Novick, 2003). A reason might be that the *tst1* gene down regulates the expression of other toxins in *S. aureus* (Vojtov et al., 2002; Novick, 2003) and this effect was also observed for PVL (Stieber et al., 2014). This strain was previously observed in swine and in Rhesus from Nepal and its genome was sequenced (Roberts et al., 2018b; NCBI Biosample accession numbers SAMN08146085, SAMN08146086, SAMN08146087, SAMN08146088, SAMN08146089, SAMN08146091, SAMN08146092, SAMN08146093). A strain that appeared identical applying the same array-based methods as used in the present paper was recently observed to emerge in Kuwait (Boswihi et al., 2018). Similar or identical strains also have been reported from Iran (Goudarzi et al., 2017). Given the high number of Nepalese nationals (in 2011, more than 720,000) working in Arabian Gulf countries (Labor Report, 2019) a transmission of a MRSA strain epidemic at the Gulf to Nepal appears likely. We previously observed MRSA strains that occur in both, the Gulf States and Pakistan where a similarly high number of expat Nepalese workers travel to the Gulf region (Jamil et al., 2018).

One isolate [4007] was a CC22-MRSA-IVa positive for *tst1*, but without PVL. This might have been a deletion variant of the strain discussed above that lost the PVL prophage. Another isolate [5008] was assigned to CC22-MRSA-IVc (PVL+). This is another strain for which a Middle Eastern/Arabian Gulf connection can be assumed (Senok et al., 2016).

### CC88-MRSA-V

CC88-MRSA-V was observed twice [556, 1007] with one isolate being PVL-positive. While CC88-MSSA and CC88-MRSA-IV are common and geographically widespread, strains with SCC*mec* V elements are rare. A PVL-positive strain has previously been noted as community-associated MRSA in Western Australia (“WA MRSA-117”). Both isolates observed herein yielded the same SCC*mec* pattern as the CC772-MRSA-V “Bengal Bay Clone” that is common and widespread on the Indian subcontinent (see below) (Pokhrel et al., 2016). This might indicate a local transfer of SCC*mec* and emergence of these CC88 strains which are also found in Nepalese swine MRSA (Roberts et al., 2018b).

### Other Clonal Complexes

One isolate [2039] was identified as CC121-MRSA-VT. CC121 is a clonal complex that can be found in humans essentially worldwide. MRSA from this lineage, however, are very rare and only few isolates from different geographic regions have yet been described.

Another eight isolates [106, 115, 117,120, 505, 611, 807, 811] were assigned to CC361-MRSA-IVa. This is less well known strain, characterized by the presence of the *egc* enterotoxin gene cluster, as well as, of enterotoxin genes *seb, sek, seq*, and/or *tst1*. Related/identical isolates were found, among humans, in the United Arab Emirates (unpublished isolate, courtesy to Abiola Senok) and in Western Australia (“WA MRSA-29,” courtesy to Geoffrey Coombs).

One environmental isolate [2043] belonged to CC772-MRSA-V and was PVL positive. It thus represented the “Bengal Bay Clone” which is a multi-resistant, mainly community-associated MRSA strain that recently spread on the Indian subcontinent as well as in the Middle East and among travelers to/from these regions.

Two environmental isolates [2006, 5025-1] were CC779-MRSA-VT [2006, 5025]. We did not observe that strain before; and thus we cannot draw conclusions on its provenance. CC779 in general appears to be rare with few descriptions of other CC779-MRSA strains originating from Australia (“WA MRSA-100”) and Ireland (Kinnevey et al., 2012).

## Conclusion

In general, the MRSA strains in the current study indicate epidemiological links, using CC and SCC*mec* genes, to other countries of the Indian subcontinent and to the Middle East. Strains that have been detected in monkeys also have been found, either in this or in other studies, in humans (Soge et al., 2016; Roberts et al., 2018a, b). For the (environmental) CC779-MRSA-VT, no conclusions can be drawn due to a lack of data. However, this lineage has been found in humans before (see above) and we are not aware of any published observations on its presence in animals. In conclusion, it can be speculated that the detection of MRSA in Nepalese Rhesus can be attributed at least in a majority of cases to contamination/infection during contacts to humans or to human offal. Thus, humans can not only be infected with zoonotic pathogens by too close contacts to wild animals; they may also transmit human pathogens into wildlife posing a possible hazard to wild animals whose population are already endangered and under stress. At least, the impact on the monkeys might be limited in this particular case as a related species of macaques seemed to be rather resistant toward PVL (Loffler et al., 2010).

A current limitation to the study is that, although array analyses might give a clue on the origin of an epidemic strain, the data does not allow phylogenetic analyses to the same extend as full genome sequencing. Thus hypothesis on a chain of transmission of the PVL+/*tst*1+ positive CC22-MRSA-IVa strain from the Gulf to Nepal and from humans to monkey is plausible but not proven. Proving that MRSA isolates in primates come from the Gulf to Nepal would require a large sequencing study with a very different scope involving strains from many countries beyond Nepal.

Future studies should expand the knowledge on MRSA in humans in Nepal and on their possible impact on domestic and wild animals. It would also be interesting to know if *S. aureus* occurs in Rhesus monkeys that do not have contact to humans and, if that was the case, to which *S. aureus* lineage they belong.

## Data Availability Statement

All datasets generated for this study are included in the article/Supplementary Material.

## Ethics Statement

Ethical approval was obtained from the Nepalese Hospital for the clinical MRSA isolates (IRC No. 00100/016/017). The patients/participants provided their written informed consent to participate in this study. The research protocol for the sampling of free-ranging primates in the Kathmandu area was approved by the Kathmandu District Forest Office, Department of Forest under the Ministry of Forestry, Government of Nepal (Reference Letter Number: 074/074/1010). This research also complied with the animal use protocol for primates (#3143-04) approved by the Institutional Animal Care and Use Committee at the University of Washington, United States, and the American Society of Primatologists (ASP) Principles for the Ethical Treatment of Non-human Primates. Written informed consent was obtained from the individual(s) for the publication of any potentially identifiable images or data included in this manuscript.

## Author Contributions

MR and RK coordinated the study and supervised the original field and data collection. PJ coordinated in country collection of the MRSA samples. SaP, MA, and SB helped to collect the field samples of animals and environment. SuP and RT provided Nepali human samples. SM, RE, EM, and DG did all the analysis as well as designing and helped with writing the manuscript along with MC. MC helped with facilitating access to isolates.

## Conflict of Interest

The authors declare that the research was conducted in the absence of any commercial or financial relationships that could be construed as a potential conflict of interest.
