# Supplementary material for: MRSA Strains in Nepalese Rhesus Macaques (Macaca mulatta) and Their Environment
Source: Front Microbiol. 2019 Nov 5;10:2505. doi: 10.3389/fmicb.2019.02505 (PMC6849405; doi:10.3389/fmicb.2019.02505)
Supplement: Supplementary file 1 [file Table_1.pdf]

ISOLATE	TYPING DATA		Geographic origin	Host	SCC subtype
	CLONAL COMPLEX	STRAIN AFFILIATION			
**Washington-08_sample-640**	>CC22	CC22-MRSA-IV (PVL+/tst+)	Nepal,Pashupatinath	Monkey	SCCmec IVa (MW2)
**Washington-09_sample-657**	>CC22	CC22-MRSA-IV (PVL+/tst+)	Nepal,Pashupatinath	Monkey	
**Washington-10_sample-801**	>CC22	CC22-MRSA-IV (PVL+/tst+)	Nepal,Bajrayogini	Monkey	
**Washington-11_sample-804**	>CC22	CC22-MRSA-IV (PVL+/tst+)	Nepal,Bajrayogini	Monkey	
**Washington-14_sample-1005**	>CC22	CC22-MRSA-IV (PVL+/tst+)	Nepal, Thapathali	Environmental	SCCmec IVa (MW2)
**Washington-16_sample-1027**	>CC22	CC22-MRSA-IV (PVL+/tst+)	Nepal, Thapathali	Environmental	
**Washington-18_sample-2021**	>CC22	CC22-MRSA-IV (PVL+/tst+)	Nepal, Pashupati	Environmental	
**Washington-19_sample-2027**	>CC22	CC22-MRSA-IV (PVL+/tst+)	Nepal, Pashupati	Environmental	
**Washington-22_sample-2051**	>CC22	CC22-MRSA-IV (PVL+/tst+)	Nepal, Pashupati	Environmental	
**Washington-23_sample-3009**	>CC22	CC22-MRSA-IV (PVL+/tst+)	Nepal, Nilbarahi	Environmental	
**Washington-24_sample-3022**	>CC22	CC22-MRSA-IV (PVL+/tst+)	Nepal, Nilbarahi	Environmental	
**Washington-26_sample-4021**	>CC22	CC22-MRSA-IV (PVL+/tst+)	Nepal, Bajrayogini	Environmental	
**Washington-27_sample-4033**	>CC22	CC22-MRSA-IV (PVL+/tst+)	Nepal, Bajrayogini	Environmental	
**Washington-29_sample-5018**	>CC22	CC22-MRSA-IV (PVL+/tst+)	Nepal, Swyambhu	Environmental	
**Washington-31_sample-5040**	>CC22	CC22-MRSA-IV (PVL+/tst+)	Nepal, Swyambhu	Environmental	
**Washington-30_sample 5025_Subclone 2**	>CC22	CC22-MRSA-IV (PVL+/tst+)	Nepal, Swyambhu	Environmental	
**Washington-32_sample-21**	>CC22	CC22-MRSA-IV (PVL+/tst+)	Nepal, Kathmandu	Human	
**Washington-33_sample-22**	>CC22	CC22-MRSA-IV (PVL+/tst+)	Nepal, Kathmandu	Human	
**Washington-34_sample-23**	>CC22	CC22-MRSA-IV (PVL+/tst+)	Nepal, Kathmandu	Human	
**Washington-35_sample-24**	>CC22	CC22-MRSA-IV (PVL+/tst+)	Nepal, Kathmandu	Human	SCCmec IVa (MW2)
**Washington-36_sample-25**	>CC22	CC22-MRSA-IV (PVL+/tst+)	Nepal, Kathmandu	Human	SCCmec IVa (MW2)
**Washington-25_sample-4007**	>CC22	CC22-MRSA-IV (tst1+)	Nepal, Bajrayogini	Environmental	SCCmec IVa (MW2)
**Washington-28_sample-5008**	>CC22	CC22-MRSA-IVc (PVL+)	Nepal, Swyambhu	Environmental	SCCmec IVc (IS-105)
**Washington-06_sample-556**	>CC88	CC88-MRSA-V	Nepal,Swyambhu	Monkey	SCCmec V (Bengal Bay)
**Washington-15_sample-1007**	>CC88	CC88-MRSA-V (PVL+), WA MRSA-117	Nepal, Thapathali	Environmental	SCCmec V (Bengal Bay)
**Washington-20_sample-2039**	>CC121	CC121-MRSA-V/VT	Nepal, Pashupati	Environmental	SCCmec VT (GR1)

ISOLATE	TYPING DATA		Geographic origin	Host	SCC subtype
	CLONAL COMPLEX	STRAIN AFFILIATION			
**Washington-01_sample-106**	**>CC361**	**CC361-MRSA-IV, WA MRSA-29**	Nepal,Thapathali	Monkey	**SCCmec IVa (MW2)**
**Washington-02_sample-115**	**>CC361**	**CC361-MRSA-IV, WA MRSA-29**	Nepal,Thapathali	Monkey	
**Washington-03_sample-117**	**>CC361**	**CC361-MRSA-IV, WA MRSA-29**	Nepal,Thapathali	Monkey	
**Washington-04_sample-120**	**>CC361**	**CC361-MRSA-IV, WA MRSA-29**	Nepal,Thapathali	Monkey	
**Washington-05_sample-505**	**>CC361**	**CC361-MRSA-IV, WA MRSA-29**	Nepal,Swyambhu	Monkey	
**Washington-07_sample-611**	**>CC361**	**CC361-MRSA-IV, WA MRSA-29**	Nepal,Pashupatinath	Monkey	
**Washington-12_sample-807**	**>CC361**	**CC361-MRSA-IV, WA MRSA-29**	Nepal,Bajrayogini	Monkey	
**Washington-13_sample-811**	**>CC361**	**CC361-MRSA-IV, WA MRSA-29**	Nepal,Bajrayogini	Monkey	
**Washington-21_sample-2043**	**>CC772**	**CC772-MRSA-V (PVL+), "Bengal Bay Clone"**	Nepal, Pashupati	Environmental	**SCCmec V (Bengal Bay)**
**Washington-17_sample-2006**	**>CC779**	**CC779-MRSA-V/VT**	Nepal, Pashupati	Environmental	**SCCmec VT (GR1)**
**Washington-30_sample-5025_Subclone 1**	**>CC779**	**CC779-MRSA-V/VT**	Nepal, Swyambhu	Environmental	

From the previous study - for comparison:

**Washington-37_sample-48_ST22_Primate**	**>CC22**	**CC22-MRSA-IV (PVL+/tst+)**	Nepal	Rhesus (Macaca mu	**SCCmec IVa (MW2)**
**Washington-38_sample-06_ST22_Swine**	**>CC22**	**CC22-MRSA-IV (PVL+/tst+)**	Nepal	Swine	**SCCmec IVa (MW2)**

ISOLATE	SPECIES MARKER							
	Domain 1 of 23S-rRNA	glyceraldehyde 3-phosphate dehydrogenase, locus 1	katalase A	coagulase	thermostable extracellular nuclease	staphylococcal protein A	IgG-binding protein	staphylococcal accessory regulator A
	**Ribos. STAU**	***gapA***	***katA***	**CoA**	***nuc1***	***spa***	***sbi***	***sarA***
	median(s_aur_rrn_1_pm)	median(gapA_11)	median(katA_11)	median(probe_coa_	median(hp_nuc1_611)	median(proteinA_1	median(sbi-var1_1	median(hp_sarA_611)
**Washington-08_sample-640**	**POS**	**POS**	**POS**	**POS**	**POS**	**POS**	**POS**	**POS**
**Washington-09_sample-657**	**POS**	**POS**	**POS**	**POS**	**POS**	**POS**	**POS**	**POS**
**Washington-10_sample-801**	**POS**	**POS**	**POS**	**POS**	**POS**	**POS**	**POS**	**POS**
**Washington-11_sample-804**	**POS**	**POS**	**POS**	**POS**	**POS**	**POS**	**POS**	**POS**
**Washington-14_sample-1005**	**POS**	**POS**	**POS**	**POS**	**POS**	**POS**	**POS**	**POS**
**Washington-16_sample-1027**	**POS**	**POS**	**POS**	**POS**	**POS**	**POS**	**POS**	**POS**
**Washington-18_sample-2021**	**POS**	**POS**	**POS**	**POS**	**POS**	**POS**	**POS**	**POS**
**Washington-19_sample-2027**	**POS**	**POS**	**POS**	**POS**	**POS**	**POS**	**POS**	**POS**
**Washington-22_sample-2051**	**POS**	**POS**	**POS**	**POS**	**POS**	**POS**	**POS**	**POS**
**Washington-23_sample-3009**	**POS**	**POS**	**POS**	**POS**	**POS**	**POS**	**POS**	**POS**
**Washington-24_sample-3022**	**POS**	**POS**	**POS**	**POS**	**POS**	**POS**	**POS**	**POS**
**Washington-26_sample-4021**	**POS**	**POS**	**POS**	**POS**	**POS**	**POS**	**POS**	**POS**
**Washington-27_sample-4033**	**POS**	**POS**	**POS**	**POS**	**POS**	**POS**	**POS**	**POS**
**Washington-29_sample-5018**	**POS**	**POS**	**POS**	**POS**	**POS**	**POS**	**POS**	**POS**
**Washington-31_sample-5040**	**POS**	**POS**	**POS**	**POS**	**POS**	**POS**	**POS**	**POS**
**Washington-30_sample 5025_Subclone 2**	**POS**	**POS**	**POS**	**POS**	**POS**	**POS**	**POS**	**POS**
**Washington-32_sample-21**	**POS**	**POS**	**POS**	**POS**	**POS**	**POS**	**POS**	**POS**
**Washington-33_sample-22**	**POS**	**POS**	**POS**	**POS**	**POS**	**POS**	**POS**	**POS**
**Washington-34_sample-23**	**POS**	**POS**	**POS**	**POS**	**POS**	**POS**	**POS**	**POS**
**Washington-35_sample-24**	**POS**	**POS**	**POS**	**AMB**	**POS**	**POS**	**POS**	**POS**
**Washington-36_sample-25**	**POS**	**POS**	**POS**	**AMB**	**POS**	**POS**	**POS**	**POS**
**Washington-25_sample-4007**	**POS**	**POS**	**POS**	**POS**	**POS**	**POS**	**POS**	**POS**
**Washington-28_sample-5008**	**POS**	**POS**	**POS**	**POS**	**POS**	**POS**	**POS**	**POS**
**Washington-06_sample-556**	**POS**	**POS**	**POS**	**POS**	**POS**	**POS**	**POS**	**POS**
**Washington-15_sample-1007**	**POS**	**POS**	**POS**	**POS**	**POS**	**POS**	**POS**	**POS**
**Washington-20_sample-2039**	**POS**	**POS**	**POS**	**POS**	**POS**	**POS**	**POS**	**POS**

ISOLATE	SPECIES MARKER							
	Domain 1 of 23S-rRNA	glyceraldehyde 3-phosphate dehydrogenase, locus 1	katalase A	coagulase	thermostable extracellular nuclease	staphylococcal protein A	IgG-binding protein	staphylococcal accessory regulator A
	**Ribos. STAU**	***gapA***	***katA***	**CoA**	***nuc1***	***spa***	***sbi***	***sarA***
	median(s_aur_rrn_1_pm)	median(gapA_11)	median(katA_11)	median(probe_coa_	median(hp_nuc1_611)	median(proteinA_1	median(sbi-var1_1:	median(hp_sarA_611)
**Washington-01_sample-106**	**POS**	**POS**	**POS**	**POS**	**POS**	**POS**	**POS**	**POS**
**Washington-02_sample-115**	**POS**	**POS**	**POS**	**POS**	**POS**	**POS**	**POS**	**POS**
**Washington-03_sample-117**	**POS**	**POS**	**POS**	**POS**	**POS**	**POS**	**POS**	**POS**
**Washington-04_sample-120**	**POS**	**POS**	**POS**	**POS**	**POS**	**POS**	**POS**	**POS**
**Washington-05_sample-505**	**POS**	**POS**	**POS**	**POS**	**POS**	**POS**	**POS**	**POS**
**Washington-07_sample-611**	**POS**	**POS**	**POS**	**POS**	**POS**	**POS**	**POS**	**POS**
**Washington-12_sample-807**	**POS**	**POS**	**POS**	**POS**	**POS**	**POS**	**POS**	**POS**
**Washington-13_sample-811**	**POS**	**POS**	**POS**	**POS**	**POS**	**POS**	**POS**	**POS**
**Washington-21_sample-2043**	**POS**	**POS**	**POS**	**POS**	**POS**	**POS**	**POS**	**POS**
**Washington-17_sample-2006**	**POS**	**POS**	**POS**	**POS**	**POS**	**POS**	**POS**	**POS**
**Washington-30_sample-5025_Subclone 1**	**POS**	**POS**	**POS**	**POS**	**POS**	**POS**	**POS**	**POS**

**From the previous study - for comparison:**								
**Washington-37_sample-48_ST22_Primate**	**POS**	**POS**	**POS**	**POS**	**POS**	**POS**	**POS**	**POS**
**Washington-38_sample-06_ST22_Swine**	**POS**	**POS**	**POS**	**POS**	**POS**	**POS**	**POS**	**POS**

ISOLATE	REGULATORY GENES									
	histidine protein kinase, sae locus	sensor protein	accessory gene regulator allele I				accessory gene regulator allele II			
	saeS	***vraS***	**agrI (total)**	**agrB-I**	**agrC-I**	**agrD-I**	**agrII (total)**	**agrB-II**	**agrC-II**	**agrD-II**
	median(hp_saeS_611)	median(hp_vraS_6	median(agrB-I_11;	median(agrB-I_11)	median(agrC-I_12)	median(agrD-I_11;	median(agrB-II_11	median(agrB-II_11	median(agrC-II_11	median(agrD-II_11
**Washington-08_sample-640**	**POS**	**POS**	**POS**	**POS**	**NEG**	**POS**	**NEG**	**NEG**	**NEG**	**NEG**
**Washington-09_sample-657**	**POS**	**POS**	**POS**	**POS**	**NEG**	**POS**	**NEG**	**NEG**	**NEG**	**NEG**
**Washington-10_sample-801**	**POS**	**POS**	**POS**	**POS**	**NEG**	**POS**	**NEG**	**NEG**	**NEG**	**NEG**
**Washington-11_sample-804**	**POS**	**POS**	**POS**	**POS**	**NEG**	**POS**	**NEG**	**NEG**	**NEG**	**NEG**
**Washington-14_sample-1005**	**POS**	**POS**	**POS**	**POS**	**NEG**	**POS**	**NEG**	**NEG**	**NEG**	**NEG**
**Washington-16_sample-1027**	**POS**	**POS**	**POS**	**POS**	**AMB**	**POS**	**NEG**	**NEG**	**NEG**	**NEG**
**Washington-18_sample-2021**	**POS**	**POS**	**POS**	**POS**	**NEG**	**POS**	**NEG**	**NEG**	**NEG**	**NEG**
**Washington-19_sample-2027**	**POS**	**POS**	**POS**	**POS**	**NEG**	**POS**	**NEG**	**NEG**	**NEG**	**NEG**
**Washington-22_sample-2051**	**POS**	**POS**	**POS**	**POS**	**POS**	**POS**	**NEG**	**NEG**	**NEG**	**NEG**
**Washington-23_sample-3009**	**POS**	**POS**	**POS**	**POS**	**AMB**	**POS**	**NEG**	**NEG**	**NEG**	**NEG**
**Washington-24_sample-3022**	**POS**	**POS**	**POS**	**POS**	**NEG**	**POS**	**NEG**	**NEG**	**NEG**	**NEG**
**Washington-26_sample-4021**	**POS**	**POS**	**POS**	**POS**	**NEG**	**POS**	**NEG**	**NEG**	**NEG**	**NEG**
**Washington-27_sample-4033**	**POS**	**POS**	**POS**	**POS**	**AMB**	**POS**	**NEG**	**NEG**	**NEG**	**NEG**
**Washington-29_sample-5018**	**POS**	**POS**	**POS**	**POS**	**NEG**	**POS**	**NEG**	**NEG**	**NEG**	**NEG**
**Washington-31_sample-5040**	**POS**	**POS**	**POS**	**POS**	**AMB**	**POS**	**NEG**	**NEG**	**NEG**	**NEG**
**Washington-30_sample 5025_Subclone 2**	**POS**	**POS**	**POS**	**POS**	**POS**	**POS**	**NEG**	**NEG**	**NEG**	**NEG**
**Washington-32_sample-21**	**POS**	**POS**	**POS**	**POS**	**NEG**	**POS**	**NEG**	**NEG**	**NEG**	**NEG**
**Washington-33_sample-22**	**POS**	**POS**	**POS**	**POS**	**NEG**	**POS**	**NEG**	**NEG**	**NEG**	**NEG**
**Washington-34_sample-23**	**POS**	**POS**	**POS**	**POS**	**NEG**	**POS**	**NEG**	**NEG**	**NEG**	**NEG**
**Washington-35_sample-24**	**POS**	**POS**	**POS**	**POS**	**NEG**	**POS**	**NEG**	**NEG**	**NEG**	**NEG**
**Washington-36_sample-25**	**POS**	**POS**	**POS**	**POS**	**NEG**	**POS**	**NEG**	**NEG**	**NEG**	**NEG**
**Washington-25_sample-4007**	**POS**	**POS**	**POS**	**POS**	**NEG**	**POS**	**NEG**	**NEG**	**NEG**	**NEG**
**Washington-28_sample-5008**	**POS**	**POS**	**POS**	**POS**	**NEG**	**POS**	**NEG**	**NEG**	**NEG**	**NEG**
**Washington-06_sample-556**	**POS**	**POS**	**NEG**	**NEG**	**NEG**	**NEG**	**NEG**	**NEG**	**NEG**	**NEG**
**Washington-15_sample-1007**	**POS**	**POS**	**NEG**	**NEG**	**NEG**	**NEG**	**NEG**	**NEG**	**NEG**	**NEG**
**Washington-20_sample-2039**	**POS**	**POS**	**NEG**	**NEG**	**AMB**	**NEG**	**NEG**	**NEG**	**NEG**	**NEG**

ISOLATE	REGULATORY GENES									
	histidine protein kinase, sae locus	sensor protein	accessory gene regulator allele I				accessory gene regulator allele II			
	saeS	***vraS***	**agrI (total)**	**agrB-I**	**agrC-I**	**agrD-I**	**agrII (total)**	**agrB-II**	**agrC-II**	**agrD-II**
	median(hp_saeS_611)	median(hp_vraS_6	median(agrB-I_11;	median(agrB-I_11)	median(agrC-I_12)	median(agrD-I_11;	median(agrB-II_11	median(agrB-II_11	median(agrC-II_11	median(agrD-II_11
**Washington-01_sample-106**	**POS**	**POS**	**POS**	**POS**	**POS**	**POS**	**NEG**	**NEG**	**NEG**	**NEG**
**Washington-02_sample-115**	**POS**	**POS**	**POS**	**POS**	**POS**	**POS**	**NEG**	**NEG**	**NEG**	**NEG**
**Washington-03_sample-117**	**POS**	**POS**	**POS**	**POS**	**POS**	**POS**	**NEG**	**NEG**	**NEG**	**NEG**
**Washington-04_sample-120**	**POS**	**POS**	**POS**	**POS**	**POS**	**POS**	**NEG**	**NEG**	**NEG**	**NEG**
**Washington-05_sample-505**	**POS**	**POS**	**POS**	**POS**	**NEG**	**POS**	**NEG**	**NEG**	**NEG**	**NEG**
**Washington-07_sample-611**	**POS**	**POS**	**POS**	**POS**	**NEG**	**POS**	**NEG**	**NEG**	**NEG**	**NEG**
**Washington-12_sample-807**	**POS**	**POS**	**POS**	**POS**	**AMB**	**POS**	**NEG**	**NEG**	**NEG**	**NEG**
**Washington-13_sample-811**	**POS**	**POS**	**POS**	**POS**	**AMB**	**POS**	**NEG**	**NEG**	**NEG**	**NEG**
**Washington-21_sample-2043**	**POS**	**POS**	**NEG**	**NEG**	**NEG**	**NEG**	**POS**	**POS**	**POS**	**POS**
**Washington-17_sample-2006**	**POS**	**POS**	**NEG**	**NEG**	**NEG**	**NEG**	**NEG**	**NEG**	**NEG**	**NEG**
**Washington-30_sample-5025_Subclone 1**	**POS**	**POS**	**NEG**	**NEG**	**NEG**	**NEG**	**NEG**	**NEG**	**NEG**	**NEG**

**From the previous study - for comparison:**										
**Washington-37_sample-48_ST22_Primate**	**POS**	**POS**	**POS**	**POS**	**NEG**	**POS**	**NEG**	**NEG**	**NEG**	**NEG**
**Washington-38_sample-06_ST22_Swine**	**POS**	**POS**	**POS**	**POS**	**NEG**	**POS**	**NEG**	**NEG**	**NEG**	**NEG**

ISOLATE	accessory gene regulator allele III				accessory gene regulator allele IV			haemolysin delta	alternate penicillin binding protein 2, defining MRSA
	**agrIII (total)**	**agrB-III**	**agrC-III**	**agrD-III**	**agrIV (total)**	**agrB-IV**	**agrC-IV**	***hld***	***mecA***
	median(agrB-III_11	median(agrB-III_11	median(agrC-III_11	median(agrD-III_1	median(agrB-IV_11	median(agrB-IV_11	median(agrC-IV_1	median(hld_11)	median(mecA_11;mecA-1,4)
**Washington-08_sample-640**	**NEG**	**NEG**	**NEG**	**NEG**	**NEG**	**NEG**	**NEG**	**POS**	**POS**
**Washington-09_sample-657**	**NEG**	**NEG**	**NEG**	**NEG**	**NEG**	**NEG**	**NEG**	**POS**	**POS**
**Washington-10_sample-801**	**NEG**	**NEG**	**NEG**	**NEG**	**NEG**	**NEG**	**NEG**	**POS**	**POS**
**Washington-11_sample-804**	**NEG**	**NEG**	**NEG**	**NEG**	**NEG**	**NEG**	**NEG**	**POS**	**POS**
**Washington-14_sample-1005**	**NEG**	**NEG**	**NEG**	**NEG**	**NEG**	**NEG**	**NEG**	**POS**	**POS**
**Washington-16_sample-1027**	**NEG**	**NEG**	**NEG**	**NEG**	**NEG**	**NEG**	**NEG**	**POS**	**POS**
**Washington-18_sample-2021**	**NEG**	**NEG**	**NEG**	**NEG**	**NEG**	**NEG**	**NEG**	**POS**	**POS**
**Washington-19_sample-2027**	**NEG**	**NEG**	**NEG**	**NEG**	**NEG**	**NEG**	**NEG**	**POS**	**POS**
**Washington-22_sample-2051**	**NEG**	**NEG**	**NEG**	**NEG**	**NEG**	**NEG**	**NEG**	**POS**	**POS**
**Washington-23_sample-3009**	**NEG**	**NEG**	**NEG**	**NEG**	**NEG**	**NEG**	**NEG**	**POS**	**POS**
**Washington-24_sample-3022**	**NEG**	**NEG**	**NEG**	**NEG**	**NEG**	**NEG**	**NEG**	**POS**	**POS**
**Washington-26_sample-4021**	**NEG**	**NEG**	**NEG**	**NEG**	**NEG**	**NEG**	**NEG**	**POS**	**POS**
**Washington-27_sample-4033**	**NEG**	**NEG**	**NEG**	**NEG**	**NEG**	**NEG**	**NEG**	**POS**	**POS**
**Washington-29_sample-5018**	**NEG**	**NEG**	**NEG**	**NEG**	**NEG**	**NEG**	**NEG**	**POS**	**POS**
**Washington-31_sample-5040**	**NEG**	**NEG**	**NEG**	**NEG**	**NEG**	**NEG**	**NEG**	**POS**	**POS**
**Washington-30_sample 5025_Subclone 2**	**NEG**	**NEG**	**NEG**	**NEG**	**AMB**	**POS**	**NEG**	**POS**	**POS**
**Washington-32_sample-21**	**NEG**	**NEG**	**NEG**	**NEG**	**NEG**	**NEG**	**NEG**	**POS**	**POS**
**Washington-33_sample-22**	**NEG**	**NEG**	**NEG**	**NEG**	**NEG**	**NEG**	**NEG**	**POS**	**POS**
**Washington-34_sample-23**	**NEG**	**NEG**	**NEG**	**NEG**	**NEG**	**NEG**	**NEG**	**POS**	**POS**
**Washington-35_sample-24**	**NEG**	**NEG**	**NEG**	**NEG**	**NEG**	**NEG**	**NEG**	**POS**	**POS**
**Washington-36_sample-25**	**NEG**	**NEG**	**NEG**	**NEG**	**NEG**	**NEG**	**NEG**	**POS**	**POS**
**Washington-25_sample-4007**	**NEG**	**NEG**	**NEG**	**NEG**	**NEG**	**NEG**	**NEG**	**POS**	**POS**
**Washington-28_sample-5008**	**NEG**	**NEG**	**NEG**	**NEG**	**NEG**	**NEG**	**NEG**	**POS**	**POS**
**Washington-06_sample-556**	**POS**	**POS**	**POS**	**POS**	**NEG**	**NEG**	**NEG**	**POS**	**POS**
**Washington-15_sample-1007**	**POS**	**POS**	**POS**	**POS**	**NEG**	**NEG**	**NEG**	**POS**	**POS**
**Washington-20_sample-2039**	**NEG**	**NEG**	**NEG**	**NEG**	**POS**	**POS**	**POS**	**POS**	**POS**

ISOLATE	accessory gene regulator allele III				accessory gene regulator allele IV			haemolysin delta	alternate penicillin binding protein 2, defining MRSA
	**agrIII (total)**	**agrB-III**	**agrC-III**	**agrD-III**	**agrIV (total)**	**agrB-IV**	**agrC-IV**	***hld***	***mecA***
	median(agrB-III_1	median(agrB-III_1	median(agrC-III_1	median(agrD-III_1	median(agrB-IV_1	median(agrB-IV_1	median(agrC-IV_1	median(hld_11)	median(mecA_11;mecA-1,4)
**Washington-01_sample-106**	**NEG**	**NEG**	**NEG**	**NEG**	**NEG**	**NEG**	**NEG**	**POS**	**POS**
**Washington-02_sample-115**	**NEG**	**NEG**	**NEG**	**NEG**	**NEG**	**NEG**	**NEG**	**POS**	**POS**
**Washington-03_sample-117**	**NEG**	**NEG**	**NEG**	**NEG**	**NEG**	**NEG**	**NEG**	**POS**	**POS**
**Washington-04_sample-120**	**NEG**	**NEG**	**NEG**	**NEG**	**NEG**	**NEG**	**NEG**	**POS**	**POS**
**Washington-05_sample-505**	**NEG**	**NEG**	**NEG**	**NEG**	**NEG**	**NEG**	**NEG**	**POS**	**POS**
**Washington-07_sample-611**	**NEG**	**NEG**	**NEG**	**NEG**	**NEG**	**NEG**	**NEG**	**POS**	**POS**
**Washington-12_sample-807**	**NEG**	**NEG**	**NEG**	**NEG**	**NEG**	**NEG**	**NEG**	**POS**	**POS**
**Washington-13_sample-811**	**NEG**	**NEG**	**NEG**	**NEG**	**NEG**	**NEG**	**NEG**	**POS**	**POS**
**Washington-21_sample-2043**	**NEG**	**NEG**	**NEG**	**NEG**	**NEG**	**NEG**	**NEG**	**POS**	**POS**
**Washington-17_sample-2006**	**POS**	**POS**	**POS**	**POS**	**NEG**	**NEG**	**NEG**	**POS**	**POS**
**Washington-30_sample-5025_Subclone 1**	**POS**	**POS**	**POS**	**POS**	**NEG**	**NEG**	**NEG**	**POS**	**POS**
**From the previous study - for comparison:**									
**Washington-37_sample-48_ST22_Primate**	**NEG**	**NEG**	**NEG**	**NEG**	**NEG**	**NEG**	**NEG**	**POS**	**POS**
**Washington-38_sample-06_ST22_Swine**	**NEG**	**NEG**	**NEG**	**NEG**	**NEG**	**NEG**	**NEG**	**POS**	**POS**

ISOLATE	truncated signal transducer protein MecR1	glycerophosphoryl diester phosphodi-esterase, associated with *mecA*	cassette chromosome recombinase genes A/B-1		plasmin-sensitive surface protein	hypothetical protein from SCCmec elements	cassette ch recombinase
	**delta_mecR**	***ugpQ***	***ccrA-1***	***ccrB-1***	**plsSCC (COL)**	**Q9XB68-dcs**	**ccrA-2**
	median(hp_mecR_611)	median(hp_ugpQ_611)	median(hp_ccrA-1_	median(hp_ccrB-1_	median(hp_plsSCC_611)	median(hp_Q9XB68_611)	median(hp_ccrA-2_
**Washington-08_sample-640**	**POS**	**POS**	**NEG**	**NEG**	**NEG**	**POS**	**POS**
**Washington-09_sample-657**	**POS**	**POS**	**NEG**	**NEG**	**NEG**	**POS**	**POS**
**Washington-10_sample-801**	**POS**	**POS**	**NEG**	**NEG**	**NEG**	**POS**	**POS**
**Washington-11_sample-804**	**POS**	**POS**	**NEG**	**NEG**	**NEG**	**AMB**	**POS**
**Washington-14_sample-1005**	**POS**	**POS**	**NEG**	**NEG**	**NEG**	**POS**	**POS**
**Washington-16_sample-1027**	**POS**	**POS**	**NEG**	**NEG**	**NEG**	**POS**	**POS**
**Washington-18_sample-2021**	**POS**	**POS**	**NEG**	**NEG**	**NEG**	**AMB**	**POS**
**Washington-19_sample-2027**	**POS**	**POS**	**NEG**	**NEG**	**NEG**	**POS**	**POS**
**Washington-22_sample-2051**	**POS**	**POS**	**NEG**	**NEG**	**NEG**	**POS**	**POS**
**Washington-23_sample-3009**	**POS**	**POS**	**NEG**	**NEG**	**NEG**	**POS**	**POS**
**Washington-24_sample-3022**	**POS**	**POS**	**NEG**	**NEG**	**NEG**	**POS**	**POS**
**Washington-26_sample-4021**	**POS**	**POS**	**NEG**	**NEG**	**NEG**	**POS**	**POS**
**Washington-27_sample-4033**	**POS**	**POS**	**NEG**	**NEG**	**NEG**	**POS**	**POS**
**Washington-29_sample-5018**	**POS**	**POS**	**NEG**	**NEG**	**NEG**	**POS**	**POS**
**Washington-31_sample-5040**	**POS**	**POS**	**NEG**	**NEG**	**NEG**	**POS**	**POS**
**Washington-30_sample 5025_Subclone 2**	**POS**	**POS**	**NEG**	**NEG**	**NEG**	**POS**	**POS**
**Washington-32_sample-21**	**POS**	**POS**	**NEG**	**NEG**	**NEG**	**AMB**	**POS**
**Washington-33_sample-22**	**POS**	**POS**	**NEG**	**NEG**	**NEG**	**POS**	**POS**
**Washington-34_sample-23**	**POS**	**POS**	**NEG**	**NEG**	**NEG**	**POS**	**POS**
**Washington-35_sample-24**	**POS**	**POS**	**NEG**	**NEG**	**NEG**	**NEG**	**POS**
**Washington-36_sample-25**	**POS**	**POS**	**NEG**	**NEG**	**NEG**	**NEG**	**POS**
**Washington-25_sample-4007**	**POS**	**POS**	**NEG**	**NEG**	**NEG**	**POS**	**POS**
**Washington-28_sample-5008**	**POS**	**POS**	**NEG**	**NEG**	**NEG**	**AMB**	**POS**
**Washington-06_sample-556**	**NEG**	**POS**	**NEG**	**NEG**	**NEG**	**NEG**	**NEG**
**Washington-15_sample-1007**	**NEG**	**POS**	**NEG**	**NEG**	**NEG**	**NEG**	**NEG**
**Washington-20_sample-2039**	**NEG**	**POS**	**NEG**	**NEG**	**NEG**	**NEG**	**NEG**

ISOLATE	truncated signal transducer protein MecR1	glycerophosphoryl diester phosphodi-esterase, associated with *mecA*	cassette chromosome recombinase genes A/B-1		plasmin-sensitive surface protein	hypothetical protein from SCCmec elements	cassette ch recombinase
	**delta_mecR**	***ugpQ***	***ccrA-1***	***ccrB-1***	**plsSCC (COL)**	**Q9XB68-dcs**	**ccrA-2**
	median(hp_mecR_611)	median(hp_ugpQ_611)	median(hp_ccrA-1_	median(hp_ccrB-1_	median(hp_plsSCC_611)	median(hp_Q9XB68_611)	median(hp_ccrA-2_
**Washington-01_sample-106**	**POS**	**POS**	**NEG**	**NEG**	**NEG**	**POS**	**POS**
**Washington-02_sample-115**	**POS**	**POS**	**NEG**	**NEG**	**NEG**	**POS**	**POS**
**Washington-03_sample-117**	**POS**	**POS**	**NEG**	**NEG**	**NEG**	**POS**	**POS**
**Washington-04_sample-120**	**POS**	**POS**	**NEG**	**NEG**	**NEG**	**POS**	**POS**
**Washington-05_sample-505**	**POS**	**POS**	**NEG**	**NEG**	**NEG**	**POS**	**POS**
**Washington-07_sample-611**	**POS**	**POS**	**NEG**	**NEG**	**NEG**	**AMB**	**POS**
**Washington-12_sample-807**	**POS**	**POS**	**NEG**	**NEG**	**NEG**	**POS**	**POS**
**Washington-13_sample-811**	**POS**	**POS**	**NEG**	**NEG**	**NEG**	**POS**	**POS**
**Washington-21_sample-2043**	**NEG**	**POS**	**NEG**	**NEG**	**NEG**	**NEG**	**NEG**
**Washington-17_sample-2006**	**NEG**	**POS**	**NEG**	**NEG**	**NEG**	**NEG**	**NEG**
**Washington-30_sample-5025_Subclone 1**	**NEG**	**POS**	**NEG**	**NEG**	**NEG**	**NEG**	**NEG**
**From the previous study - for comparison:**							
**Washington-37_sample-48_ST22_Primate**	**POS**	**POS**	**NEG**	**NEG**	**NEG**	**NEG**	**POS**
**Washington-38_sample-06_ST22_Swine**	**POS**	**POS**	**NEG**	**NEG**	**NEG**	**AMB**	**POS**

ISOLATE	METHICILLIN RESISTANCE AND SCCmec TYPING							
	romosome genes A/B-2	potassium-trans-locating ATPase A, chain 2	potassium-transporting ATPase B,	potassium-trans-locating	sensor kinase protein	KDP operon transcrip-tional regulatory protein	methicillin-resistance regulatory protein	signal transducer protein MecR1
	ccrB-2	**kdpA-SCC**	**kdpB-SCC**	**kdpC-SCC**	**kdpD-SCC**	**kdpE-SCC**	***mecI***	***mecR***
	median(hp_ccrB-2_	median(hp_kdpA-SCC_611)	median(hp_kdpB-S	median(hp_kdpC-S	median(hp_kdpD-SCC	median(hp_kdpE-SCC_611)	median(hp_mecI_611)	median(hp_mecR_612)
**Washington-08_sample-640**	**POS**	**NEG**	**NEG**	**NEG**	**NEG**	**NEG**	**NEG**	**NEG**
**Washington-09_sample-657**	**POS**	**NEG**	**NEG**	**NEG**	**NEG**	**NEG**	**NEG**	**NEG**
**Washington-10_sample-801**	**POS**	**NEG**	**NEG**	**NEG**	**NEG**	**NEG**	**NEG**	**NEG**
**Washington-11_sample-804**	**POS**	**NEG**	**NEG**	**NEG**	**NEG**	**NEG**	**NEG**	**NEG**
**Washington-14_sample-1005**	**POS**	**NEG**	**NEG**	**NEG**	**NEG**	**NEG**	**NEG**	**NEG**
**Washington-16_sample-1027**	**POS**	**NEG**	**NEG**	**NEG**	**NEG**	**NEG**	**NEG**	**NEG**
**Washington-18_sample-2021**	**POS**	**NEG**	**NEG**	**NEG**	**NEG**	**NEG**	**NEG**	**NEG**
**Washington-19_sample-2027**	**POS**	**NEG**	**NEG**	**NEG**	**NEG**	**NEG**	**NEG**	**NEG**
**Washington-22_sample-2051**	**POS**	**NEG**	**NEG**	**NEG**	**NEG**	**NEG**	**NEG**	**NEG**
**Washington-23_sample-3009**	**POS**	**NEG**	**NEG**	**NEG**	**NEG**	**NEG**	**NEG**	**NEG**
**Washington-24_sample-3022**	**POS**	**NEG**	**NEG**	**NEG**	**NEG**	**NEG**	**NEG**	**NEG**
**Washington-26_sample-4021**	**POS**	**NEG**	**NEG**	**NEG**	**NEG**	**NEG**	**NEG**	**NEG**
**Washington-27_sample-4033**	**POS**	**NEG**	**NEG**	**NEG**	**NEG**	**NEG**	**NEG**	**NEG**
**Washington-29_sample-5018**	**POS**	**NEG**	**NEG**	**NEG**	**NEG**	**NEG**	**NEG**	**NEG**
**Washington-31_sample-5040**	**POS**	**NEG**	**NEG**	**NEG**	**NEG**	**NEG**	**NEG**	**NEG**
**Washington-30_sample 5025_Subclone 2**	**POS**	**NEG**	**NEG**	**NEG**	**NEG**	**NEG**	**NEG**	**NEG**
**Washington-32_sample-21**	**POS**	**NEG**	**NEG**	**NEG**	**NEG**	**NEG**	**NEG**	**NEG**
**Washington-33_sample-22**	**POS**	**NEG**	**NEG**	**NEG**	**NEG**	**NEG**	**NEG**	**NEG**
**Washington-34_sample-23**	**POS**	**NEG**	**NEG**	**NEG**	**NEG**	**NEG**	**NEG**	**NEG**
**Washington-35_sample-24**	**POS**	**NEG**	**NEG**	**NEG**	**NEG**	**NEG**	**NEG**	**NEG**
**Washington-36_sample-25**	**POS**	**NEG**	**NEG**	**NEG**	**NEG**	**NEG**	**NEG**	**NEG**
**Washington-25_sample-4007**	**POS**	**NEG**	**NEG**	**NEG**	**NEG**	**NEG**	**NEG**	**NEG**
**Washington-28_sample-5008**	**POS**	**NEG**	**NEG**	**NEG**	**NEG**	**NEG**	**NEG**	**NEG**
**Washington-06_sample-556**	**NEG**	**NEG**	**NEG**	**NEG**	**NEG**	**NEG**	**NEG**	**NEG**
**Washington-15_sample-1007**	**NEG**	**NEG**	**NEG**	**NEG**	**NEG**	**NEG**	**NEG**	**NEG**
**Washington-20_sample-2039**	**NEG**	**NEG**	**NEG**	**NEG**	**NEG**	**NEG**	**NEG**	**NEG**

ISOLATE	METHICILLIN RESISTANCE AND SCCmec TYPING							
	romosome genes A/B-2	potassium-trans-locating ATPase A, chain 2	potassium-transporting ATPase B,	potassium-trans-locating	sensor kinase protein	KDP operon transcrip-tional regulatory protein	methicillin-resistance regulatory protein	signal transducer protein MecR1
	ccrB-2	**kdpA-SCC**	**kdpB-SCC**	**kdpC-SCC**	**kdpD-SCC**	**kdpE-SCC**	***mecI***	***mecR***
	median(hp_ccrB-2_	median(hp_kdpA-SCC_611)	median(hp_kdpB-S	median(hp_kdpC-S	median(hp_kdpD-SCC	median(hp_kdpE-SCC_611)	median(hp_mecI_611)	median(hp_mecR_612)
**Washington-01_sample-106**	**POS**	**NEG**	**NEG**	**NEG**	**NEG**	**NEG**	**NEG**	**NEG**
**Washington-02_sample-115**	**POS**	**NEG**	**NEG**	**NEG**	**NEG**	**NEG**	**NEG**	**NEG**
**Washington-03_sample-117**	**POS**	**NEG**	**NEG**	**NEG**	**NEG**	**NEG**	**NEG**	**NEG**
**Washington-04_sample-120**	**POS**	**NEG**	**NEG**	**NEG**	**NEG**	**NEG**	**NEG**	**NEG**
**Washington-05_sample-505**	**POS**	**NEG**	**NEG**	**NEG**	**NEG**	**NEG**	**NEG**	**NEG**
**Washington-07_sample-611**	**POS**	**NEG**	**NEG**	**NEG**	**NEG**	**NEG**	**NEG**	**NEG**
**Washington-12_sample-807**	**POS**	**NEG**	**NEG**	**NEG**	**NEG**	**NEG**	**NEG**	**NEG**
**Washington-13_sample-811**	**POS**	**NEG**	**NEG**	**NEG**	**NEG**	**NEG**	**NEG**	**NEG**
**Washington-21_sample-2043**	**NEG**	**NEG**	**NEG**	**NEG**	**NEG**	**NEG**	**NEG**	**NEG**
**Washington-17_sample-2006**	**NEG**	**NEG**	**NEG**	**NEG**	**NEG**	**NEG**	**NEG**	**NEG**
**Washington-30_sample-5025_Subclone 1**	**NEG**	**NEG**	**NEG**	**NEG**	**NEG**	**NEG**	**NEG**	**NEG**

**From the previous study - for comparison:**								
**Washington-37_sample-48_ST22_Primate**	**POS**	**NEG**	**NEG**	**NEG**	**NEG**	**NEG**	**NEG**	**NEG**
**Washington-38_sample-06_ST22_Swine**	**POS**	**NEG**	**NEG**	**NEG**	**NEG**	**NEG**	**NEG**	**NEG**

ISOLATE	homolog of xylose repressor, SCCmec-elements	cassette chromosome recombinase genes A/B-3		mercury resistance operon		cassette chromosome recombinase genes "ccrAA" (hypothetical) and ccrC			cassette chromosome recombinase genes A/B-4	
	xylR	***ccrA-3***	***ccrB-3***	***merA***	***merB***	**ccrAA (MRSAZH4**	**ccrAA (MRSAZH4**	**ccrC (85-2082)**	**ccrA-4**	**ccrB-4**
	median(hp_xylR_611)	median(hp_ccrA-3_	median(hp_ccrB-3_	median(hp_merA_	median(hp_merB_	median(hp_ccrAA_	median(hp_ccrAA_	median(hp_ccrC_6	median(hp_ccrA-4_	median(hp_ccrB-4_
**Washington-08_sample-640**	**NEG**	**NEG**	**NEG**	**NEG**	**NEG**	**NEG**	**NEG**	**NEG**	**NEG**	**NEG**
**Washington-09_sample-657**	**NEG**	**NEG**	**NEG**	**NEG**	**NEG**	**NEG**	**NEG**	**NEG**	**NEG**	**NEG**
**Washington-10_sample-801**	**NEG**	**NEG**	**NEG**	**NEG**	**NEG**	**NEG**	**NEG**	**NEG**	**NEG**	**NEG**
**Washington-11_sample-804**	**NEG**	**NEG**	**NEG**	**NEG**	**NEG**	**NEG**	**NEG**	**NEG**	**NEG**	**NEG**
**Washington-14_sample-1005**	**NEG**	**NEG**	**NEG**	**NEG**	**NEG**	**NEG**	**NEG**	**NEG**	**NEG**	**NEG**
**Washington-16_sample-1027**	**NEG**	**NEG**	**NEG**	**NEG**	**NEG**	**NEG**	**NEG**	**NEG**	**NEG**	**NEG**
**Washington-18_sample-2021**	**NEG**	**NEG**	**NEG**	**NEG**	**NEG**	**NEG**	**NEG**	**NEG**	**NEG**	**NEG**
**Washington-19_sample-2027**	**NEG**	**NEG**	**NEG**	**NEG**	**NEG**	**NEG**	**NEG**	**NEG**	**NEG**	**NEG**
**Washington-22_sample-2051**	**NEG**	**NEG**	**NEG**	**NEG**	**NEG**	**NEG**	**NEG**	**NEG**	**NEG**	**NEG**
**Washington-23_sample-3009**	**NEG**	**NEG**	**NEG**	**NEG**	**NEG**	**NEG**	**NEG**	**NEG**	**NEG**	**NEG**
**Washington-24_sample-3022**	**NEG**	**NEG**	**NEG**	**NEG**	**NEG**	**NEG**	**NEG**	**NEG**	**NEG**	**NEG**
**Washington-26_sample-4021**	**NEG**	**NEG**	**NEG**	**NEG**	**NEG**	**NEG**	**NEG**	**NEG**	**NEG**	**NEG**
**Washington-27_sample-4033**	**NEG**	**NEG**	**NEG**	**NEG**	**NEG**	**NEG**	**NEG**	**NEG**	**NEG**	**NEG**
**Washington-29_sample-5018**	**NEG**	**NEG**	**NEG**	**NEG**	**NEG**	**NEG**	**NEG**	**NEG**	**NEG**	**NEG**
**Washington-31_sample-5040**	**NEG**	**NEG**	**NEG**	**NEG**	**NEG**	**NEG**	**NEG**	**NEG**	**NEG**	**NEG**
**Washington-30_sample 5025_Subclone 2**	**NEG**	**NEG**	**NEG**	**NEG**	**NEG**	**NEG**	**NEG**	**NEG**	**NEG**	**NEG**
**Washington-32_sample-21**	**NEG**	**NEG**	**NEG**	**NEG**	**NEG**	**NEG**	**NEG**	**NEG**	**NEG**	**NEG**
**Washington-33_sample-22**	**NEG**	**NEG**	**NEG**	**NEG**	**NEG**	**NEG**	**NEG**	**NEG**	**NEG**	**NEG**
**Washington-34_sample-23**	**NEG**	**NEG**	**NEG**	**NEG**	**NEG**	**NEG**	**NEG**	**NEG**	**NEG**	**NEG**
**Washington-35_sample-24**	**NEG**	**NEG**	**NEG**	**NEG**	**NEG**	**NEG**	**NEG**	**NEG**	**NEG**	**NEG**
**Washington-36_sample-25**	**NEG**	**NEG**	**NEG**	**NEG**	**NEG**	**NEG**	**NEG**	**NEG**	**NEG**	**NEG**
**Washington-25_sample-4007**	**NEG**	**NEG**	**NEG**	**NEG**	**NEG**	**NEG**	**NEG**	**NEG**	**NEG**	**NEG**
**Washington-28_sample-5008**	**NEG**	**NEG**	**NEG**	**NEG**	**NEG**	**NEG**	**NEG**	**NEG**	**NEG**	**NEG**
**Washington-06_sample-556**	**NEG**	**NEG**	**NEG**	**NEG**	**NEG**	**POS**	**POS**	**POS**	**NEG**	**NEG**
**Washington-15_sample-1007**	**NEG**	**NEG**	**NEG**	**NEG**	**NEG**	**POS**	**POS**	**POS**	**NEG**	**NEG**
**Washington-20_sample-2039**	**NEG**	**NEG**	**NEG**	**NEG**	**NEG**	**POS**	**POS**	**POS**	**NEG**	**NEG**

ISOLATE	homolog of xylose repressor, SCCmec-elements	cassette chromosome recombinase genes A/B-3		mercury resistance operon		cassette chromosome recombinase genes "ccrAA" (hypothetical) and ccrC			cassette chromosome recombinase genes A/B-4	
	xylR	***ccrA-3***	***ccrB-3***	***merA***	***merB***	**ccrAA (MRSAZH4**	**ccrAA (MRSAZH4**	**ccrC (85-2082)**	**ccrA-4**	**ccrB-4**
	median(hp_xylR_611)	median(hp_ccrA-3_	median(hp_ccrB-3_	median(hp_merA_	median(hp_merB_	median(hp_ccrAA_	median(hp_ccrAA_	median(hp_ccrC_6	median(hp_ccrA-4_	median(hp_ccrB-4_
**Washington-01_sample-106**	**NEG**	**NEG**	**NEG**	**NEG**	**NEG**	**NEG**	**NEG**	**NEG**	**NEG**	**NEG**
**Washington-02_sample-115**	**NEG**	**NEG**	**NEG**	**NEG**	**NEG**	**NEG**	**NEG**	**NEG**	**NEG**	**NEG**
**Washington-03_sample-117**	**NEG**	**NEG**	**NEG**	**NEG**	**NEG**	**NEG**	**NEG**	**NEG**	**NEG**	**NEG**
**Washington-04_sample-120**	**NEG**	**NEG**	**NEG**	**NEG**	**NEG**	**NEG**	**NEG**	**NEG**	**NEG**	**NEG**
**Washington-05_sample-505**	**NEG**	**NEG**	**NEG**	**NEG**	**NEG**	**NEG**	**NEG**	**NEG**	**NEG**	**NEG**
**Washington-07_sample-611**	**NEG**	**NEG**	**NEG**	**NEG**	**NEG**	**NEG**	**NEG**	**NEG**	**NEG**	**NEG**
**Washington-12_sample-807**	**NEG**	**NEG**	**NEG**	**NEG**	**NEG**	**NEG**	**NEG**	**NEG**	**NEG**	**NEG**
**Washington-13_sample-811**	**NEG**	**NEG**	**NEG**	**NEG**	**NEG**	**NEG**	**NEG**	**NEG**	**NEG**	**NEG**
**Washington-21_sample-2043**	**NEG**	**NEG**	**NEG**	**NEG**	**NEG**	**POS**	**POS**	**POS**	**NEG**	**NEG**
**Washington-17_sample-2006**	**NEG**	**NEG**	**NEG**	**NEG**	**NEG**	**POS**	**POS**	**POS**	**NEG**	**NEG**
**Washington-30_sample-5025_Subclone 1**	**NEG**	**NEG**	**NEG**	**NEG**	**NEG**	**POS**	**POS**	**POS**	**NEG**	**NEG**

From the previous study - for comparison:

**Washington-37_sample-48_ST22_Primate**	**NEG**	**NEG**	**NEG**	**NEG**	**NEG**	**NEG**	**NEG**	**NEG**	**NEG**	**NEG**
**Washington-38_sample-06_ST22_Swine**	**NEG**	**NEG**	**NEG**	**NEG**	**NEG**	**NEG**	**NEG**	**NEG**	**NEG**	**NEG**

ISOLATE			RESISTANCE : PENICILLINASE					
	SCC*mec* XI		β-lactamase	β lactamase repressor	β-lactamase regulatory protein	RESISTANCE : MLS-ANTIBIOTICS	rRNA methylase	rRNA methylase
	mecC	***blaZ* -SCCmec XI**	***blaZ***	***blaI***	***blaR***	***erm* (A)**	***erm* (B)**	***erm* (C)**
	median (17_mecA_	(hp_01_blaZ_M10)	median(hp_blaZ_6	median(hp_blaI_611)	median(hp_blaR_611;hp_blaR_6	median(ermA-9,4)	median(hp_ermB_611;hp_	median(ermC-8,1;ermC-
**Washington-08_sample-640**	**NEG**	**NEG**	**POS**	**POS**	**POS**	**NEG**	**NEG**	**POS**
**Washington-09_sample-657**	**NEG**	**NEG**	**POS**	**POS**	**AMB**	**NEG**	**NEG**	**POS**
**Washington-10_sample-801**	**NEG**	**NEG**	**POS**	**POS**	**AMB**	**NEG**	**NEG**	**NEG**
**Washington-11_sample-804**	**NEG**	**NEG**	**POS**	**POS**	**AMB**	**NEG**	**NEG**	**NEG**
**Washington-14_sample-1005**	**NEG**	**NEG**	**POS**	**POS**	**AMB**	**NEG**	**NEG**	**POS**
**Washington-16_sample-1027**	**NEG**	**NEG**	**POS**	**POS**	**AMB**	**NEG**	**NEG**	**POS**
**Washington-18_sample-2021**	**NEG**	**NEG**	**POS**	**POS**	**AMB**	**NEG**	**NEG**	**POS**
**Washington-19_sample-2027**	**NEG**	**NEG**	**POS**	**POS**	**POS**	**NEG**	**NEG**	**POS**
**Washington-22_sample-2051**	**NEG**	**NEG**	**POS**	**POS**	**POS**	**NEG**	**NEG**	**POS**
**Washington-23_sample-3009**	**NEG**	**NEG**	**POS**	**POS**	**POS**	**NEG**	**NEG**	**POS**
**Washington-24_sample-3022**	**NEG**	**NEG**	**POS**	**POS**	**AMB**	**NEG**	**NEG**	**POS**
**Washington-26_sample-4021**	**NEG**	**NEG**	**POS**	**POS**	**AMB**	**NEG**	**NEG**	**NEG**
**Washington-27_sample-4033**	**NEG**	**NEG**	**POS**	**POS**	**AMB**	**NEG**	**NEG**	**POS**
**Washington-29_sample-5018**	**NEG**	**NEG**	**POS**	**POS**	**AMB**	**NEG**	**NEG**	**NEG**
**Washington-31_sample-5040**	**NEG**	**NEG**	**POS**	**POS**	**AMB**	**NEG**	**NEG**	**NEG**
**Washington-30_sample 5025_Subclone 2**	**NEG**	**NEG**	**POS**	**POS**	**POS**	**NEG**	**NEG**	**POS**
**Washington-32_sample-21**	**NEG**	**NEG**	**POS**	**POS**	**AMB**	**NEG**	**NEG**	**POS**
**Washington-33_sample-22**	**NEG**	**NEG**	**POS**	**POS**	**AMB**	**NEG**	**NEG**	**POS**
**Washington-34_sample-23**	**NEG**	**NEG**	**POS**	**POS**	**AMB**	**NEG**	**NEG**	**POS**
**Washington-35_sample-24**	**NEG**	**NEG**	**POS**	**AMB**	**AMB**	**NEG**	**NEG**	**NEG**
**Washington-36_sample-25**	**NEG**	**NEG**	**POS**	**POS**	**AMB**	**NEG**	**NEG**	**POS**
**Washington-25_sample-4007**	**NEG**	**NEG**	**POS**	**POS**	**AMB**	**NEG**	**NEG**	**NEG**
**Washington-28_sample-5008**	**NEG**	**NEG**	**POS**	**POS**	**AMB**	**NEG**	**NEG**	**NEG**
**Washington-06_sample-556**	**NEG**	**NEG**	**POS**	**POS**	**POS**	**NEG**	**NEG**	**POS**
**Washington-15_sample-1007**	**NEG**	**NEG**	**POS**	**POS**	**POS**	**NEG**	**NEG**	**POS**
**Washington-20_sample-2039**	**NEG**	**NEG**	**NEG**	**NEG**	**NEG**	**NEG**	**NEG**	**POS**

ISOLATE			RESISTANCE : PENICILLINASE					
	SCC*mec* XI		β-lactamase	β lactamase repressor	β-lactamase regulatory protein	RESISTANCE : MLS-ANTIBIOTICS	rRNA methylase	rRNA methylase
	mecC	***blaZ* -SCCmec XI**	***blaZ***	***blaI***	***blaR***	***erm* (A)**	***erm* (B)**	***erm* (C)**
	median (17_mecA_	(hp_01_blaZ_M10)	median(hp_blaZ_6	median(hp_blaI_611)	median(hp_blaR_611;hp_blaR_6	median(ermA-9,4)	median(hp_ermB_611;hp_	median(ermC-8,1;ermC-
**Washington-01_sample-106**	**NEG**	**NEG**	**POS**	**POS**	**POS**	**NEG**	**NEG**	**NEG**
**Washington-02_sample-115**	**NEG**	**NEG**	**POS**	**POS**	**POS**	**NEG**	**NEG**	**NEG**
**Washington-03_sample-117**	**NEG**	**NEG**	**POS**	**POS**	**POS**	**NEG**	**NEG**	**NEG**
**Washington-04_sample-120**	**NEG**	**NEG**	**POS**	**POS**	**POS**	**NEG**	**NEG**	**NEG**
**Washington-05_sample-505**	**NEG**	**NEG**	**POS**	**POS**	**POS**	**NEG**	**NEG**	**NEG**
**Washington-07_sample-611**	**NEG**	**NEG**	**POS**	**POS**	**POS**	**NEG**	**NEG**	**NEG**
**Washington-12_sample-807**	**NEG**	**NEG**	**POS**	**POS**	**POS**	**NEG**	**NEG**	**NEG**
**Washington-13_sample-811**	**NEG**	**NEG**	**POS**	**POS**	**POS**	**NEG**	**NEG**	**NEG**
**Washington-21_sample-2043**	**NEG**	**NEG**	**POS**	**POS**	**POS**	**NEG**	**NEG**	**NEG**
**Washington-17_sample-2006**	**NEG**	**NEG**	**POS**	**POS**	**POS**	**NEG**	**NEG**	**POS**
**Washington-30_sample-5025_Subclone 1**	**NEG**	**NEG**	**POS**	**POS**	**POS**	**NEG**	**NEG**	**POS**

**From the previous study - for comparison:**								
**Washington-37_sample-48_ST22_Primate**	**NEG**	**NEG**	**POS**	**POS**	**AMB**	**NEG**	**NEG**	**POS**
**Washington-38_sample-06_ST22_Swine**	**NEG**	**NEG**	**POS**	**POS**	**AMB**	**NEG**	**NEG**	**POS**

ISOLATE	RESISTANCE : MLS-ANTIBIOTICS							
	Lincosamid-resistance	efflux of erythromycin	macrolide efflux	probable lysylphos-phatidyl-glycerol synthetase	virginia-mycin A acetyltransfer	acetyl-transferase inactivating	ATP binding protein, streptogramin-A-resistance	
	lnu **(A)**	***msr*** **(A)**	***mef*** **(A)**	***mph*** **(C)**	***vat*** **(A)**	***vat*** **(B)**	***vga*** **(A)**	***vga*** **(A)**
	median(linA-19,2;linA-19,3)	median(15,3-msrA)	median(hp_mefA_611;h	median(hp_mpbBM_611;hp_mpbBM_612)	median(15,3-vatA)	median(16,3-vatB)	median(17,3-vga)	median(18,3-vgaA)
**Washington-08_sample-640**	**NEG**	**NEG**	**NEG**	**NEG**	**NEG**	**NEG**	**NEG**	**NEG**
**Washington-09_sample-657**	**NEG**	**NEG**	**NEG**	**NEG**	**NEG**	**NEG**	**NEG**	**NEG**
**Washington-10_sample-801**	**NEG**	**NEG**	**NEG**	**NEG**	**NEG**	**NEG**	**NEG**	**NEG**
**Washington-11_sample-804**	**NEG**	**NEG**	**NEG**	**NEG**	**NEG**	**NEG**	**NEG**	**NEG**
**Washington-14_sample-1005**	**NEG**	**NEG**	**NEG**	**NEG**	**NEG**	**NEG**	**NEG**	**NEG**
**Washington-16_sample-1027**	**NEG**	**NEG**	**NEG**	**NEG**	**NEG**	**NEG**	**NEG**	**NEG**
**Washington-18_sample-2021**	**NEG**	**NEG**	**NEG**	**NEG**	**NEG**	**NEG**	**NEG**	**NEG**
**Washington-19_sample-2027**	**NEG**	**NEG**	**NEG**	**NEG**	**NEG**	**NEG**	**NEG**	**NEG**
**Washington-22_sample-2051**	**NEG**	**NEG**	**NEG**	**NEG**	**NEG**	**NEG**	**NEG**	**NEG**
**Washington-23_sample-3009**	**NEG**	**NEG**	**NEG**	**NEG**	**NEG**	**NEG**	**NEG**	**NEG**
**Washington-24_sample-3022**	**NEG**	**NEG**	**NEG**	**NEG**	**NEG**	**NEG**	**NEG**	**NEG**
**Washington-26_sample-4021**	**NEG**	**NEG**	**NEG**	**NEG**	**NEG**	**NEG**	**NEG**	**NEG**
**Washington-27_sample-4033**	**NEG**	**NEG**	**NEG**	**NEG**	**NEG**	**NEG**	**NEG**	**NEG**
**Washington-29_sample-5018**	**NEG**	**NEG**	**NEG**	**NEG**	**NEG**	**NEG**	**NEG**	**NEG**
**Washington-31_sample-5040**	**NEG**	**NEG**	**NEG**	**NEG**	**NEG**	**NEG**	**NEG**	**NEG**
**Washington-30_sample 5025_Subclone 2**	**NEG**	**NEG**	**NEG**	**NEG**	**NEG**	**NEG**	**NEG**	**NEG**
**Washington-32_sample-21**	**NEG**	**NEG**	**NEG**	**NEG**	**NEG**	**NEG**	**NEG**	**NEG**
**Washington-33_sample-22**	**NEG**	**NEG**	**NEG**	**NEG**	**NEG**	**NEG**	**NEG**	**NEG**
**Washington-34_sample-23**	**NEG**	**NEG**	**NEG**	**NEG**	**NEG**	**NEG**	**NEG**	**NEG**
**Washington-35_sample-24**	**NEG**	**NEG**	**NEG**	**NEG**	**NEG**	**NEG**	**NEG**	**NEG**
**Washington-36_sample-25**	**NEG**	**NEG**	**NEG**	**NEG**	**NEG**	**NEG**	**NEG**	**NEG**
**Washington-25_sample-4007**	**NEG**	**NEG**	**NEG**	**NEG**	**NEG**	**NEG**	**NEG**	**NEG**
**Washington-28_sample-5008**	**NEG**	**NEG**	**NEG**	**NEG**	**NEG**	**NEG**	**NEG**	**NEG**
**Washington-06_sample-556**	**NEG**	**POS**	**NEG**	**POS**	**NEG**	**NEG**	**NEG**	**NEG**
**Washington-15_sample-1007**	**NEG**	**NEG**	**NEG**	**NEG**	**NEG**	**NEG**	**NEG**	**NEG**
**Washington-20_sample-2039**	**NEG**	**NEG**	**NEG**	**NEG**	**NEG**	**NEG**	**NEG**	**NEG**

ISOLATE	RESISTANCE : MLS-ANTIBIOTICS							
	Lincosamid-resistance	efflux of erythromycin	macrolide efflux	probable lysylphos-phatidyl-glycerol synthetase	virginia-mycin A acetyltransfer	acetyl-transferase inactivating	ATP binding protein, streptogramin-A-resistance	
	***lnu* (A)**	***msr* (A)**	***mef* (A)**	***mph* (C)**	***vat* (A)**	***vat* (B)**	***vga* (A)**	***vga* (A)**
	median(linA-19,2;linA-19,3)	median(15,3-msrA)	median(hp_mefA_611;h	median(hp_mpbBM_611;hp_mpbBM_612)	median(15,3-vatA)	median(16,3-vatB)	median(17,3-vga)	median(18,3-vgaA)
**Washington-01_sample-106**	**NEG**	**NEG**	**NEG**	**NEG**	**NEG**	**NEG**	**NEG**	**NEG**
**Washington-02_sample-115**	**NEG**	**NEG**	**NEG**	**NEG**	**NEG**	**NEG**	**NEG**	**NEG**
**Washington-03_sample-117**	**NEG**	**NEG**	**NEG**	**NEG**	**NEG**	**NEG**	**NEG**	**NEG**
**Washington-04_sample-120**	**NEG**	**NEG**	**NEG**	**NEG**	**NEG**	**NEG**	**NEG**	**NEG**
**Washington-05_sample-505**	**NEG**	**NEG**	**NEG**	**NEG**	**NEG**	**NEG**	**NEG**	**NEG**
**Washington-07_sample-611**	**NEG**	**NEG**	**NEG**	**NEG**	**NEG**	**NEG**	**NEG**	**NEG**
**Washington-12_sample-807**	**NEG**	**NEG**	**NEG**	**NEG**	**NEG**	**NEG**	**NEG**	**NEG**
**Washington-13_sample-811**	**NEG**	**NEG**	**NEG**	**NEG**	**NEG**	**NEG**	**NEG**	**NEG**
**Washington-21_sample-2043**	**NEG**	**POS**	**NEG**	**POS**	**NEG**	**NEG**	**NEG**	**NEG**
**Washington-17_sample-2006**	**NEG**	**NEG**	**NEG**	**NEG**	**NEG**	**NEG**	**NEG**	**NEG**
**Washington-30_sample-5025_Subclone 1**	**NEG**	**NEG**	**NEG**	**NEG**	**NEG**	**NEG**	**NEG**	**NEG**
**From the previous study - for comparison:**								
**Washington-37_sample-48_ST22_Primate**	**NEG**	**NEG**	**NEG**	**NEG**	**NEG**	**NEG**	**NEG**	**NEG**
**Washington-38_sample-06_ST22_Swine**	**NEG**	**NEG**	**NEG**	**NEG**	**NEG**	**NEG**	**NEG**	**NEG**

ISOLATE		RESISTANCE : AMINOGLYSOSIDES						
	virginiamycin hydrolase	bifunctional enzyme Aac/Aph, GM resistance	aminoglycoside ADP-transferase,tobramycin resistance	3'5'-aminoglycoside phospho-transferase, neo/ kanamycin	streptothricine-acetyltransferase	dihydrofolate reductase type 1	fusidic acid resistence	hypothetical protein: fusidic acid resistance
	vgb **(A)**	***aacA-aphD***	***aadD***	***aphA3***	***sat***	***dfrA***	***far1***	**Q6GD50 (*fusC*)**
	median(19,2-vgb)	median(aacA-aphD-10,4)	median(1,2-aadD)	median(aphA-3-18,3)	median(sat-17,2;sat	median(2,1-dfrA;df	median(far1_10)	median(hp_Q6GD5
**Washington-08_sample-640**	**NEG**	**POS**	**NEG**	**NEG**	**NEG**	**POS**	**NEG**	**NEG**
**Washington-09_sample-657**	**NEG**	**POS**	**NEG**	**NEG**	**NEG**	**POS**	**NEG**	**NEG**
**Washington-10_sample-801**	**NEG**	**POS**	**NEG**	**NEG**	**NEG**	**POS**	**NEG**	**NEG**
**Washington-11_sample-804**	**NEG**	**POS**	**NEG**	**NEG**	**NEG**	**POS**	**NEG**	**NEG**
**Washington-14_sample-1005**	**NEG**	**POS**	**NEG**	**NEG**	**NEG**	**POS**	**NEG**	**NEG**
**Washington-16_sample-1027**	**NEG**	**POS**	**NEG**	**NEG**	**NEG**	**POS**	**NEG**	**NEG**
**Washington-18_sample-2021**	**NEG**	**POS**	**NEG**	**NEG**	**NEG**	**POS**	**NEG**	**NEG**
**Washington-19_sample-2027**	**NEG**	**POS**	**NEG**	**NEG**	**NEG**	**POS**	**NEG**	**NEG**
**Washington-22_sample-2051**	**NEG**	**POS**	**NEG**	**NEG**	**NEG**	**POS**	**NEG**	**NEG**
**Washington-23_sample-3009**	**NEG**	**POS**	**NEG**	**NEG**	**NEG**	**POS**	**NEG**	**NEG**
**Washington-24_sample-3022**	**NEG**	**POS**	**NEG**	**NEG**	**NEG**	**POS**	**NEG**	**NEG**
**Washington-26_sample-4021**	**NEG**	**POS**	**NEG**	**NEG**	**NEG**	**POS**	**NEG**	**NEG**
**Washington-27_sample-4033**	**NEG**	**POS**	**NEG**	**NEG**	**NEG**	**POS**	**NEG**	**NEG**
**Washington-29_sample-5018**	**NEG**	**POS**	**NEG**	**NEG**	**NEG**	**POS**	**NEG**	**NEG**
**Washington-31_sample-5040**	**NEG**	**POS**	**NEG**	**NEG**	**NEG**	**POS**	**NEG**	**NEG**
**Washington-30_sample 5025_Subclone 2**	**NEG**	**POS**	**NEG**	**NEG**	**NEG**	**POS**	**NEG**	**NEG**
**Washington-32_sample-21**	**NEG**	**POS**	**NEG**	**NEG**	**NEG**	**POS**	**NEG**	**NEG**
**Washington-33_sample-22**	**NEG**	**POS**	**NEG**	**NEG**	**NEG**	**POS**	**NEG**	**NEG**
**Washington-34_sample-23**	**NEG**	**POS**	**NEG**	**NEG**	**NEG**	**POS**	**NEG**	**NEG**
**Washington-35_sample-24**	**NEG**	**POS**	**NEG**	**NEG**	**NEG**	**POS**	**NEG**	**NEG**
**Washington-36_sample-25**	**NEG**	**POS**	**NEG**	**NEG**	**NEG**	**POS**	**NEG**	**NEG**
**Washington-25_sample-4007**	**NEG**	**POS**	**NEG**	**NEG**	**NEG**	**POS**	**NEG**	**NEG**
**Washington-28_sample-5008**	**NEG**	**POS**	**POS**	**NEG**	**NEG**	**POS**	**NEG**	**NEG**
**Washington-06_sample-556**	**NEG**	**POS**	**NEG**	**POS**	**POS**	**NEG**	**NEG**	**NEG**
**Washington-15_sample-1007**	**NEG**	**POS**	**NEG**	**NEG**	**NEG**	**NEG**	**NEG**	**NEG**
**Washington-20_sample-2039**	**NEG**	**POS**	**NEG**	**NEG**	**NEG**	**NEG**	**NEG**	**NEG**

ISOLATE		RESISTANCE : AMINOGLYSOSIDES						
	virginiamycin hydrolase	bifunctional enzyme Aac/Aph, GM resistance	aminoglycoside ADP-transferase,tobramycin resistance	3'5'-aminoglycoside phospho-transferase, neo/ kanamycin	streptothricine-acetyltransferase	dihydrofolate reductase type 1	fusidic acid resistence	hypothetical protein: fusidic acid resistance
	vgb **(A)**	***aacA-aphD***	***aadD***	***aphA3***	***sat***	***dfrA***	***far1***	**Q6GD50 (*fusC*)**
	median(19,2-vgb)	median(aacA-aphD-10,4)	median(1,2-aadD)	median(aphA-3-18,3)	median(sat-17,2;sat	median(2,1-dfrA;df	median(far1_10)	median(hp_Q6GD5
**Washington-01_sample-106**	**NEG**	**NEG**	**NEG**	**NEG**	**NEG**	**NEG**	**NEG**	**NEG**
**Washington-02_sample-115**	**NEG**	**NEG**	**NEG**	**NEG**	**NEG**	**NEG**	**NEG**	**NEG**
**Washington-03_sample-117**	**NEG**	**NEG**	**NEG**	**NEG**	**NEG**	**NEG**	**NEG**	**NEG**
**Washington-04_sample-120**	**NEG**	**NEG**	**NEG**	**NEG**	**NEG**	**NEG**	**NEG**	**NEG**
**Washington-05_sample-505**	**NEG**	**NEG**	**NEG**	**NEG**	**NEG**	**NEG**	**NEG**	**NEG**
**Washington-07_sample-611**	**NEG**	**NEG**	**NEG**	**NEG**	**NEG**	**NEG**	**NEG**	**NEG**
**Washington-12_sample-807**	**NEG**	**NEG**	**NEG**	**NEG**	**NEG**	**NEG**	**NEG**	**NEG**
**Washington-13_sample-811**	**NEG**	**NEG**	**NEG**	**NEG**	**NEG**	**NEG**	**NEG**	**NEG**
**Washington-21_sample-2043**	**NEG**	**POS**	**NEG**	**POS**	**POS**	**NEG**	**NEG**	**NEG**
**Washington-17_sample-2006**	**NEG**	**POS**	**NEG**	**NEG**	**NEG**	**NEG**	**NEG**	**NEG**
**Washington-30_sample-5025_Subclone 1**	**NEG**	**POS**	**NEG**	**NEG**	**NEG**	**NEG**	**NEG**	**NEG**
**From the previous study - for comparison:**								
**Washington-37_sample-48_ST22_Primate**	**NEG**	**POS**	**NEG**	**NEG**	**NEG**	**POS**	**NEG**	**NEG**
**Washington-38_sample-06_ST22_Swine**	**NEG**	**POS**	**NEG**	**NEG**	**NEG**	**POS**	**NEG**	**NEG**

ISOLATE	RESISTANCE : MISCELLANEOUS GENES									
	mupirocin resistence	tetracycline	tetracycline	chloramphenicol acetyltransferase					23S rRNA methyltransferase	chlorampH/florfen exporter
	mupR	***tet* (K)**	***tet* (M)**	***cat***	***cat* (pC221)**	***cat* (pc223)**	***cat* (pMC524)**	***cat* (pSBK203R)**	***cfr***	***fexA***
	median(13,2-mupR)	median(tetK-12,3;t	median(tetM-11,3)	WENN((ZÄHLEN	median(hp_cat_613	median(hp_cat_611	median(hp_cat_612	median(hp_cat_615	median(hp_cfr_611	median(hp_fexA_611)
**Washington-08_sample-640**	**NEG**	**NEG**	**NEG**	**NEG**	**NEG**	**NEG**	**NEG**	**NEG**	**NEG**	**NEG**
**Washington-09_sample-657**	**NEG**	**NEG**	**NEG**	**NEG**	**NEG**	**NEG**	**NEG**	**NEG**	**NEG**	**NEG**
**Washington-10_sample-801**	**NEG**	**NEG**	**NEG**	**NEG**	**NEG**	**NEG**	**NEG**	**NEG**	**NEG**	**NEG**
**Washington-11_sample-804**	**NEG**	**NEG**	**NEG**	**NEG**	**NEG**	**NEG**	**NEG**	**NEG**	**NEG**	**NEG**
**Washington-14_sample-1005**	**NEG**	**NEG**	**NEG**	**NEG**	**NEG**	**NEG**	**NEG**	**NEG**	**NEG**	**NEG**
**Washington-16_sample-1027**	**NEG**	**NEG**	**NEG**	**NEG**	**NEG**	**NEG**	**NEG**	**NEG**	**NEG**	**NEG**
**Washington-18_sample-2021**	**NEG**	**NEG**	**NEG**	**NEG**	**NEG**	**NEG**	**NEG**	**NEG**	**NEG**	**NEG**
**Washington-19_sample-2027**	**NEG**	**NEG**	**NEG**	**NEG**	**NEG**	**NEG**	**NEG**	**NEG**	**NEG**	**NEG**
**Washington-22_sample-2051**	**NEG**	**NEG**	**NEG**	**NEG**	**NEG**	**NEG**	**NEG**	**NEG**	**NEG**	**NEG**
**Washington-23_sample-3009**	**NEG**	**NEG**	**NEG**	**NEG**	**NEG**	**NEG**	**NEG**	**NEG**	**NEG**	**NEG**
**Washington-24_sample-3022**	**NEG**	**NEG**	**NEG**	**NEG**	**NEG**	**NEG**	**NEG**	**NEG**	**NEG**	**NEG**
**Washington-26_sample-4021**	**NEG**	**NEG**	**NEG**	**NEG**	**NEG**	**NEG**	**NEG**	**NEG**	**NEG**	**NEG**
**Washington-27_sample-4033**	**NEG**	**NEG**	**NEG**	**NEG**	**NEG**	**NEG**	**NEG**	**NEG**	**NEG**	**NEG**
**Washington-29_sample-5018**	**NEG**	**NEG**	**NEG**	**NEG**	**NEG**	**NEG**	**NEG**	**NEG**	**NEG**	**NEG**
**Washington-31_sample-5040**	**NEG**	**NEG**	**NEG**	**NEG**	**NEG**	**NEG**	**NEG**	**NEG**	**NEG**	**NEG**
**Washington-30_sample 5025_Subclone 2**	**NEG**	**NEG**	**NEG**	**NEG**	**NEG**	**NEG**	**NEG**	**NEG**	**NEG**	**NEG**
**Washington-32_sample-21**	**NEG**	**NEG**	**NEG**	**NEG**	**NEG**	**NEG**	**NEG**	**NEG**	**NEG**	**NEG**
**Washington-33_sample-22**	**NEG**	**NEG**	**NEG**	**NEG**	**NEG**	**NEG**	**NEG**	**NEG**	**NEG**	**NEG**
**Washington-34_sample-23**	**NEG**	**NEG**	**NEG**	**NEG**	**NEG**	**NEG**	**NEG**	**NEG**	**NEG**	**NEG**
**Washington-35_sample-24**	**NEG**	**NEG**	**NEG**	**NEG**	**NEG**	**NEG**	**NEG**	**NEG**	**NEG**	**NEG**
**Washington-36_sample-25**	**NEG**	**NEG**	**NEG**	**NEG**	**NEG**	**NEG**	**NEG**	**NEG**	**NEG**	**NEG**
**Washington-25_sample-4007**	**NEG**	**NEG**	**NEG**	**NEG**	**NEG**	**NEG**	**NEG**	**NEG**	**NEG**	**NEG**
**Washington-28_sample-5008**	**NEG**	**NEG**	**NEG**	**NEG**	**NEG**	**NEG**	**NEG**	**NEG**	**NEG**	**NEG**
**Washington-06_sample-556**	**NEG**	**NEG**	**NEG**	**NEG**	**NEG**	**NEG**	**NEG**	**NEG**	**NEG**	**NEG**
**Washington-15_sample-1007**	**NEG**	**NEG**	**NEG**	**NEG**	**NEG**	**NEG**	**NEG**	**NEG**	**NEG**	**NEG**
**Washington-20_sample-2039**	**NEG**	**NEG**	**NEG**	**NEG**	**NEG**	**NEG**	**NEG**	**NEG**	**NEG**	**NEG**

ISOLATE	RESISTANCE : MISCELLANEOUS GENES									
	mupirocin resistence	tetracycline	tetracycline	chloramphenicol acetyltransferase					23S rRNA methyltransferase	chlorampH/florfen exporter
	mupR	***tet* (K)**	***tet* (M)**	***cat***	***cat* (pC221)**	***cat* (pc223)**	***cat* (pMC524)**	***cat* (pSBK203R)**	***cfr***	***fexA***
	median(13,2-mupR)	median(tetK-12,3;t	median(tetM-11,3)	WENN((ZÄHLEN	median(hp_cat_613	median(hp_cat_611	median(hp_cat_612	median(hp_cat_615	median(hp_cfr_611	median(hp_fexA_611)
**Washington-01_sample-106**	**NEG**	**NEG**	**NEG**	**NEG**	**NEG**	**NEG**	**NEG**	**NEG**	**NEG**	**NEG**
**Washington-02_sample-115**	**NEG**	**NEG**	**NEG**	**NEG**	**NEG**	**NEG**	**NEG**	**NEG**	**NEG**	**NEG**
**Washington-03_sample-117**	**NEG**	**NEG**	**NEG**	**NEG**	**NEG**	**NEG**	**NEG**	**NEG**	**NEG**	**NEG**
**Washington-04_sample-120**	**NEG**	**NEG**	**NEG**	**NEG**	**NEG**	**NEG**	**NEG**	**NEG**	**NEG**	**NEG**
**Washington-05_sample-505**	**NEG**	**NEG**	**NEG**	**NEG**	**NEG**	**NEG**	**NEG**	**NEG**	**NEG**	**NEG**
**Washington-07_sample-611**	**NEG**	**NEG**	**NEG**	**NEG**	**NEG**	**NEG**	**NEG**	**NEG**	**NEG**	**NEG**
**Washington-12_sample-807**	**NEG**	**NEG**	**NEG**	**NEG**	**NEG**	**NEG**	**NEG**	**NEG**	**NEG**	**NEG**
**Washington-13_sample-811**	**NEG**	**NEG**	**NEG**	**NEG**	**NEG**	**NEG**	**NEG**	**NEG**	**NEG**	**NEG**
**Washington-21_sample-2043**	**NEG**	**NEG**	**NEG**	**NEG**	**NEG**	**NEG**	**NEG**	**NEG**	**NEG**	**NEG**
**Washington-17_sample-2006**	**NEG**	**NEG**	**NEG**	**NEG**	**NEG**	**NEG**	**NEG**	**NEG**	**NEG**	**NEG**
**Washington-30_sample-5025_Subclone 1**	**NEG**	**NEG**	**NEG**	**NEG**	**NEG**	**NEG**	**NEG**	**NEG**	**NEG**	**NEG**

From the previous study - for comparison:

**Washington-37_sample-48_ST22_Primate**	**NEG**	**NEG**	**NEG**	**NEG**	**NEG**	**NEG**	**NEG**	**NEG**	**NEG**	**NEG**
**Washington-38_sample-06_ST22_Swine**	**NEG**	**NEG**	**NEG**	**NEG**	**NEG**	**NEG**	**NEG**	**NEG**	**NEG**	**NEG**

ISOLATE			RESISTANCE : EFFLUX SYSTEMS								RESISTAN
	metallothiol transferase		quaternary ammonium resistance	quaternary ammonium compound resistance protein C						Transport-/Efflux protein	vancomycin resistance gene
	fosB	*fosB* - plasmid	*qacA*	*qacC*	*qacC* (cons)	*qacC* (equine)	*qacC* (SA5)	*qacC* (Ssap)	*qacC* (ST94)	*sdrM*	*van* (A)
	median(hp_fosB_6	median(hp_fosB_6	median(hp_qacA_6	WENN((ZÄHLEN	median(hp_qacC_6	median(hp_qacC_6	median(hp_qacC_6	median(hp_qacC_6	median(hp_qacC_6	median(hp_tetEfflu	median(18,2-vanA)
Washington-08_sample-640	NEG	NEG	NEG	NEG	NEG	NEG	NEG	NEG	NEG	NEG	NEG
Washington-09_sample-657	NEG	NEG	NEG	NEG	NEG	NEG	NEG	NEG	NEG	NEG	NEG
Washington-10_sample-801	NEG	NEG	NEG	NEG	NEG	NEG	NEG	NEG	NEG	NEG	NEG
Washington-11_sample-804	NEG	NEG	NEG	NEG	NEG	NEG	NEG	NEG	NEG	NEG	NEG
Washington-14_sample-1005	NEG	NEG	NEG	NEG	NEG	NEG	NEG	NEG	NEG	NEG	NEG
Washington-16_sample-1027	NEG	NEG	NEG	NEG	NEG	NEG	NEG	NEG	NEG	NEG	NEG
Washington-18_sample-2021	NEG	NEG	NEG	NEG	NEG	NEG	NEG	NEG	NEG	NEG	NEG
Washington-19_sample-2027	NEG	NEG	NEG	NEG	NEG	NEG	NEG	NEG	NEG	NEG	NEG
Washington-22_sample-2051	NEG	NEG	NEG	NEG	NEG	NEG	NEG	NEG	NEG	NEG	NEG
Washington-23_sample-3009	NEG	NEG	NEG	NEG	NEG	NEG	NEG	NEG	NEG	NEG	NEG
Washington-24_sample-3022	NEG	NEG	NEG	NEG	NEG	NEG	NEG	NEG	NEG	NEG	NEG
Washington-26_sample-4021	NEG	NEG	NEG	NEG	NEG	NEG	NEG	NEG	NEG	NEG	NEG
Washington-27_sample-4033	NEG	NEG	NEG	NEG	NEG	NEG	NEG	NEG	NEG	NEG	NEG
Washington-29_sample-5018	NEG	NEG	NEG	NEG	NEG	NEG	NEG	NEG	NEG	NEG	NEG
Washington-31_sample-5040	NEG	NEG	NEG	NEG	NEG	NEG	NEG	NEG	NEG	NEG	NEG
Washington-30_sample 5025_Subclone 2	NEG	NEG	NEG	NEG	NEG	NEG	NEG	NEG	NEG	NEG	NEG
Washington-32_sample-21	NEG	NEG	NEG	NEG	NEG	NEG	NEG	NEG	NEG	NEG	NEG
Washington-33_sample-22	NEG	NEG	NEG	NEG	NEG	NEG	NEG	NEG	NEG	NEG	NEG
Washington-34_sample-23	NEG	NEG	NEG	NEG	NEG	NEG	NEG	NEG	NEG	NEG	NEG
Washington-35_sample-24	NEG	NEG	NEG	NEG	NEG	NEG	NEG	NEG	NEG	NEG	NEG
Washington-36_sample-25	NEG	NEG	NEG	NEG	NEG	NEG	NEG	NEG	NEG	NEG	NEG
Washington-25_sample-4007	NEG	NEG	NEG	NEG	NEG	NEG	NEG	NEG	NEG	NEG	NEG
Washington-28_sample-5008	NEG	NEG	NEG	NEG	NEG	NEG	NEG	NEG	NEG	NEG	NEG
Washington-06_sample-556	NEG	NEG	NEG	NEG	NEG	NEG	NEG	NEG	NEG	POS	NEG
Washington-15_sample-1007	NEG	NEG	NEG	NEG	NEG	NEG	NEG	NEG	NEG	POS	NEG
Washington-20_sample-2039	POS	NEG	NEG	NEG	NEG	NEG	NEG	NEG	NEG	POS	NEG

ISOLATE			RESISTANCE : EFFLUX SYSTEMS								RESISTAN
	metallothiol transferase		quaternary ammonium resistance	quaternary ammonium compound resistance protein C						Transport-/Efflux protein	vancomycin resistance gene
	fosB	*fosB* - plasmid	*qacA*	*qacC*	*qacC* (cons)	*qacC* (equine)	*qacC* (SA5)	*qacC* (Ssap)	*qacC* (ST94)	*sdrM*	*van* (A)
	median(hp_fosB_6	median(hp_fosB_6	median(hp_qacA_6	WENN((ZÄHLEN	median(hp_qacC_6	median(hp_qacC_6	median(hp_qacC_6	median(hp_qacC_6	median(hp_qacC_6	median(hp_tetEfflu	median(18,2-vanA)
**Washington-01_sample-106**	**POS**	**NEG**	**NEG**	**NEG**	**NEG**	**NEG**	**NEG**	**NEG**	**NEG**	**POS**	**NEG**
**Washington-02_sample-115**	**POS**	**NEG**	**NEG**	**NEG**	**NEG**	**NEG**	**NEG**	**NEG**	**NEG**	**POS**	**NEG**
**Washington-03_sample-117**	**POS**	**NEG**	**NEG**	**NEG**	**NEG**	**NEG**	**NEG**	**NEG**	**NEG**	**POS**	**NEG**
**Washington-04_sample-120**	**POS**	**NEG**	**NEG**	**NEG**	**NEG**	**NEG**	**NEG**	**NEG**	**NEG**	**POS**	**NEG**
**Washington-05_sample-505**	**POS**	**NEG**	**NEG**	**NEG**	**NEG**	**NEG**	**NEG**	**NEG**	**NEG**	**POS**	**NEG**
**Washington-07_sample-611**	**POS**	**NEG**	**NEG**	**NEG**	**NEG**	**NEG**	**NEG**	**NEG**	**NEG**	**POS**	**NEG**
**Washington-12_sample-807**	**POS**	**NEG**	**NEG**	**NEG**	**NEG**	**NEG**	**NEG**	**NEG**	**NEG**	**POS**	**NEG**
**Washington-13_sample-811**	**POS**	**NEG**	**NEG**	**NEG**	**NEG**	**NEG**	**NEG**	**NEG**	**NEG**	**POS**	**NEG**
**Washington-21_sample-2043**	**POS**	**NEG**	**NEG**	**NEG**	**NEG**	**NEG**	**NEG**	**NEG**	**NEG**	**POS**	**NEG**
**Washington-17_sample-2006**	**NEG**	**NEG**	**NEG**	**NEG**	**NEG**	**NEG**	**NEG**	**NEG**	**NEG**	**POS**	**NEG**
**Washington-30_sample-5025_Subclone 1**	**NEG**	**NEG**	**NEG**	**NEG**	**NEG**	**NEG**	**NEG**	**NEG**	**NEG**	**POS**	**NEG**

From the previous study - for comparison:

**Washington-37_sample-48_ST22_Primate**	**NEG**	**NEG**	**NEG**	**NEG**	**NEG**	**NEG**	**NEG**	**NEG**	**NEG**	**NEG**	**NEG**
**Washington-38_sample-06_ST22_Swine**	**NEG**	**NEG**	**NEG**	**NEG**	**NEG**	**NEG**	**NEG**	**NEG**	**NEG**	**NEG**	**NEG**

ISOLATE	CE : GLYCOPEPTIDES		VIRULENCE : TOX.SCHOCK.TOXIN								
	vancomycin resistance gene from enterococci and	teicoplanin resistance gene enterococci	toxic shock syndrome toxin 1			enterotoxin A	enterotoxin A, allele from strain 320E	enterotoxin A, allele from strain N315 = entero-toxin P	enterotoxin B	enterotoxin C	enterotoxin D
	van (B)	*van* (Z)	*tst1* (consensus)	*tst1* ("human"	*tst1* ("bovine"	*entA*	*entA* (320E)	*entA* (N315) / entP	*entB*	*entC*	*entD*
	median(19,3-vanB;	median(20,3-vanZ)	median(hp_tst_611	median(tst-1-16,2)	WENN((UND((hp_	median(3,2-entA;3,	median(entA-var2_	median(entA-var3_	median(4,1-entB;er	median(5,2-entC;5,	median(entD_11)
**Washington-08_sample-640**	**NEG**	**NEG**	**POS**	**POS**	**NEG**	**NEG**	**NEG**	**NEG**	**NEG**	**POS**	**NEG**
**Washington-09_sample-657**	**NEG**	**NEG**	**POS**	**POS**	**NEG**	**NEG**	**NEG**	**NEG**	**NEG**	**POS**	**NEG**
**Washington-10_sample-801**	**NEG**	**NEG**	**POS**	**POS**	**NEG**	**NEG**	**NEG**	**NEG**	**NEG**	**POS**	**NEG**
**Washington-11_sample-804**	**NEG**	**NEG**	**POS**	**POS**	**NEG**	**NEG**	**NEG**	**NEG**	**NEG**	**POS**	**NEG**
**Washington-14_sample-1005**	**NEG**	**NEG**	**POS**	**POS**	**NEG**	**NEG**	**NEG**	**NEG**	**NEG**	**POS**	**NEG**
**Washington-16_sample-1027**	**NEG**	**NEG**	**POS**	**POS**	**NEG**	**NEG**	**NEG**	**NEG**	**NEG**	**POS**	**NEG**
**Washington-18_sample-2021**	**NEG**	**NEG**	**POS**	**POS**	**NEG**	**NEG**	**NEG**	**NEG**	**NEG**	**POS**	**NEG**
**Washington-19_sample-2027**	**NEG**	**NEG**	**POS**	**POS**	**NEG**	**NEG**	**NEG**	**NEG**	**NEG**	**POS**	**NEG**
**Washington-22_sample-2051**	**NEG**	**NEG**	**POS**	**POS**	**NEG**	**NEG**	**NEG**	**NEG**	**NEG**	**POS**	**NEG**
**Washington-23_sample-3009**	**NEG**	**NEG**	**POS**	**POS**	**NEG**	**NEG**	**NEG**	**NEG**	**NEG**	**POS**	**NEG**
**Washington-24_sample-3022**	**NEG**	**NEG**	**POS**	**POS**	**NEG**	**NEG**	**NEG**	**NEG**	**NEG**	**POS**	**NEG**
**Washington-26_sample-4021**	**NEG**	**NEG**	**POS**	**POS**	**NEG**	**NEG**	**NEG**	**NEG**	**NEG**	**POS**	**NEG**
**Washington-27_sample-4033**	**NEG**	**NEG**	**POS**	**POS**	**NEG**	**NEG**	**NEG**	**NEG**	**NEG**	**POS**	**NEG**
**Washington-29_sample-5018**	**NEG**	**NEG**	**POS**	**POS**	**NEG**	**NEG**	**NEG**	**NEG**	**NEG**	**POS**	**NEG**
**Washington-31_sample-5040**	**NEG**	**NEG**	**POS**	**POS**	**NEG**	**NEG**	**NEG**	**NEG**	**NEG**	**POS**	**NEG**
**Washington-30_sample 5025_Subclone 2**	**NEG**	**NEG**	**POS**	**POS**	**NEG**	**NEG**	**NEG**	**NEG**	**NEG**	**POS**	**NEG**
**Washington-32_sample-21**	**NEG**	**NEG**	**POS**	**POS**	**NEG**	**NEG**	**NEG**	**NEG**	**NEG**	**POS**	**NEG**
**Washington-33_sample-22**	**NEG**	**NEG**	**POS**	**POS**	**NEG**	**NEG**	**NEG**	**NEG**	**NEG**	**POS**	**NEG**
**Washington-34_sample-23**	**NEG**	**NEG**	**POS**	**POS**	**NEG**	**NEG**	**NEG**	**NEG**	**NEG**	**POS**	**NEG**
**Washington-35_sample-24**	**NEG**	**NEG**	**POS**	**POS**	**NEG**	**NEG**	**NEG**	**NEG**	**NEG**	**POS**	**NEG**
**Washington-36_sample-25**	**NEG**	**NEG**	**POS**	**POS**	**NEG**	**NEG**	**NEG**	**NEG**	**NEG**	**POS**	**NEG**
**Washington-25_sample-4007**	**NEG**	**NEG**	**POS**	**POS**	**NEG**	**NEG**	**NEG**	**NEG**	**NEG**	**POS**	**NEG**
**Washington-28_sample-5008**	**NEG**	**NEG**	**NEG**	**NEG**	**NEG**	**NEG**	**NEG**	**NEG**	**NEG**	**NEG**	**NEG**
**Washington-06_sample-556**	**NEG**	**NEG**	**NEG**	**NEG**	**NEG**	**NEG**	**NEG**	**POS**	**NEG**	**NEG**	**NEG**
**Washington-15_sample-1007**	**NEG**	**NEG**	**NEG**	**NEG**	**NEG**	**POS**	**NEG**	**POS**	**NEG**	**NEG**	**NEG**
**Washington-20_sample-2039**	**NEG**	**NEG**	**NEG**	**NEG**	**NEG**	**NEG**	**NEG**	**NEG**	**NEG**	**NEG**	**NEG**

ISOLATE	CE : GLYCOPEPTIDES		VIRULENCE : TOX.SCHOCK.TOXIN								
	vancomycin resistance gene from enterococci and	teicoplanin resistance gene enterococci	toxic shock syndrome toxin 1			enterotoxin A	enterotoxin A, allele from strain 320E	enterotoxin A, allele from strain N315 = entero-toxin P	enterotoxin B	enterotoxin C	enterotoxin D
	van **(B)**	***van*** **(Z)**	***tst1*** **(consensus)**	***tst1*** **("human"**	***tst1*** **("bovine"**	***entA***	***entA*** **(320E)**	***entA*** **(N315) / entP**	***entB***	***entC***	***entD***
	median(19,3-vanB;	median(20,3-vanZ)	median(hp_tst_611	median(tst-1-16,2)	WENN((UND((hp	median(3,2-entA;3,	median(entA-var2_	median(entA-var3_	median(4,1-entB;er	median(5,2-entC;5,	median(entD_11)
**Washington-01_sample-106**	**NEG**	**NEG**	**NEG**	**NEG**	**NEG**	**NEG**	**NEG**	**NEG**	**POS**	**NEG**	**NEG**
**Washington-02_sample-115**	**NEG**	**NEG**	**NEG**	**NEG**	**NEG**	**NEG**	**NEG**	**NEG**	**POS**	**NEG**	**NEG**
**Washington-03_sample-117**	**NEG**	**NEG**	**NEG**	**NEG**	**NEG**	**NEG**	**NEG**	**NEG**	**POS**	**NEG**	**NEG**
**Washington-04_sample-120**	**NEG**	**NEG**	**NEG**	**NEG**	**NEG**	**NEG**	**NEG**	**NEG**	**POS**	**NEG**	**NEG**
**Washington-05_sample-505**	**NEG**	**NEG**	**NEG**	**NEG**	**NEG**	**NEG**	**NEG**	**NEG**	**POS**	**NEG**	**NEG**
**Washington-07_sample-611**	**NEG**	**NEG**	**NEG**	**NEG**	**NEG**	**NEG**	**NEG**	**NEG**	**POS**	**NEG**	**NEG**
**Washington-12_sample-807**	**NEG**	**NEG**	**NEG**	**NEG**	**NEG**	**NEG**	**NEG**	**NEG**	**POS**	**NEG**	**NEG**
**Washington-13_sample-811**	**NEG**	**NEG**	**POS**	**NEG**	**POS**	**NEG**	**NEG**	**NEG**	**POS**	**NEG**	**NEG**
**Washington-21_sample-2043**	**NEG**	**NEG**	**NEG**	**NEG**	**NEG**	**POS**	**NEG**	**NEG**	**NEG**	**POS**	**NEG**
**Washington-17_sample-2006**	**NEG**	**NEG**	**NEG**	**NEG**	**NEG**	**NEG**	**NEG**	**NEG**	**NEG**	**NEG**	**NEG**
**Washington-30_sample-5025_Subclone 1**	**NEG**	**NEG**	**NEG**	**NEG**	**NEG**	**NEG**	**NEG**	**NEG**	**NEG**	**NEG**	**NEG**
**From the previous study - for comparison:**											
**Washington-37_sample-48_ST22_Primate**	**NEG**	**NEG**	**POS**	**POS**	**NEG**	**NEG**	**NEG**	**NEG**	**NEG**	**POS**	**NEG**
**Washington-38_sample-06_ST22_Swine**	**NEG**	**NEG**	**POS**	**POS**	**NEG**	**NEG**	**NEG**	**NEG**	**NEG**	**POS**	**NEG**

ISOLATE	VIRULENCE : ENTEROTOXINS										
	enterotoxin E	enterotoxin G	enterotoxin H	enterotoxin I	enterotoxin J	enterotoxin K	enterotoxin L	enterotoxin M	enterotoxin N		enterotoxin O
	entE	***entG***	***entH***	***entI***	***entJ***	***entK***	***entL***	***entM***	***entN* (cons)**	***entN* (not RE122)**	***entO***
	median(entE_11)	median(entG_11)	median(entH_11)	median(entI_11)	median(entJ_11)	median(hp_entK_6	median(entL_11)	median(entM_11)	median(entN_611)	median(entN_11)	median(entO_11)
**Washington-08_sample-640**	**NEG**	**POS**	**NEG**	**POS**	**NEG**	**NEG**	**POS**	**POS**	**POS**	**POS**	**POS**
**Washington-09_sample-657**	**NEG**	**POS**	**NEG**	**POS**	**NEG**	**NEG**	**POS**	**POS**	**POS**	**POS**	**POS**
**Washington-10_sample-801**	**NEG**	**POS**	**NEG**	**POS**	**NEG**	**NEG**	**POS**	**POS**	**POS**	**POS**	**POS**
**Washington-11_sample-804**	**NEG**	**POS**	**NEG**	**POS**	**NEG**	**NEG**	**POS**	**POS**	**POS**	**POS**	**POS**
**Washington-14_sample-1005**	**NEG**	**POS**	**NEG**	**POS**	**NEG**	**NEG**	**POS**	**POS**	**POS**	**POS**	**POS**
**Washington-16_sample-1027**	**NEG**	**POS**	**NEG**	**POS**	**NEG**	**NEG**	**POS**	**POS**	**POS**	**POS**	**POS**
**Washington-18_sample-2021**	**NEG**	**POS**	**NEG**	**POS**	**NEG**	**NEG**	**POS**	**POS**	**POS**	**POS**	**POS**
**Washington-19_sample-2027**	**NEG**	**POS**	**NEG**	**POS**	**NEG**	**NEG**	**POS**	**POS**	**POS**	**POS**	**POS**
**Washington-22_sample-2051**	**NEG**	**POS**	**NEG**	**POS**	**NEG**	**NEG**	**POS**	**POS**	**POS**	**POS**	**POS**
**Washington-23_sample-3009**	**NEG**	**POS**	**NEG**	**POS**	**NEG**	**NEG**	**POS**	**POS**	**POS**	**POS**	**POS**
**Washington-24_sample-3022**	**NEG**	**POS**	**NEG**	**POS**	**NEG**	**NEG**	**POS**	**POS**	**POS**	**POS**	**POS**
**Washington-26_sample-4021**	**NEG**	**POS**	**NEG**	**POS**	**NEG**	**NEG**	**POS**	**POS**	**POS**	**POS**	**POS**
**Washington-27_sample-4033**	**NEG**	**POS**	**NEG**	**POS**	**NEG**	**NEG**	**POS**	**POS**	**POS**	**POS**	**POS**
**Washington-29_sample-5018**	**NEG**	**POS**	**NEG**	**POS**	**NEG**	**NEG**	**POS**	**POS**	**POS**	**POS**	**POS**
**Washington-31_sample-5040**	**NEG**	**POS**	**NEG**	**POS**	**NEG**	**NEG**	**POS**	**POS**	**POS**	**POS**	**POS**
**Washington-30_sample 5025_Subclone 2**	**NEG**	**POS**	**NEG**	**POS**	**NEG**	**NEG**	**POS**	**POS**	**POS**	**POS**	**POS**
**Washington-32_sample-21**	**NEG**	**POS**	**NEG**	**POS**	**NEG**	**NEG**	**POS**	**POS**	**POS**	**POS**	**AMB**
**Washington-33_sample-22**	**NEG**	**POS**	**NEG**	**POS**	**NEG**	**NEG**	**POS**	**POS**	**POS**	**POS**	**POS**
**Washington-34_sample-23**	**NEG**	**POS**	**NEG**	**POS**	**NEG**	**NEG**	**POS**	**POS**	**POS**	**POS**	**POS**
**Washington-35_sample-24**	**NEG**	**POS**	**NEG**	**POS**	**NEG**	**NEG**	**POS**	**POS**	**POS**	**POS**	**NEG**
**Washington-36_sample-25**	**NEG**	**POS**	**NEG**	**POS**	**NEG**	**NEG**	**POS**	**POS**	**POS**	**POS**	**AMB**
**Washington-25_sample-4007**	**NEG**	**POS**	**NEG**	**POS**	**NEG**	**NEG**	**POS**	**POS**	**POS**	**POS**	**POS**
**Washington-28_sample-5008**	**NEG**	**POS**	**NEG**	**POS**	**NEG**	**NEG**	**NEG**	**POS**	**POS**	**POS**	**POS**
**Washington-06_sample-556**	**NEG**	**NEG**	**NEG**	**NEG**	**NEG**	**POS**	**NEG**	**NEG**	**NEG**	**NEG**	**NEG**
**Washington-15_sample-1007**	**NEG**	**NEG**	**NEG**	**NEG**	**NEG**	**POS**	**NEG**	**NEG**	**NEG**	**NEG**	**NEG**
**Washington-20_sample-2039**	**NEG**	**POS**	**NEG**	**NEG**	**NEG**	**NEG**	**NEG**	**POS**	**POS**	**POS**	**POS**

ISOLATE	VIRULENCE : ENTEROTOXINS										
	enterotoxin E	enterotoxin G	enterotoxin H	enterotoxin I	enterotoxin J	enterotoxin K	enterotoxin L	enterotoxin M	enterotoxin N		enterotoxin O
	entE	***entG***	***entH***	***entI***	***entJ***	***entK***	***entL***	***entM***	***entN*** **(cons)**	***entN*** **(not RE122)**	***entO***
	median(entE_11)	median(entG_11)	median(entH_11)	median(entI_11)	median(entJ_11)	median(hp_entK_6	median(entL_11)	median(entM_11)	median(entN_611)	median(entN_11)	median(entO_11)
**Washington-01_sample-106**	NEG	POS	NEG	POS	NEG	POS	NEG	POS	POS	POS	POS
**Washington-02_sample-115**	NEG	POS	NEG	POS	NEG	POS	NEG	POS	POS	POS	POS
**Washington-03_sample-117**	NEG	POS	NEG	POS	NEG	POS	NEG	POS	POS	POS	POS
**Washington-04_sample-120**	NEG	POS	NEG	POS	NEG	POS	NEG	POS	POS	POS	POS
**Washington-05_sample-505**	NEG	POS	NEG	POS	NEG	POS	NEG	POS	POS	POS	POS
**Washington-07_sample-611**	NEG	POS	NEG	POS	NEG	POS	NEG	POS	POS	POS	POS
**Washington-12_sample-807**	NEG	POS	NEG	POS	NEG	POS	NEG	POS	POS	POS	POS
**Washington-13_sample-811**	NEG	POS	NEG	POS	NEG	POS	NEG	POS	POS	POS	POS
**Washington-21_sample-2043**	NEG	POS	NEG	POS	NEG	NEG	POS	POS	POS	POS	POS
**Washington-17_sample-2006**	NEG	NEG	NEG	NEG	NEG	POS	NEG	NEG	NEG	NEG	NEG
**Washington-30_sample-5025_Subclone 1**	NEG	NEG	NEG	NEG	NEG	POS	NEG	NEG	NEG	NEG	NEG

From the previous study - for comparison:

**Washington-37_sample-48_ST22_Primate**	NEG	POS	NEG	POS	NEG	NEG	POS	POS	POS	POS	NEG
**Washington-38_sample-06_ST22_Swine**	NEG	POS	NEG	POS	NEG	NEG	POS	POS	POS	POS	AMB

ISOLATE											VI
	egc cluster	enterotoxin Q	enterotoxin R	Enterotoxin U and/or Y	enterotoxin-like protein ORF CM14		haemolysin gamma/ leukocidin, component B	haemolysin gamma / leukocidin, component C		haemolysin γ, component A	Panton Valentine leukocidin F
	egc (total)	*entQ*	*entR*	*entU*	entCM14 probe1	entCM14 probe2	*lukF*	*lukS*	*lukS* (ST22+ST45	*hlgA*	*lukF-PV*
	;hp_entN_611;entN	median(hp_entQ_6	median(entR_11)	median(hp_entU_6	median(hp_entCM1	median(hp_entCM	median(lukF_10)	median(lukS_10)	median(hp_lukS-ST	median(hlgA_11)	median(lukF-PV_1
Washington-08_sample-640	POS	NEG	NEG	POS	NEG	NEG	NEG	NEG	POS	POS	POS
Washington-09_sample-657	POS	NEG	NEG	POS	NEG	NEG	NEG	NEG	POS	AMB	POS
Washington-10_sample-801	POS	NEG	NEG	POS	NEG	NEG	NEG	NEG	POS	AMB	POS
Washington-11_sample-804	POS	NEG	NEG	POS	NEG	NEG	NEG	NEG	POS	AMB	POS
Washington-14_sample-1005	POS	NEG	NEG	POS	NEG	NEG	NEG	NEG	POS	POS	POS
Washington-16_sample-1027	POS	NEG	NEG	POS	NEG	NEG	NEG	NEG	POS	POS	POS
Washington-18_sample-2021	POS	NEG	NEG	POS	NEG	NEG	NEG	NEG	POS	AMB	POS
Washington-19_sample-2027	POS	NEG	NEG	POS	NEG	NEG	NEG	NEG	POS	POS	POS
Washington-22_sample-2051	POS	NEG	NEG	POS	NEG	NEG	NEG	NEG	POS	POS	POS
Washington-23_sample-3009	POS	NEG	NEG	POS	NEG	NEG	NEG	NEG	POS	POS	POS
Washington-24_sample-3022	POS	NEG	NEG	POS	NEG	NEG	NEG	NEG	POS	AMB	POS
Washington-26_sample-4021	POS	NEG	NEG	POS	NEG	NEG	NEG	NEG	POS	AMB	POS
Washington-27_sample-4033	POS	NEG	NEG	POS	NEG	NEG	NEG	NEG	POS	POS	POS
Washington-29_sample-5018	POS	NEG	NEG	POS	NEG	NEG	NEG	NEG	POS	POS	POS
Washington-31_sample-5040	POS	NEG	NEG	POS	NEG	NEG	NEG	NEG	POS	POS	POS
Washington-30_sample 5025_Subclone 2	POS	NEG	NEG	POS	NEG	NEG	POS	POS	POS	POS	POS
Washington-32_sample-21	POS	NEG	NEG	POS	NEG	NEG	NEG	NEG	POS	AMB	POS
Washington-33_sample-22	POS	NEG	NEG	POS	NEG	NEG	NEG	NEG	POS	POS	POS
Washington-34_sample-23	POS	NEG	NEG	POS	NEG	NEG	NEG	NEG	POS	POS	POS
Washington-35_sample-24	POS	NEG	NEG	POS	NEG	NEG	NEG	NEG	POS	NEG	NEG
Washington-36_sample-25	POS	NEG	NEG	POS	NEG	NEG	NEG	NEG	POS	NEG	AMB
Washington-25_sample-4007	POS	NEG	NEG	POS	NEG	NEG	NEG	NEG	POS	POS	NEG
Washington-28_sample-5008	POS	NEG	NEG	POS	NEG	NEG	NEG	NEG	POS	AMB	POS
Washington-06_sample-556	NEG	POS	NEG	NEG	NEG	NEG	POS	POS	NEG	POS	NEG
Washington-15_sample-1007	NEG	POS	NEG	NEG	NEG	NEG	POS	POS	NEG	POS	POS
Washington-20_sample-2039	POS	NEG	NEG	POS	POS	POS	POS	POS	NEG	POS	NEG

ISOLATE											VI
	egc cluster	enterotoxin Q	enterotoxin R	Enterotoxin U and/or Y	enterotoxin-like protein ORF CM14		haemolysin gamma/ leukocidin, component B	haemolysin gamma / leukocidin, component C		haemolysin γ, component A	Panton Valentine leukocidin F
	egc (total)	*entQ*	*entR*	*entU*	entCM14 probe1	entCM14 probe2	*lukF*	*lukS*	*lukS* (ST22+ST45	*hlgA*	*lukF-PV*
	;hp_entN_611;entN	median(hp_entQ_6	median(entR_11)	median(hp_entU_6	median(hp_entCM1	median(hp_entCM1	median(lukF_10)	median(lukS_10)	median(hp_lukS-ST	median(hlgA_11)	median(lukF-PV_1
**Washington-01_sample-106**	**POS**	**POS**	**NEG**	**POS**	**NEG**	**NEG**	**POS**	**POS**	**NEG**	**POS**	**NEG**
**Washington-02_sample-115**	**POS**	**POS**	**NEG**	**POS**	**NEG**	**NEG**	**POS**	**POS**	**NEG**	**POS**	**NEG**
**Washington-03_sample-117**	**POS**	**POS**	**NEG**	**POS**	**NEG**	**NEG**	**POS**	**POS**	**NEG**	**POS**	**NEG**
**Washington-04_sample-120**	**POS**	**POS**	**NEG**	**POS**	**NEG**	**NEG**	**POS**	**POS**	**NEG**	**POS**	**NEG**
**Washington-05_sample-505**	**POS**	**POS**	**NEG**	**POS**	**NEG**	**NEG**	**POS**	**POS**	**NEG**	**POS**	**NEG**
**Washington-07_sample-611**	**POS**	**POS**	**NEG**	**POS**	**NEG**	**NEG**	**POS**	**POS**	**NEG**	**POS**	**NEG**
**Washington-12_sample-807**	**POS**	**POS**	**NEG**	**POS**	**NEG**	**NEG**	**POS**	**POS**	**NEG**	**POS**	**NEG**
**Washington-13_sample-811**	**POS**	**POS**	**NEG**	**POS**	**NEG**	**NEG**	**POS**	**POS**	**NEG**	**POS**	**NEG**
**Washington-21_sample-2043**	**POS**	**NEG**	**NEG**	**POS**	**POS**	**POS**	**POS**	**POS**	**NEG**	**POS**	**POS**
**Washington-17_sample-2006**	**NEG**	**POS**	**NEG**	**NEG**	**NEG**	**NEG**	**POS**	**POS**	**NEG**	**POS**	**NEG**
**Washington-30_sample-5025_Subclone 1**	**NEG**	**POS**	**NEG**	**NEG**	**NEG**	**NEG**	**POS**	**POS**	**POS**	**POS**	**NEG**

**From the previous study - for comparison:**											
**Washington-37_sample-48_ST22_Primate**	**POS**	**NEG**	**NEG**	**POS**	**NEG**	**NEG**	**NEG**	**NEG**	**POS**	**NEG**	**AMB**
**Washington-38_sample-06_ST22_Swine**	**POS**	**NEG**	**NEG**	**POS**	**NEG**	**NEG**	**NEG**	**NEG**	**POS**	**AMB**	**POS**

ISOLATE	RULENCE : HLG AND LEUKOCIDINS								VI		
	Panton Valentine leukocidin S	F component from hypothetical	S component from hypothetical	leukocidin D component	leukocidin E component	leukocidin/ haemolysin toxin family	leukocidin/haemolysin toxin family protein		putative membrane protein	haemolysin alpha	putative mem
	lukS-PV	lukF-PV (P83)	*lukM*	*lukD*	*lukE*	*lukX*	*lukY*	*lukY* (ST30+ST45)	*hl*	*hla*	*hlIII* (cons)
	median(lukS-PV_2	median(lukF-PV-P(	median(lukM_11)	median(lukD_11)	median(lukE_11)	median(lukX_11)	median(lukY-var1_	median(lukY-var2_	median(hl_11)	median(hla_11)	median(hp_hlIII_6
**Washington-08_sample-640**	POS	NEG	NEG	NEG	NEG	POS	NEG	NEG	POS	NEG	NEG
**Washington-09_sample-657**	POS	NEG	NEG	NEG	NEG	POS	NEG	NEG	POS	NEG	NEG
**Washington-10_sample-801**	POS	NEG	NEG	NEG	NEG	POS	NEG	NEG	POS	NEG	NEG
**Washington-11_sample-804**	POS	NEG	NEG	NEG	NEG	POS	NEG	NEG	POS	NEG	NEG
**Washington-14_sample-1005**	POS	NEG	NEG	NEG	NEG	POS	NEG	NEG	POS	NEG	NEG
**Washington-16_sample-1027**	POS	NEG	NEG	NEG	NEG	POS	NEG	NEG	POS	NEG	NEG
**Washington-18_sample-2021**	POS	NEG	NEG	NEG	NEG	POS	NEG	NEG	POS	NEG	NEG
**Washington-19_sample-2027**	POS	NEG	NEG	NEG	NEG	POS	POS	NEG	POS	NEG	NEG
**Washington-22_sample-2051**	POS	NEG	NEG	NEG	NEG	POS	AMB	NEG	POS	NEG	NEG
**Washington-23_sample-3009**	POS	NEG	NEG	NEG	NEG	POS	NEG	NEG	POS	NEG	NEG
**Washington-24_sample-3022**	POS	NEG	NEG	NEG	NEG	POS	NEG	NEG	POS	NEG	NEG
**Washington-26_sample-4021**	POS	NEG	NEG	NEG	NEG	POS	NEG	NEG	POS	NEG	NEG
**Washington-27_sample-4033**	POS	NEG	NEG	NEG	NEG	POS	NEG	NEG	POS	NEG	NEG
**Washington-29_sample-5018**	POS	NEG	NEG	NEG	NEG	POS	NEG	NEG	POS	NEG	NEG
**Washington-31_sample-5040**	POS	NEG	NEG	NEG	NEG	POS	NEG	NEG	POS	NEG	NEG
**Washington-30_sample 5025_Subclone 2**	POS	NEG	NEG	NEG	NEG	POS	POS	NEG	POS	POS	NEG
**Washington-32_sample-21**	POS	NEG	NEG	NEG	NEG	POS	NEG	NEG	POS	NEG	NEG
**Washington-33_sample-22**	POS	NEG	NEG	NEG	NEG	POS	NEG	NEG	POS	NEG	NEG
**Washington-34_sample-23**	POS	NEG	NEG	NEG	NEG	POS	NEG	NEG	POS	NEG	NEG
**Washington-35_sample-24**	POS	NEG	NEG	NEG	NEG	POS	NEG	NEG	POS	NEG	NEG
**Washington-36_sample-25**	POS	NEG	NEG	NEG	NEG	POS	NEG	NEG	POS	NEG	NEG
**Washington-25_sample-4007**	NEG	NEG	NEG	NEG	NEG	POS	NEG	NEG	POS	NEG	NEG
**Washington-28_sample-5008**	POS	NEG	NEG	NEG	NEG	POS	NEG	NEG	POS	NEG	NEG
**Washington-06_sample-556**	NEG	NEG	NEG	AMB	POS	POS	NEG	NEG	POS	NEG	POS
**Washington-15_sample-1007**	POS	NEG	NEG	AMB	POS	POS	NEG	NEG	POS	NEG	POS
**Washington-20_sample-2039**	NEG	NEG	NEG	POS	POS	POS	NEG	NEG	POS	NEG	POS

ISOLATE	RULENCE : HLG AND LEUKOCIDINS								VI		
	Panton Valentine leukocidin S	F component from hypothetical	S component from hypothetical	leukocidin D component	leukocidin E component	leukocidin/ haemolysin toxin family	leukocidin/haemolysin toxin family protein		putative membrane protein	haemolysin alpha	putative mem
	lukS-PV	**lukF-PV (P83)**	***lukM***	***lukD***	***lukE***	***lukX***	***lukY***	***lukY* (ST30+ST45)**	***hl***	***hla***	***hlIII* (cons)**
	median(lukS-PV_2	median(lukF-PV-P	median(lukM_11)	median(lukD_11)	median(lukE_11)	median(lukX_11)	median(lukY-var1_	median(lukY-var2_	median(hl_11)	median(hla_11)	median(hp_hlIII_6
**Washington-01_sample-106**	NEG	NEG	NEG	POS	POS	POS	AMB	NEG	POS	AMB	POS
**Washington-02_sample-115**	NEG	NEG	NEG	POS	POS	POS	NEG	NEG	POS	AMB	POS
**Washington-03_sample-117**	NEG	NEG	NEG	POS	POS	POS	POS	NEG	POS	POS	POS
**Washington-04_sample-120**	NEG	NEG	NEG	POS	POS	POS	AMB	NEG	POS	AMB	POS
**Washington-05_sample-505**	NEG	NEG	NEG	POS	POS	POS	NEG	NEG	POS	NEG	POS
**Washington-07_sample-611**	NEG	NEG	NEG	POS	POS	POS	NEG	NEG	POS	NEG	POS
**Washington-12_sample-807**	NEG	NEG	NEG	POS	POS	POS	NEG	NEG	POS	AMB	POS
**Washington-13_sample-811**	NEG	NEG	NEG	POS	POS	POS	NEG	NEG	POS	AMB	POS
**Washington-21_sample-2043**	POS	NEG	NEG	NEG	NEG	POS	NEG	NEG	POS	AMB	POS
**Washington-17_sample-2006**	NEG	NEG	NEG	POS	AMB	POS	AMB	NEG	POS	POS	POS
**Washington-30_sample-5025_Subclone 1**	NEG	NEG	NEG	POS	POS	POS	POS	NEG	POS	POS	POS

**From the previous study - for comparison:**											
**Washington-37_sample-48_ST22_Primate**	POS	NEG	NEG	NEG	NEG	POS	NEG	NEG	POS	NEG	NEG
**Washington-38_sample-06_ST22_Swine**	POS	NEG	NEG	NEG	NEG	POS	NEG	NEG	POS	NEG	NEG

ISOLATE	RULENCE : HAEMOLYSINS					VIRULENCE : HLB-CONV PHAGES			VIRULENCE : EXFOL.TOXINS		
	brane protein	haemolysin beta				staphylo-kinase	chemotaxis-inhibiting protein	Staphyl. Comple-ment	exfoliative toxin serotype A	exfoliative toxin serotype B	exfoliative toxin D
	hlIII (other than RF122)	hlb-probe 1	hlb-probe 2	hlb-probe 3	untruncated *hlb*	*sak*	*chp*	*scn*	*etA*	*etB*	*etD*
	median(hl-III_11)	median(hp_hlb_61	median(hp_hlb_612	median(hp_hlb_613	median(hlb_11;hlb	median(sak_11;hp_	median(hp_chp_61	median(hp_scn_61	median(8,2-etA)	median(9,3-etB)	median(etD_11)
Washington-08_sample-640	NEG	POS	POS	AMB	NEG	POS	POS	POS	NEG	NEG	NEG
Washington-09_sample-657	NEG	POS	POS	AMB	NEG	POS	POS	POS	NEG	NEG	NEG
Washington-10_sample-801	NEG	POS	POS	AMB	NEG	POS	POS	POS	NEG	NEG	NEG
Washington-11_sample-804	NEG	POS	POS	AMB	NEG	POS	POS	POS	NEG	NEG	NEG
Washington-14_sample-1005	NEG	POS	POS	POS	NEG	POS	POS	POS	NEG	NEG	NEG
Washington-16_sample-1027	NEG	POS	POS	AMB	POS	NEG	NEG	NEG	NEG	NEG	NEG
Washington-18_sample-2021	NEG	POS	POS	AMB	NEG	POS	POS	POS	NEG	NEG	NEG
Washington-19_sample-2027	NEG	POS	POS	POS	NEG	POS	POS	POS	NEG	NEG	NEG
Washington-22_sample-2051	NEG	POS	POS	POS	NEG	POS	POS	POS	NEG	NEG	NEG
Washington-23_sample-3009	NEG	POS	POS	POS	NEG	POS	POS	POS	NEG	NEG	NEG
Washington-24_sample-3022	NEG	POS	POS	AMB	NEG	POS	POS	POS	NEG	NEG	NEG
Washington-26_sample-4021	NEG	POS	POS	NEG	POS	NEG	NEG	NEG	NEG	NEG	NEG
Washington-27_sample-4033	NEG	POS	POS	POS	NEG	POS	POS	POS	NEG	NEG	NEG
Washington-29_sample-5018	NEG	POS	POS	AMB	NEG	POS	POS	POS	NEG	NEG	NEG
Washington-31_sample-5040	NEG	POS	POS	AMB	NEG	POS	POS	POS	NEG	NEG	NEG
Washington-30_sample 5025_Subclone 2	NEG	POS	POS	POS	NEG	POS	POS	POS	NEG	NEG	NEG
Washington-32_sample-21	NEG	POS	POS	AMB	NEG	POS	NEG	POS	NEG	NEG	NEG
Washington-33_sample-22	NEG	POS	POS	POS	NEG	POS	POS	POS	NEG	NEG	NEG
Washington-34_sample-23	NEG	POS	POS	AMB	NEG	POS	POS	POS	NEG	NEG	NEG
Washington-35_sample-24	NEG	POS	POS	NEG	NEG	POS	POS	NEG	NEG	NEG	NEG
Washington-36_sample-25	NEG	POS	POS	NEG	NEG	POS	POS	AMB	NEG	NEG	NEG
Washington-25_sample-4007	NEG	POS	POS	NEG	POS	NEG	NEG	NEG	NEG	NEG	NEG
Washington-28_sample-5008	NEG	POS	POS	AMB	NEG	POS	POS	POS	NEG	NEG	NEG
Washington-06_sample-556	POS	POS	POS	AMB	NEG	POS	POS	POS	NEG	NEG	NEG
Washington-15_sample-1007	POS	POS	POS	POS	NEG	POS	POS	POS	NEG	NEG	NEG
Washington-20_sample-2039	NEG	POS	POS	AMB	NEG	POS	NEG	POS	POS	NEG	NEG

ISOLATE	RULENCE : HAEMOLYSINS					VIRULENCE : HLB-CONV PHAGES			VIRULENCE : EXFOL.TOXINS		
	brane protein	haemolysin beta				staphylo-kinase	chemotaxis-inhibiting protein	Staphyl. Comple-ment	exfoliative toxin serotype A	exfoliative toxin serotype B	exfoliative toxin D
	hlIII **(other than RF122)**	**hlb-probe 1**	**hlb-probe 2**	**hlb-probe 3**	**untruncated *hlb***	***sak***	***chp***	***scn***	***etA***	***etB***	***etD***
	median(hl-III_11)	median(hp_hlb_61	median(hp_hlb_612	median(hp_hlb_61	median(hlb_11;hlb	median(sak_11;hp_	median(hp_chp_61	median(hp_scn_61	median(8,2-etA)	median(9,3-etB)	median(etD_11)
**Washington-01_sample-106**	**POS**	**NEG**	**POS**	**NEG**	**NEG**	**POS**	**NEG**	**POS**	**NEG**	**NEG**	**NEG**
**Washington-02_sample-115**	**POS**	**NEG**	**POS**	**NEG**	**NEG**	**POS**	**NEG**	**POS**	**NEG**	**NEG**	**NEG**
**Washington-03_sample-117**	**POS**	**NEG**	**POS**	**NEG**	**NEG**	**POS**	**NEG**	**POS**	**NEG**	**NEG**	**NEG**
**Washington-04_sample-120**	**POS**	**NEG**	**POS**	**NEG**	**NEG**	**POS**	**NEG**	**POS**	**NEG**	**NEG**	**NEG**
**Washington-05_sample-505**	**POS**	**NEG**	**POS**	**NEG**	**NEG**	**POS**	**NEG**	**POS**	**NEG**	**NEG**	**NEG**
**Washington-07_sample-611**	**POS**	**NEG**	**POS**	**NEG**	**NEG**	**POS**	**NEG**	**POS**	**NEG**	**NEG**	**NEG**
**Washington-12_sample-807**	**POS**	**NEG**	**POS**	**NEG**	**NEG**	**POS**	**NEG**	**POS**	**POS**	**NEG**	**NEG**
**Washington-13_sample-811**	**POS**	**NEG**	**POS**	**NEG**	**NEG**	**POS**	**NEG**	**POS**	**POS**	**NEG**	**NEG**
**Washington-21_sample-2043**	**POS**	**NEG**	**NEG**	**NEG**	**NEG**	**NEG**	**NEG**	**POS**	**NEG**	**NEG**	**NEG**
**Washington-17_sample-2006**	**POS**	**POS**	**POS**	**POS**	**NEG**	**POS**	**POS**	**POS**	**NEG**	**NEG**	**POS**
**Washington-30_sample-5025_Subclone 1**	**POS**	**POS**	**POS**	**POS**	**NEG**	**POS**	**POS**	**POS**	**NEG**	**NEG**	**POS**

From the previous study - for comparison:

**Washington-37_sample-48_ST22_Primate**	**NEG**	**POS**	**POS**	**NEG**	**NEG**	**POS**	**POS**	**AMB**	**NEG**	**NEG**	**NEG**
**Washington-38_sample-06_ST22_Swine**	**NEG**	**POS**	**POS**	**NEG**	**NEG**	**POS**	**POS**	**POS**	**NEG**	**NEG**	**NEG**

ISOLATE	VIRULENCE : EPITHEL. DIFF. INHIB			VIRULENCE : ACME LOCUS							
	epidermal cell differen-tiation	epidermal cell differen-tiation	epidermal cell differen-tiation	ACME-locus	ACME-locus	ACME: ornithincarb-amoyltrans-ferase	ACME:carbamat-kinase	ACME-locus: arginine/orni-	aureolysin		
	edinA	***edinB***	***edinC***	**ACME (total)**	**arcA-SCC**	**arcB-SCC**	**arcC-SCC**	**arcD-SCC**	***aur* (cons)**	***aur* (Not MRSA252)**	***aur* (MRSA252)**
	median(edinA_11)	median(edinB_11)	median(edinC_11)		median(hp_arcA_6	median(hp_arcB_6	median(hp_arcC_6	median(hp_arcD_6	median(hp_aur_61	median(hp_aur_61	median(hp_aur_612
**Washington-08_sample-640**	NEG	NEG	NEG	NEG	NEG	NEG	NEG	NEG	POS	NEG	POS
**Washington-09_sample-657**	NEG	NEG	NEG	NEG	NEG	NEG	NEG	NEG	POS	NEG	POS
**Washington-10_sample-801**	NEG	NEG	NEG	NEG	NEG	NEG	NEG	NEG	POS	NEG	POS
**Washington-11_sample-804**	NEG	NEG	NEG	NEG	NEG	NEG	NEG	NEG	POS	NEG	POS
**Washington-14_sample-1005**	NEG	NEG	NEG	NEG	NEG	NEG	NEG	NEG	POS	NEG	POS
**Washington-16_sample-1027**	NEG	NEG	NEG	NEG	NEG	NEG	NEG	NEG	POS	NEG	POS
**Washington-18_sample-2021**	NEG	NEG	NEG	NEG	NEG	NEG	NEG	NEG	POS	NEG	POS
**Washington-19_sample-2027**	NEG	NEG	NEG	NEG	NEG	NEG	NEG	NEG	POS	NEG	POS
**Washington-22_sample-2051**	NEG	NEG	NEG	NEG	NEG	NEG	NEG	NEG	POS	NEG	POS
**Washington-23_sample-3009**	NEG	NEG	NEG	NEG	NEG	NEG	NEG	NEG	POS	NEG	POS
**Washington-24_sample-3022**	NEG	NEG	NEG	NEG	NEG	NEG	NEG	NEG	POS	NEG	POS
**Washington-26_sample-4021**	NEG	NEG	NEG	NEG	NEG	NEG	NEG	NEG	POS	NEG	POS
**Washington-27_sample-4033**	NEG	NEG	NEG	NEG	NEG	NEG	NEG	NEG	POS	NEG	POS
**Washington-29_sample-5018**	NEG	NEG	NEG	NEG	NEG	NEG	NEG	NEG	POS	NEG	POS
**Washington-31_sample-5040**	NEG	NEG	NEG	NEG	NEG	NEG	NEG	NEG	POS	NEG	POS
**Washington-30_sample 5025_Subclone 2**	NEG	NEG	NEG	NEG	NEG	NEG	NEG	NEG	POS	NEG	POS
**Washington-32_sample-21**	NEG	NEG	NEG	NEG	NEG	NEG	NEG	NEG	POS	NEG	POS
**Washington-33_sample-22**	NEG	NEG	NEG	NEG	NEG	NEG	NEG	NEG	POS	NEG	POS
**Washington-34_sample-23**	NEG	NEG	NEG	NEG	NEG	NEG	NEG	NEG	POS	NEG	POS
**Washington-35_sample-24**	NEG	NEG	NEG	NEG	NEG	NEG	NEG	NEG	NEG	NEG	POS
**Washington-36_sample-25**	NEG	NEG	NEG	NEG	NEG	NEG	NEG	NEG	AMB	NEG	POS
**Washington-25_sample-4007**	NEG	NEG	NEG	NEG	NEG	NEG	NEG	NEG	POS	NEG	POS
**Washington-28_sample-5008**	NEG	NEG	NEG	NEG	NEG	NEG	NEG	NEG	POS	NEG	POS
**Washington-06_sample-556**	NEG	NEG	NEG	NEG	NEG	NEG	NEG	NEG	POS	POS	NEG
**Washington-15_sample-1007**	NEG	NEG	NEG	NEG	NEG	NEG	NEG	NEG	POS	POS	NEG
**Washington-20_sample-2039**	NEG	NEG	NEG	NEG	NEG	NEG	NEG	NEG	POS	POS	NEG

ISOLATE	VIRULENCE : EPITHEL. DIFF. INHIB			VIRULENCE : ACME LOCUS							
	epidermal cell differen-tiation	epidermal cell differen-tiation	epidermal cell differen-tiation	ACME-locus	ACME-locus	ACME: ornithincarb-amoyltrans-ferase	ACME:carba mat-kinase	ACME-locus: arginine/orni-	aureolysin		
	ednA	***ednB***	***ednC***	**ACME (total)**	**arcA-SCC**	**arcB-SCC**	**arcC-SCC**	**arcD-SCC**	***aur* (cons)**	***aur* (Not MRSA252)**	***aur* (MRSA252)**
	median(edinA_11)	median(edinB_11)	median(edinC_11)		median(hp_arcA_6	median(hp_arcB_6	median(hp_arcC_6	median(hp_arcD_6	median(hp_aur_61	median(hp_aur_61	median(hp_aur_61
**Washington-01_sample-106**	NEG	NEG	NEG	NEG	NEG	NEG	NEG	NEG	POS	POS	NEG
**Washington-02_sample-115**	NEG	NEG	NEG	NEG	NEG	NEG	NEG	NEG	POS	POS	NEG
**Washington-03_sample-117**	NEG	NEG	NEG	NEG	NEG	NEG	NEG	NEG	POS	POS	NEG
**Washington-04_sample-120**	NEG	NEG	NEG	NEG	NEG	NEG	NEG	NEG	POS	POS	NEG
**Washington-05_sample-505**	NEG	NEG	NEG	NEG	NEG	NEG	NEG	NEG	POS	POS	NEG
**Washington-07_sample-611**	NEG	NEG	NEG	NEG	NEG	NEG	NEG	NEG	POS	POS	NEG
**Washington-12_sample-807**	NEG	NEG	NEG	NEG	NEG	NEG	NEG	NEG	POS	POS	NEG
**Washington-13_sample-811**	NEG	NEG	NEG	NEG	NEG	NEG	NEG	NEG	POS	POS	NEG
**Washington-21_sample-2043**	NEG	NEG	NEG	NEG	NEG	NEG	NEG	NEG	POS	POS	NEG
**Washington-17_sample-2006**	NEG	POS	NEG	NEG	NEG	NEG	NEG	NEG	POS	POS	NEG
**Washington-30_sample-5025_Subclone 1**	NEG	POS	NEG	NEG	NEG	NEG	NEG	NEG	POS	POS	NEG
**From the previous study - for comparison:**											
**Washington-37_sample-48_ST22_Primate**	NEG	NEG	NEG	NEG	NEG	NEG	NEG	NEG	AMB	NEG	POS
**Washington-38_sample-06_ST22_Swine**	NEG	NEG	NEG	NEG	NEG	NEG	NEG	NEG	POS	NEG	POS

ISOLATE	VIRULENCE : PROTEASES										
	serin-protease A	serin-protease B	serin-protease E	glutamylend opeptidase	staphopain B, protease	staphopain A (staphylopain A), protease		staphyl. exotoxin-like protein			
	splA	*splB*	*splE*	*sspA*	*sspB*	*sspP* (cons)	*sspP* (other than ST93)	*setC*	set6-var1_11	set6-var2_11	set6-var1_12
	median(splA_11)	median(splB_11)	median(hp_splE_6	median(hp_sspA_6	median(hp_sspB_6	median(hp_sspP_6	median(hp_sspP_6	median(setC-MW0	median(set6-var1_1	median(set6-var2_1	median(set6-var1_
Washington-08_sample-640	NEG	NEG	NEG	POS	POS	POS	POS	POS	NEG	POS	AMB
Washington-09_sample-657	NEG	NEG	NEG	POS	POS	POS	POS	POS	NEG	POS	AMB
Washington-10_sample-801	NEG	NEG	NEG	POS	POS	POS	POS	POS	NEG	POS	AMB
Washington-11_sample-804	NEG	NEG	NEG	POS	POS	POS	POS	POS	NEG	POS	NEG
Washington-14_sample-1005	NEG	NEG	NEG	POS	POS	POS	POS	POS	NEG	POS	AMB
Washington-16_sample-1027	NEG	NEG	NEG	POS	POS	POS	POS	POS	NEG	POS	POS
Washington-18_sample-2021	NEG	NEG	NEG	POS	POS	POS	POS	POS	NEG	POS	AMB
Washington-19_sample-2027	NEG	NEG	NEG	POS	POS	POS	POS	POS	NEG	POS	POS
Washington-22_sample-2051	NEG	NEG	NEG	POS	POS	POS	POS	POS	NEG	POS	POS
Washington-23_sample-3009	NEG	NEG	NEG	POS	POS	POS	POS	POS	NEG	POS	POS
Washington-24_sample-3022	NEG	NEG	NEG	POS	POS	POS	POS	POS	NEG	POS	AMB
Washington-26_sample-4021	NEG	NEG	NEG	POS	POS	POS	POS	POS	NEG	POS	AMB
Washington-27_sample-4033	NEG	NEG	NEG	POS	POS	POS	POS	POS	NEG	POS	POS
Washington-29_sample-5018	NEG	NEG	NEG	POS	POS	POS	POS	POS	NEG	POS	AMB
Washington-31_sample-5040	NEG	NEG	NEG	POS	POS	POS	POS	POS	NEG	POS	AMB
Washington-30_sample 5025_Subclone 2	NEG	NEG	NEG	POS	POS	POS	POS	POS	NEG	POS	POS
Washington-32_sample-21	NEG	NEG	NEG	POS	POS	POS	POS	POS	NEG	POS	AMB
Washington-33_sample-22	NEG	NEG	NEG	POS	POS	POS	POS	POS	NEG	POS	POS
Washington-34_sample-23	NEG	NEG	NEG	POS	POS	POS	POS	POS	NEG	POS	AMB
Washington-35_sample-24	NEG	NEG	NEG	POS	POS	POS	POS	POS	NEG	POS	NEG
Washington-36_sample-25	NEG	NEG	NEG	POS	POS	POS	POS	POS	NEG	POS	NEG
Washington-25_sample-4007	NEG	NEG	NEG	POS	POS	POS	POS	POS	NEG	POS	AMB
Washington-28_sample-5008	NEG	NEG	NEG	POS	POS	POS	POS	POS	NEG	POS	AMB
Washington-06_sample-556	POS	POS	NEG	POS	POS	POS	POS	POS	NEG	NEG	NEG
Washington-15_sample-1007	POS	POS	NEG	POS	POS	POS	POS	POS	NEG	NEG	NEG
Washington-20_sample-2039	POS	POS	NEG	POS	POS	POS	POS	POS	POS	NEG	POS

ISOLATE	VIRULENCE : PROTEASES										
	serin-protease A	serin-protease B	serin-protease E	glutamylendopeptidase	staphopain B, protease	staphopain A (staphylopain A), protease		staphyl. exotoxin-like protein			
	splA	***splB***	***splE***	***sspA***	***sspB***	***sspP* (cons)**	***sspP* (other than ST93)**	***setC***	**set6-var1_11**	**set6-var2_11**	**set6-var1_12**
	median(splA_11)	median(splB_11)	median(hp_splE_6	median(hp_sspA_6	median(hp_sspB_6	median(hp_sspP_6	median(hp_sspP_6	median(setC-MW0	median(set6-var1_1	median(set6-var2_1	median(set6-var1_1
**Washington-01_sample-106**	**NEG**	**NEG**	**NEG**	**POS**	**POS**	**POS**	**POS**	**POS**	**AMB**	**POS**	**NEG**
**Washington-02_sample-115**	**NEG**	**NEG**	**NEG**	**POS**	**POS**	**POS**	**POS**	**POS**	**NEG**	**POS**	**NEG**
**Washington-03_sample-117**	**NEG**	**NEG**	**NEG**	**POS**	**POS**	**POS**	**POS**	**POS**	**POS**	**POS**	**AMB**
**Washington-04_sample-120**	**NEG**	**NEG**	**NEG**	**POS**	**POS**	**POS**	**POS**	**POS**	**NEG**	**POS**	**NEG**
**Washington-05_sample-505**	**NEG**	**NEG**	**NEG**	**POS**	**POS**	**POS**	**POS**	**POS**	**NEG**	**POS**	**NEG**
**Washington-07_sample-611**	**NEG**	**NEG**	**NEG**	**POS**	**POS**	**POS**	**POS**	**POS**	**NEG**	**POS**	**NEG**
**Washington-12_sample-807**	**NEG**	**NEG**	**NEG**	**POS**	**POS**	**POS**	**POS**	**POS**	**NEG**	**POS**	**NEG**
**Washington-13_sample-811**	**NEG**	**NEG**	**NEG**	**POS**	**POS**	**POS**	**POS**	**POS**	**AMB**	**POS**	**NEG**
**Washington-21_sample-2043**	**NEG**	**NEG**	**NEG**	**POS**	**POS**	**POS**	**POS**	**POS**	**POS**	**NEG**	**NEG**
**Washington-17_sample-2006**	**NEG**	**POS**	**POS**	**POS**	**POS**	**POS**	**POS**	**POS**	**AMB**	**POS**	**NEG**
**Washington-30_sample-5025_Subclone 1**	**POS**	**POS**	**POS**	**POS**	**POS**	**POS**	**POS**	**POS**	**NEG**	**POS**	**NEG**
**From the previous study - for comparison:**											
**Washington-37_sample-48_ST22_Primate**	**NEG**	**NEG**	**NEG**	**POS**	**POS**	**POS**	**POS**	**POS**	**NEG**	**POS**	**NEG**
**Washington-38_sample-06_ST22_Swine**	**NEG**	**NEG**	**NEG**	**POS**	**POS**	**POS**	**POS**	**POS**	**NEG**	**POS**	**AMB**

ISOLATE	staphylococcal superantigen-like protein 1									staphylococcal superantigen-like protein 2	
	set6-var2_12	set6-var4_11	ssl01-RF122	ssl01/set6 (COL)	ssl01/set6 (Mu50+N315)	ssl01/set6 (MW2+MSSA4	ssl01/set6 (MRSA252)	ssl01/set6 (RF122)	ssl01/set6 (other alleles)	ssl02/set7	ssl02/set7 (MRSA252)
	median(set6-var2_1	median(set6-var4_1	median(hp_ssl01_6	WENN(UND(	WENN(UND(set6-	WENN((UND(set6	WENN((UND	WENN((UND	WENN(UND	WENN((set7-	WENN((set7-
Washington-08_sample-640	NEG	NEG	NEG	NEG	NEG	NEG	NEG	NEG	POS	NEG	POS
Washington-09_sample-657	NEG	NEG	NEG	NEG	NEG	NEG	NEG	NEG	POS	NEG	POS
Washington-10_sample-801	NEG	NEG	NEG	NEG	NEG	NEG	NEG	NEG	POS	NEG	POS
Washington-11_sample-804	NEG	NEG	NEG	NEG	NEG	NEG	NEG	NEG	POS	NEG	NEG
Washington-14_sample-1005	NEG	NEG	NEG	NEG	NEG	NEG	NEG	NEG	POS	NEG	NEG
Washington-16_sample-1027	NEG	NEG	NEG	NEG	NEG	NEG	POS	NEG	NEG	NEG	POS
Washington-18_sample-2021	NEG	NEG	NEG	NEG	NEG	NEG	NEG	NEG	POS	NEG	NEG
Washington-19_sample-2027	NEG	NEG	NEG	NEG	NEG	NEG	POS	NEG	NEG	AMB	POS
Washington-22_sample-2051	NEG	NEG	NEG	NEG	NEG	NEG	POS	NEG	NEG	AMB	POS
Washington-23_sample-3009	NEG	NEG	NEG	NEG	NEG	NEG	POS	NEG	NEG	AMB	POS
Washington-24_sample-3022	NEG	NEG	NEG	NEG	NEG	NEG	NEG	NEG	POS	NEG	NEG
Washington-26_sample-4021	NEG	NEG	NEG	NEG	NEG	NEG	NEG	NEG	POS	NEG	NEG
Washington-27_sample-4033	NEG	NEG	NEG	NEG	NEG	NEG	POS	NEG	NEG	NEG	POS
Washington-29_sample-5018	NEG	NEG	NEG	NEG	NEG	NEG	NEG	NEG	POS	NEG	NEG
Washington-31_sample-5040	NEG	NEG	NEG	NEG	NEG	NEG	NEG	NEG	POS	NEG	POS
Washington-30_sample 5025_Subclone 2	NEG	NEG	NEG	NEG	NEG	NEG	POS	NEG	NEG	AMB	POS
Washington-32_sample-21	NEG	NEG	NEG	NEG	NEG	NEG	NEG	NEG	POS	NEG	NEG
Washington-33_sample-22	NEG	NEG	NEG	NEG	NEG	NEG	POS	NEG	NEG	NEG	POS
Washington-34_sample-23	NEG	NEG	NEG	NEG	NEG	NEG	NEG	NEG	POS	NEG	NEG
Washington-35_sample-24	NEG	NEG	NEG	NEG	NEG	NEG	NEG	NEG	POS	NEG	NEG
Washington-36_sample-25	NEG	NEG	NEG	NEG	NEG	NEG	NEG	NEG	POS	NEG	NEG
Washington-25_sample-4007	NEG	NEG	NEG	NEG	NEG	NEG	NEG	NEG	POS	NEG	NEG
Washington-28_sample-5008	NEG	NEG	NEG	NEG	NEG	NEG	NEG	NEG	POS	NEG	NEG
Washington-06_sample-556	NEG	POS	NEG	NEG	NEG	NEG	NEG	NEG	POS	NEG	NEG
Washington-15_sample-1007	NEG	POS	NEG	NEG	NEG	NEG	NEG	NEG	POS	POS	NEG
Washington-20_sample-2039	NEG	NEG	NEG	NEG	POS	NEG	NEG	NEG	NEG	NEG	NEG

ISOLATE	staphylococcal superantigen-like protein 1									staphylococcal superantigen-like protein 2	
	set6-var2_12	set6-var4_11	ssl01-RF122	ssl01/set6 (COL)	ssl01/set6 (Mu50+N315)	ssl01/set6 (MW2+MSSA4	ssl01/set6 (MRSA252)	ssl01/set6 (RF122)	ssl01/set6 (other alleles)	ssl02/set7	ssl02/set7 (MRSA252)
	median(set6-var2_1	median(set6-var4_1	median(hp_ssl01_6	WENN(UND(	WENN(UND(set6-	WENN((UND(set6	WENN((UND	WENN((UND	WENN(UND	WENN((set7-	WENN((set7-
**Washington-01_sample-106**	**POS**	**NEG**	**NEG**	**NEG**	**NEG**	**POS**	**NEG**	**NEG**	**NEG**	**NEG**	**NEG**
**Washington-02_sample-115**	**POS**	**NEG**	**NEG**	**NEG**	**NEG**	**POS**	**NEG**	**NEG**	**NEG**	**NEG**	**NEG**
**Washington-03_sample-117**	**POS**	**NEG**	**NEG**	**NEG**	**NEG**	**POS**	**NEG**	**NEG**	**NEG**	**POS**	**NEG**
**Washington-04_sample-120**	**POS**	**NEG**	**NEG**	**NEG**	**NEG**	**POS**	**NEG**	**NEG**	**NEG**	**NEG**	**NEG**
**Washington-05_sample-505**	**POS**	**NEG**	**NEG**	**NEG**	**NEG**	**POS**	**NEG**	**NEG**	**NEG**	**NEG**	**NEG**
**Washington-07_sample-611**	**POS**	**NEG**	**NEG**	**NEG**	**NEG**	**POS**	**NEG**	**NEG**	**NEG**	**NEG**	**NEG**
**Washington-12_sample-807**	**POS**	**NEG**	**NEG**	**NEG**	**NEG**	**POS**	**NEG**	**NEG**	**NEG**	**NEG**	**NEG**
**Washington-13_sample-811**	**POS**	**NEG**	**NEG**	**NEG**	**NEG**	**POS**	**NEG**	**NEG**	**NEG**	**NEG**	**NEG**
**Washington-21_sample-2043**	**NEG**	**POS**	**NEG**	**POS**	**AMB**	**NEG**	**NEG**	**NEG**	**NEG**	**POS**	**NEG**
**Washington-17_sample-2006**	**NEG**	**NEG**	**NEG**	**NEG**	**NEG**	**NEG**	**NEG**	**NEG**	**POS**	**POS**	**NEG**
**Washington-30_sample-5025_Subclone 1**	**NEG**	**NEG**	**NEG**	**NEG**	**NEG**	**NEG**	**NEG**	**NEG**	**POS**	**POS**	**AMB**
**From the previous study - for comparison:**											
**Washington-37_sample-48_ST22_Primate**	**NEG**	**NEG**	**NEG**	**NEG**	**NEG**	**NEG**	**NEG**	**NEG**	**POS**	**NEG**	**NEG**
**Washington-38_sample-06_ST22_Swine**	**NEG**	**NEG**	**NEG**	**NEG**	**NEG**	**NEG**	**NEG**	**NEG**	**POS**	**NEG**	**NEG**

ISOLATE	VIRULENCE : STAPHYLOCOCCAL SUPERANTIGEN/ENTEROTOXIN-LIKE GENES										
	staphylococcal superantigen-like protein 3			staphylococcal superantigen-like protein 4		staphylococcal superantigen-like protein 5				staphylococcal superantigen-like protein 6	
	ssl03/set8_p robe 1	ssl03/set8_p robe 2	ssl03/set8 (MRSA252	ssl04/set9	ssl04/set9 (MRSA252	ssl05/set3_prob e 1	ssl05/set3 (RF122, probe-	ssl05/set3_prob e 2 (612)	ssl05/set3 (MRSA252)	ssl06/set21	ssl06 (NCTC8325+M
	median(set8)	median(hp_ssl03_6	median(SAR0424)	median(set9-var1_1	median(set-SAR04	set3-var1_11	WENN((hp_s	WENN((hp_s	median(set3-var2)	median(set21_11)	median(hp_ssl06_6
**Washington-08_sample-640**	NEG	NEG	NEG	NEG	POS	AMB	NEG	NEG	POS	NEG	NEG
**Washington-09_sample-657**	NEG	NEG	NEG	NEG	AMB	AMB	NEG	NEG	POS	NEG	NEG
**Washington-10_sample-801**	NEG	NEG	NEG	NEG	AMB	AMB	NEG	NEG	POS	NEG	NEG
**Washington-11_sample-804**	NEG	NEG	NEG	NEG	AMB	NEG	NEG	NEG	POS	NEG	NEG
**Washington-14_sample-1005**	NEG	NEG	NEG	NEG	POS	AMB	NEG	NEG	POS	NEG	NEG
**Washington-16_sample-1027**	NEG	NEG	NEG	NEG	POS	POS	NEG	NEG	POS	NEG	NEG
**Washington-18_sample-2021**	NEG	NEG	NEG	NEG	AMB	NEG	NEG	NEG	POS	NEG	NEG
**Washington-19_sample-2027**	NEG	NEG	POS	NEG	POS	POS	NEG	NEG	POS	NEG	NEG
**Washington-22_sample-2051**	NEG	NEG	POS	NEG	POS	POS	NEG	NEG	POS	NEG	NEG
**Washington-23_sample-3009**	NEG	NEG	NEG	NEG	POS	POS	NEG	NEG	POS	NEG	NEG
**Washington-24_sample-3022**	NEG	NEG	NEG	NEG	POS	NEG	NEG	NEG	POS	NEG	NEG
**Washington-26_sample-4021**	NEG	NEG	NEG	NEG	AMB	NEG	NEG	NEG	POS	NEG	NEG
**Washington-27_sample-4033**	NEG	NEG	NEG	NEG	POS	AMB	NEG	NEG	POS	NEG	NEG
**Washington-29_sample-5018**	NEG	NEG	NEG	NEG	POS	NEG	NEG	NEG	POS	NEG	NEG
**Washington-31_sample-5040**	NEG	NEG	AMB	NEG	AMB	NEG	NEG	NEG	POS	NEG	NEG
**Washington-30_sample 5025_Subclone 2**	NEG	NEG	NEG	NEG	POS	POS	NEG	NEG	POS	NEG	NEG
**Washington-32_sample-21**	NEG	NEG	NEG	NEG	POS	NEG	NEG	NEG	POS	NEG	NEG
**Washington-33_sample-22**	NEG	NEG	NEG	NEG	POS	AMB	NEG	NEG	POS	NEG	NEG
**Washington-34_sample-23**	NEG	NEG	NEG	NEG	POS	NEG	NEG	NEG	POS	NEG	NEG
**Washington-35_sample-24**	NEG	NEG	NEG	NEG	AMB	NEG	NEG	NEG	POS	NEG	NEG
**Washington-36_sample-25**	NEG	NEG	NEG	NEG	AMB	NEG	NEG	NEG	POS	NEG	NEG
**Washington-25_sample-4007**	NEG	NEG	NEG	NEG	POS	NEG	NEG	NEG	POS	NEG	NEG
**Washington-28_sample-5008**	NEG	NEG	NEG	NEG	POS	NEG	NEG	NEG	POS	NEG	NEG
**Washington-06_sample-556**	NEG	NEG	NEG	NEG	NEG	NEG	NEG	POS	NEG	POS	POS
**Washington-15_sample-1007**	POS	POS	NEG	POS	NEG	NEG	NEG	POS	NEG	POS	POS
**Washington-20_sample-2039**	POS	POS	NEG	POS	NEG	NEG	POS	NEG	NEG	NEG	POS

ISOLATE	VIRULENCE : STAPHYLOCOCCAL SUPERANTIGEN/ENTEROTOXIN-LIKE GENES										
	staphylococcal superantigen-like protein 3			staphylococcal superantigen-like protein 4		staphylococcal superantigen-like protein 5				staphylococcal superantigen-like protein 6	
	ssl03/set8_p robe 1	ssl03/set8_p robe 2	ssl03/set8 (MRSA252	ssl04/set9	ssl04/set9 (MRSA252	ssl05/set3_prob e 1	ssl05/set3 (RF122, probe-	ssl05/set3_prob e 2 (612)	ssl05/set3 (MRSA252)	ssl06/set21	ssl06 (NCTC8325+M
	median(set8)	median(hp_ssl03_6	median(SAR0424)	median(set9-var1_1	median(set-SAR04	set3-var1_11	WENN((hp_s	WENN((hp_s	median(set3-var2)	median(set21_11)	median(hp_ssl06_6
**Washington-01_sample-106**	POS	POS	NEG	POS	NEG	POS	POS	NEG	NEG	NEG	NEG
**Washington-02_sample-115**	POS	POS	NEG	POS	NEG	POS	POS	NEG	NEG	NEG	NEG
**Washington-03_sample-117**	POS	POS	NEG	POS	NEG	POS	POS	AMB	NEG	NEG	NEG
**Washington-04_sample-120**	POS	POS	NEG	POS	NEG	POS	POS	NEG	NEG	NEG	NEG
**Washington-05_sample-505**	POS	POS	NEG	POS	NEG	POS	POS	NEG	NEG	NEG	NEG
**Washington-07_sample-611**	POS	POS	NEG	POS	NEG	POS	POS	NEG	NEG	NEG	NEG
**Washington-12_sample-807**	POS	POS	NEG	POS	NEG	POS	POS	NEG	NEG	NEG	NEG
**Washington-13_sample-811**	POS	POS	NEG	POS	NEG	POS	POS	NEG	NEG	NEG	NEG
**Washington-21_sample-2043**	POS	POS	NEG	POS	NEG	POS	POS	NEG	NEG	NEG	NEG
**Washington-17_sample-2006**	POS	POS	NEG	POS	NEG	POS	NEG	POS	NEG	NEG	NEG
**Washington-30_sample-5025_Subclone 1**	POS	POS	NEG	POS	NEG	POS	AMB	POS	NEG	NEG	AMB

From the previous study - for comparison:

**Washington-37_sample-48_ST22_Primate**	NEG	NEG	NEG	NEG	AMB	NEG	NEG	NEG	POS	NEG	NEG
**Washington-38_sample-06_ST22_Swine**	NEG	NEG	NEG	NEG	POS	NEG	NEG	NEG	POS	NEG	NEG

ISOLATE	(SET/SSL)										
	staphylococcal superantigen-like protein 7			staphylococcal superantigen-like protein 8		staphylococcal superantigen-like protein 9			staphylococcal superantigen-like protein 10		
	ssl07/set1	ssl07/set1 (MRSA252)	ssl07/set1 (AF188836)	ssl08/set12_ probe 1	ssl08/set12_ probe 2	ssl09/set5_p robe 1	ssl09/set5_p robe 2	ssl09/set5 (MRSA252)	ssl10/set4	ssl10 (RF122)	ssl10/set4 (MRSA252)
	WENN((set1-	WENN((set1-	WENN((set1-	median(set12)	median(hp_ssl08_6	median(set5-var1)	median(hp_ssl09_6	median(set5-var2)	WENN((set4-	WENN((hp_s	WENN((set4-
Washington-08_sample-640	NEG	NEG	POS	NEG	NEG	AMB	POS	NEG	NEG	NEG	NEG
Washington-09_sample-657	NEG	NEG	POS	NEG	NEG	AMB	POS	NEG	NEG	NEG	NEG
Washington-10_sample-801	NEG	NEG	POS	NEG	NEG	NEG	POS	NEG	NEG	NEG	NEG
Washington-11_sample-804	NEG	NEG	POS	NEG	NEG	NEG	POS	NEG	NEG	NEG	NEG
Washington-14_sample-1005	NEG	NEG	POS	NEG	NEG	AMB	POS	NEG	NEG	NEG	NEG
Washington-16_sample-1027	NEG	NEG	POS	NEG	NEG	POS	POS	NEG	NEG	NEG	NEG
Washington-18_sample-2021	NEG	NEG	POS	NEG	NEG	AMB	POS	NEG	NEG	NEG	NEG
Washington-19_sample-2027	NEG	NEG	POS	NEG	NEG	POS	POS	NEG	NEG	NEG	NEG
Washington-22_sample-2051	NEG	NEG	POS	NEG	NEG	POS	POS	NEG	NEG	NEG	NEG
Washington-23_sample-3009	NEG	NEG	POS	NEG	NEG	POS	POS	NEG	NEG	NEG	NEG
Washington-24_sample-3022	NEG	NEG	POS	NEG	NEG	AMB	POS	NEG	NEG	NEG	NEG
Washington-26_sample-4021	NEG	NEG	POS	NEG	NEG	POS	POS	NEG	NEG	NEG	NEG
Washington-27_sample-4033	NEG	NEG	POS	NEG	NEG	POS	POS	NEG	NEG	NEG	NEG
Washington-29_sample-5018	NEG	NEG	POS	NEG	NEG	POS	POS	NEG	NEG	NEG	NEG
Washington-31_sample-5040	NEG	NEG	POS	NEG	NEG	POS	POS	NEG	NEG	NEG	NEG
Washington-30_sample 5025_Subclone 2	AMB	AMB	POS	NEG	NEG	POS	POS	NEG	POS	NEG	AMB
Washington-32_sample-21	NEG	NEG	POS	NEG	NEG	AMB	POS	NEG	NEG	NEG	NEG
Washington-33_sample-22	NEG	NEG	POS	NEG	NEG	POS	POS	NEG	NEG	NEG	NEG
Washington-34_sample-23	NEG	NEG	POS	NEG	NEG	POS	POS	NEG	NEG	NEG	NEG
Washington-35_sample-24	NEG	NEG	POS	NEG	NEG	NEG	POS	NEG	NEG	NEG	NEG
Washington-36_sample-25	NEG	NEG	POS	NEG	NEG	NEG	POS	NEG	NEG	NEG	NEG
Washington-25_sample-4007	NEG	NEG	POS	NEG	NEG	AMB	POS	NEG	NEG	NEG	NEG
Washington-28_sample-5008	NEG	NEG	POS	NEG	NEG	AMB	POS	NEG	NEG	NEG	NEG
Washington-06_sample-556	POS	NEG	NEG	POS	POS	POS	POS	NEG	POS	NEG	NEG
Washington-15_sample-1007	POS	NEG	NEG	POS	POS	POS	POS	NEG	POS	NEG	NEG
Washington-20_sample-2039	NEG	NEG	NEG	NEG	POS	POS	POS	NEG	NEG	POS	NEG

ISOLATE	(SET/SSL)										
	staphylococcal superantigen-like protein 7			staphylococcal superantigen-like protein 8		staphylococcal superantigen-like protein 9			staphylococcal superantigen-like protein 10		
	ssl07/set1	ssl07/set1 (MRSA252)	ssl07/set1 (AF188836)	ssl08/set12_ probe 1	ssl08/set12_ probe 2	ssl09/set5_p robe 1	ssl09/set5_p robe 2	ssl09/set5 (MRSA252)	ssl10/set4	ssl10 (RF122)	ssl10/set4 (MRSA252)
	WENN((set1-	WENN((set1-	WENN((set1-	median(set12)	median(hp_ssl08_6	median(set5-var1)	median(hp_ssl09_6	median(set5-var2)	WENN((set4-	WENN((hp_s	WENN((set4-
**Washington-01_sample-106**	**POS**	**NEG**	**NEG**	**POS**	**POS**	**POS**	**POS**	**NEG**	**POS**	**NEG**	**NEG**
**Washington-02_sample-115**	**POS**	**NEG**	**NEG**	**POS**	**POS**	**POS**	**POS**	**NEG**	**POS**	**NEG**	**NEG**
**Washington-03_sample-117**	**POS**	**NEG**	**NEG**	**POS**	**POS**	**POS**	**POS**	**NEG**	**POS**	**NEG**	**NEG**
**Washington-04_sample-120**	**POS**	**NEG**	**NEG**	**POS**	**POS**	**POS**	**POS**	**NEG**	**POS**	**NEG**	**NEG**
**Washington-05_sample-505**	**POS**	**NEG**	**NEG**	**POS**	**POS**	**POS**	**POS**	**NEG**	**POS**	**NEG**	**NEG**
**Washington-07_sample-611**	**POS**	**NEG**	**NEG**	**POS**	**POS**	**POS**	**POS**	**NEG**	**POS**	**NEG**	**NEG**
**Washington-12_sample-807**	**POS**	**NEG**	**NEG**	**POS**	**POS**	**POS**	**POS**	**NEG**	**POS**	**NEG**	**NEG**
**Washington-13_sample-811**	**POS**	**NEG**	**NEG**	**POS**	**POS**	**POS**	**POS**	**NEG**	**POS**	**NEG**	**NEG**
**Washington-21_sample-2043**	**NEG**	**NEG**	**NEG**	**POS**	**POS**	**POS**	**POS**	**NEG**	**POS**	**NEG**	**NEG**
**Washington-17_sample-2006**	**POS**	**NEG**	**NEG**	**POS**	**POS**	**POS**	**POS**	**NEG**	**POS**	**NEG**	**NEG**
**Washington-30_sample-5025_Subclone 1**	**POS**	**AMB**	**AMB**	**POS**	**POS**	**POS**	**POS**	**NEG**	**POS**	**AMB**	**AMB**
**From the previous study - for comparison:**											
**Washington-37_sample-48_ST22_Primate**	**NEG**	**NEG**	**POS**	**NEG**	**NEG**	**NEG**	**POS**	**NEG**	**NEG**	**NEG**	**NEG**
**Washington-38_sample-06_ST22_Swine**	**NEG**	**NEG**	**POS**	**NEG**	**NEG**	**POS**	**POS**	**NEG**	**NEG**	**NEG**	**NEG**

ISOLATE	staphylococcal superantigene-like protein 11				staphylococcal exotoxin-like protein, second locus					Capsule type 1	capsular poly-saccharide synthesis
	**ssl11/set2 (COL)**	**ssl11+set2(Mu50+N315)**	**ssl11+set2(MW2+MSSA47**	**ssl11/set2 (MRSA252)**	**setB3**	**setB3 (MRSA252)**	**setB2**	**setB2 (MRSA252)**	**setB1**	***cap1***	***capH1***
	median(set2-var4)	median(set2-var3)	median(set2-var1)	median(set2-var2)	median(setB-SA11	median(setB-SAR1	median(setB-SA11	median(setB-SAR1	median(setB-SA11	median(hp_capH1_	median(hp_capH1_
**Washington-08_sample-640**	NEG	NEG	NEG	NEG	NEG	NEG	NEG	NEG	NEG	NEG	NEG
**Washington-09_sample-657**	NEG	NEG	NEG	NEG	NEG	NEG	NEG	NEG	NEG	NEG	NEG
**Washington-10_sample-801**	NEG	NEG	NEG	NEG	NEG	NEG	NEG	NEG	NEG	NEG	NEG
**Washington-11_sample-804**	NEG	NEG	NEG	NEG	NEG	NEG	NEG	NEG	NEG	NEG	NEG
**Washington-14_sample-1005**	NEG	NEG	NEG	NEG	NEG	NEG	NEG	NEG	NEG	NEG	NEG
**Washington-16_sample-1027**	NEG	NEG	NEG	NEG	NEG	NEG	NEG	NEG	NEG	NEG	NEG
**Washington-18_sample-2021**	NEG	NEG	NEG	NEG	NEG	NEG	NEG	NEG	NEG	NEG	NEG
**Washington-19_sample-2027**	NEG	NEG	NEG	NEG	NEG	NEG	NEG	NEG	NEG	NEG	NEG
**Washington-22_sample-2051**	NEG	NEG	NEG	NEG	NEG	NEG	NEG	NEG	NEG	NEG	NEG
**Washington-23_sample-3009**	NEG	NEG	NEG	NEG	NEG	NEG	NEG	NEG	NEG	NEG	NEG
**Washington-24_sample-3022**	NEG	NEG	NEG	NEG	NEG	NEG	NEG	NEG	NEG	NEG	NEG
**Washington-26_sample-4021**	NEG	NEG	NEG	NEG	NEG	NEG	NEG	NEG	NEG	NEG	NEG
**Washington-27_sample-4033**	NEG	NEG	NEG	NEG	NEG	NEG	NEG	NEG	NEG	NEG	NEG
**Washington-29_sample-5018**	NEG	NEG	NEG	NEG	NEG	NEG	NEG	NEG	NEG	NEG	NEG
**Washington-31_sample-5040**	NEG	NEG	NEG	NEG	NEG	NEG	NEG	NEG	NEG	NEG	NEG
**Washington-30_sample 5025_Subclone 2**	NEG	NEG	NEG	NEG	NEG	NEG	NEG	NEG	NEG	NEG	NEG
**Washington-32_sample-21**	NEG	NEG	NEG	NEG	NEG	NEG	NEG	NEG	NEG	NEG	NEG
**Washington-33_sample-22**	NEG	NEG	NEG	NEG	NEG	NEG	NEG	NEG	NEG	NEG	NEG
**Washington-34_sample-23**	NEG	NEG	NEG	NEG	NEG	NEG	NEG	NEG	NEG	NEG	NEG
**Washington-35_sample-24**	NEG	NEG	NEG	NEG	NEG	NEG	NEG	NEG	NEG	NEG	NEG
**Washington-36_sample-25**	NEG	NEG	NEG	NEG	NEG	NEG	NEG	NEG	NEG	NEG	NEG
**Washington-25_sample-4007**	NEG	NEG	NEG	NEG	NEG	NEG	NEG	NEG	NEG	NEG	NEG
**Washington-28_sample-5008**	NEG	NEG	NEG	NEG	NEG	NEG	NEG	NEG	NEG	NEG	NEG
**Washington-06_sample-556**	NEG	NEG	NEG	NEG	POS	NEG	POS	NEG	POS	NEG	NEG
**Washington-15_sample-1007**	NEG	NEG	NEG	NEG	POS	NEG	POS	NEG	POS	NEG	NEG
**Washington-20_sample-2039**	NEG	NEG	NEG	NEG	POS	NEG	POS	NEG	POS	NEG	NEG

ISOLATE	staphylococcal superantigene-like protein 11				staphylococcal exotoxin-like protein, second locus					Capsule type 1	capsular poly-saccharide synthesis
	**ssl11/set2 (COL)**	**ssl11+set2(Mu50+N315)**	**ssl11+set2(MW2+MSSA47**	**ssl11/set2 (MRSA252)**	**setB3**	**setB3 (MRSA252)**	**setB2**	**setB2 (MRSA252)**	**setB1**	***cap1***	***capH1***
	median(set2-var4)	median(set2-var3)	median(set2-var1)	median(set2-var2)	median(setB-SA11	median(setB-SAR1	median(setB-SA11	median(setB-SAR1	median(setB-SA11	median(hp_capH1_	median(hp_capH1_
**Washington-01_sample-106**	**NEG**	**NEG**	**NEG**	**NEG**	**POS**	**NEG**	**POS**	**NEG**	**POS**	**NEG**	**NEG**
**Washington-02_sample-115**	**NEG**	**NEG**	**NEG**	**NEG**	**POS**	**NEG**	**POS**	**NEG**	**POS**	**NEG**	**NEG**
**Washington-03_sample-117**	**NEG**	**NEG**	**NEG**	**NEG**	**POS**	**NEG**	**POS**	**NEG**	**POS**	**NEG**	**NEG**
**Washington-04_sample-120**	**NEG**	**NEG**	**NEG**	**NEG**	**POS**	**NEG**	**POS**	**NEG**	**POS**	**NEG**	**NEG**
**Washington-05_sample-505**	**NEG**	**NEG**	**NEG**	**NEG**	**POS**	**NEG**	**POS**	**NEG**	**POS**	**NEG**	**NEG**
**Washington-07_sample-611**	**NEG**	**NEG**	**NEG**	**NEG**	**POS**	**NEG**	**POS**	**NEG**	**POS**	**NEG**	**NEG**
**Washington-12_sample-807**	**NEG**	**NEG**	**NEG**	**NEG**	**POS**	**NEG**	**POS**	**NEG**	**POS**	**NEG**	**NEG**
**Washington-13_sample-811**	**NEG**	**NEG**	**NEG**	**NEG**	**POS**	**NEG**	**POS**	**NEG**	**POS**	**NEG**	**NEG**
**Washington-21_sample-2043**	**NEG**	**NEG**	**NEG**	**NEG**	**POS**	**NEG**	**POS**	**NEG**	**POS**	**NEG**	**NEG**
**Washington-17_sample-2006**	**NEG**	**NEG**	**NEG**	**NEG**	**POS**	**NEG**	**POS**	**NEG**	**POS**	**NEG**	**NEG**
**Washington-30_sample-5025_Subclone 1**	**NEG**	**NEG**	**NEG**	**NEG**	**POS**	**NEG**	**POS**	**NEG**	**POS**	**NEG**	**NEG**

From the previous study - for comparison:

**Washington-37_sample-48_ST22_Primate**	**NEG**	**NEG**	**NEG**	**NEG**	**NEG**	**NEG**	**NEG**	**NEG**	**NEG**	**NEG**	**NEG**
**Washington-38_sample-06_ST22_Swine**	**NEG**	**NEG**	**NEG**	**NEG**	**NEG**	**NEG**	**NEG**	**NEG**	**NEG**	**NEG**	**NEG**

ISOLATE	CAPSULE- AND BIOFILM-ASSOCIATED GENES										
	O-antigen poly- merase	capsular poly-saccharide biosyn-	Capsule type 5	capsular poly-saccharide synthesis	O-antigen poly- merase	capsular poly-saccharide biosyn-	Capsule type 8	capsular poly-saccharide synthesis	capsular poly-saccharide biosyn-	O-antigen poly- merase	capsular poly-saccharide biosyn-
	capJ1	***capK1***	***cap 5***	***capH5***	***capJ5***	***capK5***	***cap 8***	***capH8***	***capI8***	***capJ8***	***capK8***
	median(hp_capJ1_(	median(hp_capK1_	median(hp_capH5_	median(hp_capH5_	median(hp_capJ5_(	median(hp_capK5_	median(hp_capH8_	median(hp_capH8_	median(hp_capI8_(	median(hp_capJ8_(	median(hp_capK8_
**Washington-08_sample-640**	**NEG**	**NEG**	**POS**	**POS**	**POS**	**POS**	**NEG**	**NEG**	**NEG**	**NEG**	**NEG**
**Washington-09_sample-657**	**NEG**	**NEG**	**POS**	**POS**	**POS**	**POS**	**NEG**	**NEG**	**NEG**	**NEG**	**NEG**
**Washington-10_sample-801**	**NEG**	**NEG**	**POS**	**POS**	**POS**	**POS**	**NEG**	**NEG**	**NEG**	**NEG**	**NEG**
**Washington-11_sample-804**	**NEG**	**NEG**	**POS**	**POS**	**POS**	**POS**	**NEG**	**NEG**	**NEG**	**NEG**	**NEG**
**Washington-14_sample-1005**	**NEG**	**NEG**	**POS**	**POS**	**POS**	**POS**	**NEG**	**NEG**	**NEG**	**NEG**	**NEG**
**Washington-16_sample-1027**	**NEG**	**NEG**	**POS**	**POS**	**POS**	**POS**	**NEG**	**NEG**	**NEG**	**NEG**	**NEG**
**Washington-18_sample-2021**	**NEG**	**NEG**	**POS**	**POS**	**POS**	**POS**	**NEG**	**NEG**	**NEG**	**NEG**	**NEG**
**Washington-19_sample-2027**	**NEG**	**NEG**	**POS**	**POS**	**POS**	**POS**	**NEG**	**NEG**	**NEG**	**NEG**	**NEG**
**Washington-22_sample-2051**	**NEG**	**NEG**	**POS**	**POS**	**POS**	**POS**	**NEG**	**NEG**	**NEG**	**NEG**	**NEG**
**Washington-23_sample-3009**	**NEG**	**NEG**	**POS**	**POS**	**POS**	**POS**	**NEG**	**NEG**	**NEG**	**NEG**	**NEG**
**Washington-24_sample-3022**	**NEG**	**NEG**	**POS**	**POS**	**POS**	**POS**	**NEG**	**NEG**	**NEG**	**NEG**	**NEG**
**Washington-26_sample-4021**	**NEG**	**NEG**	**POS**	**POS**	**POS**	**POS**	**NEG**	**NEG**	**NEG**	**NEG**	**NEG**
**Washington-27_sample-4033**	**NEG**	**NEG**	**POS**	**POS**	**POS**	**POS**	**NEG**	**NEG**	**NEG**	**NEG**	**NEG**
**Washington-29_sample-5018**	**NEG**	**NEG**	**POS**	**POS**	**POS**	**POS**	**NEG**	**NEG**	**NEG**	**NEG**	**NEG**
**Washington-31_sample-5040**	**NEG**	**NEG**	**POS**	**POS**	**POS**	**POS**	**NEG**	**NEG**	**NEG**	**NEG**	**NEG**
**Washington-30_sample 5025_Subclone 2**	**NEG**	**NEG**	**POS**	**POS**	**POS**	**POS**	**NEG**	**NEG**	**NEG**	**NEG**	**NEG**
**Washington-32_sample-21**	**NEG**	**NEG**	**POS**	**POS**	**POS**	**POS**	**NEG**	**NEG**	**NEG**	**NEG**	**NEG**
**Washington-33_sample-22**	**NEG**	**NEG**	**POS**	**POS**	**POS**	**POS**	**NEG**	**NEG**	**NEG**	**NEG**	**NEG**
**Washington-34_sample-23**	**NEG**	**NEG**	**POS**	**POS**	**POS**	**POS**	**NEG**	**NEG**	**NEG**	**NEG**	**NEG**
**Washington-35_sample-24**	**NEG**	**NEG**	**POS**	**POS**	**POS**	**POS**	**NEG**	**NEG**	**NEG**	**NEG**	**NEG**
**Washington-36_sample-25**	**NEG**	**NEG**	**POS**	**POS**	**POS**	**POS**	**NEG**	**NEG**	**NEG**	**NEG**	**NEG**
**Washington-25_sample-4007**	**NEG**	**NEG**	**POS**	**POS**	**POS**	**POS**	**NEG**	**NEG**	**NEG**	**NEG**	**NEG**
**Washington-28_sample-5008**	**NEG**	**NEG**	**POS**	**POS**	**POS**	**POS**	**NEG**	**NEG**	**NEG**	**NEG**	**NEG**
**Washington-06_sample-556**	**NEG**	**NEG**	**NEG**	**NEG**	**NEG**	**NEG**	**POS**	**POS**	**POS**	**POS**	**POS**
**Washington-15_sample-1007**	**NEG**	**NEG**	**NEG**	**NEG**	**NEG**	**NEG**	**POS**	**POS**	**POS**	**POS**	**POS**
**Washington-20_sample-2039**	**NEG**	**NEG**	**NEG**	**NEG**	**NEG**	**NEG**	**POS**	**POS**	**POS**	**POS**	**POS**

ISOLATE	CAPSULE- AND BIOFILM-ASSOCIATED GENES										
	O-antigen poly- merase	capsular poly-saccharide biosyn-	Capsule type 5	capsular poly-saccharide synthesis	O-antigen poly- merase	capsular poly-saccharide biosyn-	Capsule type 8	capsular poly-saccharide synthesis	capsular poly-saccharide biosyn-	O-antigen poly- merase	capsular poly-saccharide biosyn-
	capJ1	***capK1***	***cap 5***	***capH5***	***capJ5***	***capK5***	***cap 8***	***capH8***	***capI8***	***capJ8***	***capK8***
	median(hp_capJ1_(	median(hp_capK1_	median(hp_capH5_	median(hp_capH5_	median(hp_capJ5_(	median(hp_capK5_	median(hp_capH8_	median(hp_capH8_	median(hp_capI8_(	median(hp_capJ8_(	median(hp_capK8_
**Washington-01_sample-106**	**NEG**	**NEG**	**NEG**	**NEG**	**NEG**	**NEG**	**POS**	**POS**	**POS**	**POS**	**POS**
**Washington-02_sample-115**	**NEG**	**NEG**	**NEG**	**NEG**	**NEG**	**NEG**	**POS**	**POS**	**POS**	**POS**	**POS**
**Washington-03_sample-117**	**NEG**	**NEG**	**NEG**	**NEG**	**NEG**	**NEG**	**POS**	**POS**	**POS**	**POS**	**POS**
**Washington-04_sample-120**	**NEG**	**NEG**	**NEG**	**NEG**	**NEG**	**NEG**	**POS**	**POS**	**POS**	**POS**	**POS**
**Washington-05_sample-505**	**NEG**	**NEG**	**NEG**	**NEG**	**NEG**	**NEG**	**POS**	**POS**	**POS**	**POS**	**POS**
**Washington-07_sample-611**	**NEG**	**NEG**	**NEG**	**NEG**	**NEG**	**NEG**	**POS**	**POS**	**POS**	**POS**	**POS**
**Washington-12_sample-807**	**NEG**	**NEG**	**NEG**	**NEG**	**NEG**	**NEG**	**POS**	**POS**	**POS**	**POS**	**POS**
**Washington-13_sample-811**	**NEG**	**NEG**	**NEG**	**NEG**	**NEG**	**NEG**	**POS**	**POS**	**POS**	**POS**	**POS**
**Washington-21_sample-2043**	**NEG**	**NEG**	**POS**	**POS**	**POS**	**POS**	**NEG**	**NEG**	**NEG**	**NEG**	**NEG**
**Washington-17_sample-2006**	**NEG**	**NEG**	**POS**	**POS**	**POS**	**POS**	**NEG**	**NEG**	**NEG**	**NEG**	**NEG**
**Washington-30_sample-5025_Subclone 1**	**NEG**	**NEG**	**POS**	**POS**	**POS**	**POS**	**NEG**	**NEG**	**NEG**	**NEG**	**NEG**
**From the previous study - for comparison:**											
**Washington-37_sample-48_ST22_Primate**	**NEG**	**NEG**	**POS**	**POS**	**POS**	**POS**	**NEG**	**NEG**	**NEG**	**NEG**	**NEG**
**Washington-38_sample-06_ST22_Swine**	**NEG**	**NEG**	**POS**	**POS**	**POS**	**POS**	**NEG**	**NEG**	**NEG**	**NEG**	**NEG**

ISOLATE					ADHAESION FACTORS / GENES ENCODING MICROBIAL S						
	intercellular adhesion protein A	intercellular adhesion protein C	biofilm PIA synthesis protein D	surface protein involved in	bone sialoprotein-binding protein						
	icaA	***icaC***	***icaD***	***bap***	***bbp***	***bbp* (cons)**	***bbp* (COL+MW2)**	***bbp* (MRSA252)**	***bbp* (Mu50)**	***bbp* (RF122)**	***bbp* (ST45)**
	median(hp_icaA_6	median(hp_icaC_6	median(hp_icaD_6	median(hp_bap_61	WENN((ZÄHLEN	median(hp_bbp_61	WENN((hp_bbp_6	WENN((hp_bbp_6	WENN((hp_bbp_6	WENN((hp_bbp_6	WENN((hp_bbp_6
**Washington-08_sample-640**	**POS**	**POS**	**POS**	**NEG**	**POS**	**POS**	**NEG**	**NEG**	**NEG**	**NEG**	**NEG**
**Washington-09_sample-657**	**POS**	**POS**	**POS**	**NEG**	**POS**	**POS**	**NEG**	**NEG**	**NEG**	**NEG**	**NEG**
**Washington-10_sample-801**	**POS**	**POS**	**POS**	**NEG**	**POS**	**POS**	**NEG**	**NEG**	**NEG**	**NEG**	**NEG**
**Washington-11_sample-804**	**POS**	**POS**	**POS**	**NEG**	**POS**	**POS**	**NEG**	**NEG**	**NEG**	**NEG**	**NEG**
**Washington-14_sample-1005**	**POS**	**POS**	**POS**	**NEG**	**POS**	**POS**	**NEG**	**NEG**	**NEG**	**NEG**	**NEG**
**Washington-16_sample-1027**	**POS**	**POS**	**POS**	**NEG**	**POS**	**POS**	**NEG**	**NEG**	**NEG**	**NEG**	**NEG**
**Washington-18_sample-2021**	**POS**	**POS**	**POS**	**NEG**	**POS**	**POS**	**NEG**	**NEG**	**NEG**	**NEG**	**NEG**
**Washington-19_sample-2027**	**POS**	**POS**	**POS**	**NEG**	**POS**	**POS**	**NEG**	**NEG**	**NEG**	**NEG**	**NEG**
**Washington-22_sample-2051**	**POS**	**POS**	**POS**	**NEG**	**POS**	**POS**	**NEG**	**NEG**	**NEG**	**NEG**	**NEG**
**Washington-23_sample-3009**	**POS**	**POS**	**POS**	**NEG**	**POS**	**POS**	**NEG**	**NEG**	**NEG**	**NEG**	**NEG**
**Washington-24_sample-3022**	**POS**	**POS**	**POS**	**NEG**	**POS**	**POS**	**NEG**	**NEG**	**NEG**	**NEG**	**NEG**
**Washington-26_sample-4021**	**POS**	**POS**	**POS**	**NEG**	**POS**	**POS**	**NEG**	**NEG**	**NEG**	**NEG**	**NEG**
**Washington-27_sample-4033**	**POS**	**POS**	**POS**	**NEG**	**POS**	**POS**	**NEG**	**NEG**	**NEG**	**NEG**	**NEG**
**Washington-29_sample-5018**	**POS**	**POS**	**POS**	**NEG**	**POS**	**POS**	**NEG**	**NEG**	**NEG**	**NEG**	**NEG**
**Washington-31_sample-5040**	**POS**	**POS**	**POS**	**NEG**	**POS**	**POS**	**NEG**	**NEG**	**NEG**	**NEG**	**NEG**
**Washington-30_sample 5025_Subclone 2**	**POS**	**POS**	**POS**	**NEG**	**POS**	**POS**	**NEG**	**NEG**	**POS**	**NEG**	**NEG**
**Washington-32_sample-21**	**POS**	**POS**	**POS**	**NEG**	**POS**	**POS**	**NEG**	**NEG**	**NEG**	**NEG**	**NEG**
**Washington-33_sample-22**	**POS**	**POS**	**POS**	**NEG**	**POS**	**POS**	**NEG**	**NEG**	**NEG**	**NEG**	**NEG**
**Washington-34_sample-23**	**POS**	**POS**	**POS**	**NEG**	**POS**	**POS**	**NEG**	**NEG**	**NEG**	**NEG**	**NEG**
**Washington-35_sample-24**	**POS**	**POS**	**POS**	**NEG**	**POS**	**POS**	**NEG**	**NEG**	**NEG**	**NEG**	**NEG**
**Washington-36_sample-25**	**POS**	**POS**	**POS**	**NEG**	**POS**	**POS**	**NEG**	**NEG**	**NEG**	**NEG**	**NEG**
**Washington-25_sample-4007**	**POS**	**POS**	**POS**	**NEG**	**POS**	**POS**	**NEG**	**NEG**	**NEG**	**NEG**	**NEG**
**Washington-28_sample-5008**	**POS**	**POS**	**POS**	**NEG**	**POS**	**POS**	**NEG**	**NEG**	**NEG**	**NEG**	**NEG**
**Washington-06_sample-556**	**NEG**	**POS**	**POS**	**NEG**	**POS**	**POS**	**NEG**	**NEG**	**POS**	**NEG**	**NEG**
**Washington-15_sample-1007**	**POS**	**POS**	**POS**	**NEG**	**POS**	**POS**	**NEG**	**NEG**	**POS**	**NEG**	**NEG**
**Washington-20_sample-2039**	**POS**	**POS**	**POS**	**NEG**	**POS**	**POS**	**NEG**	**POS**	**NEG**	**NEG**	**NEG**

ISOLATE					ADHAESION FACTORS / GENES ENCODING MICROBIAL SU						
	intercellular adhesion protein A	intercellular adhesion protein C	biofilm PIA synthesis protein D	surface protein involved in	bone sialoprotein-binding protein						
	icaA	*icaC*	*icaD*	*bap*	*bbp*	*bbp* (cons)	*bbp* (COL+MW2)	*bbp* (MRSA252)	*bbp* (Mu50)	*bbp* (RF122)	*bbp* (ST45)
	median(hp_icaA_6	median(hp_icaC_6	median(hp_icaD_6	median(hp_bap_61	WENN((ZÄHLEN	median(hp_bbp_61	WENN((hp_bbp_6	WENN((hp_bbp_6	WENN((hp_bbp_6	WENN((hp_bbp_6	WENN((hp_bbp_6
**Washington-01_sample-106**	POS	POS	POS	NEG	POS	POS	POS	NEG	NEG	NEG	NEG
**Washington-02_sample-115**	POS	POS	POS	NEG	POS	POS	POS	NEG	NEG	NEG	NEG
**Washington-03_sample-117**	POS	POS	POS	NEG	POS	POS	POS	NEG	NEG	NEG	NEG
**Washington-04_sample-120**	POS	POS	POS	NEG	POS	POS	POS	NEG	NEG	NEG	NEG
**Washington-05_sample-505**	POS	POS	POS	NEG	POS	POS	POS	NEG	NEG	NEG	NEG
**Washington-07_sample-611**	POS	POS	POS	NEG	POS	POS	POS	NEG	NEG	NEG	NEG
**Washington-12_sample-807**	POS	POS	POS	NEG	POS	POS	POS	NEG	NEG	NEG	NEG
**Washington-13_sample-811**	POS	POS	POS	NEG	POS	POS	POS	NEG	NEG	NEG	NEG
**Washington-21_sample-2043**	POS	POS	POS	NEG	POS	POS	NEG	NEG	POS	NEG	NEG
**Washington-17_sample-2006**	POS	POS	POS	NEG	POS	POS	NEG	NEG	POS	NEG	NEG
**Washington-30_sample-5025_Subclone 1**	POS	POS	POS	NEG	POS	POS	NEG	NEG	POS	NEG	NEG

**From the previous study - for comparison:**											
**Washington-37_sample-48_ST22_Primate**	POS	AMB	POS	NEG	POS	POS	NEG	NEG	NEG	NEG	NEG
**Washington-38_sample-06_ST22_Swine**	POS	POS	POS	NEG	POS	POS	NEG	NEG	NEG	NEG	NEG

ISOLATE	URFACE COMPONENTS RECOGNIZING ADHESIVE MATRIX MOLECULES (MSCRAMM GENES)									
	clumping factor A					clumping factor B				
	clfA	***clfA* (cons)**	***clfA* (COL+RF122)**	***clfA* (MRSA252)**	***clfA* (Mu50+MW2)**	***clfB***	***clfB* (cons)**	***clfB* (COL+Mu50)**	***clfB* (MW2)**	***clfB* (RF122)**
	WENN((ZÄHLEN	median(hp_clfA_6	WENN((hp_c	WENN((hp_c	WENN((hp_c	WENN((ZÄHLEN	median(hp_clfB_6	WENN((hp_c	WENN((hp_c	WENN((hp_c
**Washington-08_sample-640**	**POS**	**POS**	**NEG**	**NEG**	**POS**	**POS**	**POS**	**NEG**	**NEG**	**NEG**
**Washington-09_sample-657**	**POS**	**POS**	**NEG**	**NEG**	**POS**	**POS**	**POS**	**NEG**	**NEG**	**NEG**
**Washington-10_sample-801**	**POS**	**POS**	**NEG**	**NEG**	**POS**	**POS**	**POS**	**NEG**	**NEG**	**NEG**
**Washington-11_sample-804**	**POS**	**POS**	**NEG**	**NEG**	**POS**	**POS**	**POS**	**NEG**	**NEG**	**NEG**
**Washington-14_sample-1005**	**POS**	**POS**	**NEG**	**NEG**	**POS**	**POS**	**POS**	**NEG**	**NEG**	**NEG**
**Washington-16_sample-1027**	**POS**	**POS**	**NEG**	**NEG**	**POS**	**POS**	**POS**	**NEG**	**NEG**	**NEG**
**Washington-18_sample-2021**	**POS**	**POS**	**NEG**	**NEG**	**POS**	**POS**	**POS**	**NEG**	**NEG**	**NEG**
**Washington-19_sample-2027**	**POS**	**POS**	**NEG**	**NEG**	**POS**	**POS**	**POS**	**NEG**	**NEG**	**NEG**
**Washington-22_sample-2051**	**POS**	**POS**	**NEG**	**NEG**	**POS**	**POS**	**POS**	**NEG**	**NEG**	**NEG**
**Washington-23_sample-3009**	**POS**	**POS**	**NEG**	**NEG**	**POS**	**POS**	**POS**	**NEG**	**NEG**	**NEG**
**Washington-24_sample-3022**	**POS**	**POS**	**NEG**	**NEG**	**POS**	**POS**	**POS**	**NEG**	**NEG**	**NEG**
**Washington-26_sample-4021**	**POS**	**POS**	**NEG**	**NEG**	**POS**	**POS**	**POS**	**NEG**	**NEG**	**NEG**
**Washington-27_sample-4033**	**POS**	**POS**	**NEG**	**NEG**	**POS**	**POS**	**POS**	**NEG**	**NEG**	**NEG**
**Washington-29_sample-5018**	**POS**	**POS**	**NEG**	**NEG**	**POS**	**POS**	**POS**	**NEG**	**NEG**	**NEG**
**Washington-31_sample-5040**	**POS**	**POS**	**NEG**	**NEG**	**POS**	**POS**	**POS**	**NEG**	**NEG**	**NEG**
**Washington-30_sample 5025_Subclone 2**	**POS**	**POS**	**NEG**	**NEG**	**POS**	**POS**	**POS**	**NEG**	**NEG**	**POS**
**Washington-32_sample-21**	**POS**	**POS**	**NEG**	**NEG**	**POS**	**POS**	**POS**	**NEG**	**NEG**	**NEG**
**Washington-33_sample-22**	**POS**	**POS**	**NEG**	**NEG**	**POS**	**POS**	**POS**	**NEG**	**NEG**	**NEG**
**Washington-34_sample-23**	**POS**	**POS**	**NEG**	**NEG**	**POS**	**POS**	**POS**	**NEG**	**NEG**	**NEG**
**Washington-35_sample-24**	**POS**	**POS**	**NEG**	**NEG**	**POS**	**POS**	**POS**	**NEG**	**NEG**	**NEG**
**Washington-36_sample-25**	**POS**	**POS**	**NEG**	**NEG**	**POS**	**POS**	**POS**	**NEG**	**NEG**	**NEG**
**Washington-25_sample-4007**	**POS**	**POS**	**NEG**	**NEG**	**POS**	**POS**	**POS**	**NEG**	**NEG**	**NEG**
**Washington-28_sample-5008**	**POS**	**POS**	**NEG**	**NEG**	**POS**	**POS**	**POS**	**NEG**	**NEG**	**NEG**
**Washington-06_sample-556**	**POS**	**POS**	**NEG**	**NEG**	**POS**	**POS**	**POS**	**NEG**	**NEG**	**NEG**
**Washington-15_sample-1007**	**POS**	**POS**	**NEG**	**NEG**	**POS**	**POS**	**POS**	**NEG**	**NEG**	**NEG**
**Washington-20_sample-2039**	**POS**	**POS**	**NEG**	**NEG**	**POS**	**POS**	**POS**	**NEG**	**NEG**	**NEG**

ISOLATE	URFACE COMPONENTS RECOGNIZING ADHESIVE MATRIX MOLECULES (MSCRAMM GENES)									
	clumping factor A					clumping factor B				
	clfA	*clfA* (cons)	*clfA* (COL+RF122)	*clfA* (MRSA252)	*clfA* (Mu50+MW2)	*clfB*	*clfB* (cons)	*clfB* (COL+Mu50)	*clfB* (MW2)	*clfB* (RF122)
	WENN((ZÄHLEN	median(hp_clfA_6	WENN((hp_c	WENN((hp_c	WENN((hp_c	WENN((ZÄHLEN	median(hp_clfB_6	WENN((hp_c	WENN((hp_c	WENN((hp_c
**Washington-01_sample-106**	**POS**	**POS**	**NEG**	**NEG**	**POS**	**POS**	**POS**	**POS**	**NEG**	**NEG**
**Washington-02_sample-115**	**POS**	**POS**	**NEG**	**NEG**	**POS**	**POS**	**POS**	**POS**	**NEG**	**NEG**
**Washington-03_sample-117**	**POS**	**POS**	**NEG**	**NEG**	**POS**	**POS**	**POS**	**POS**	**NEG**	**NEG**
**Washington-04_sample-120**	**POS**	**POS**	**NEG**	**NEG**	**POS**	**POS**	**POS**	**POS**	**NEG**	**NEG**
**Washington-05_sample-505**	**POS**	**POS**	**NEG**	**NEG**	**POS**	**POS**	**POS**	**POS**	**NEG**	**NEG**
**Washington-07_sample-611**	**POS**	**POS**	**NEG**	**NEG**	**POS**	**POS**	**POS**	**POS**	**NEG**	**NEG**
**Washington-12_sample-807**	**POS**	**POS**	**NEG**	**NEG**	**POS**	**POS**	**POS**	**POS**	**NEG**	**NEG**
**Washington-13_sample-811**	**POS**	**POS**	**NEG**	**NEG**	**POS**	**POS**	**POS**	**POS**	**NEG**	**NEG**
**Washington-21_sample-2043**	**POS**	**POS**	**NEG**	**NEG**	**POS**	**POS**	**POS**	**NEG**	**NEG**	**NEG**
**Washington-17_sample-2006**	**POS**	**POS**	**NEG**	**NEG**	**POS**	**POS**	**POS**	**NEG**	**NEG**	**NEG**
**Washington-30_sample-5025_Subclone 1**	**POS**	**POS**	**NEG**	**NEG**	**POS**	**POS**	**POS**	**NEG**	**NEG**	**POS**
**From the previous study - for comparison:**										
**Washington-37_sample-48_ST22_Primate**	**POS**	**POS**	**NEG**	**NEG**	**POS**	**POS**	**POS**	**NEG**	**NEG**	**NEG**
**Washington-38_sample-06_ST22_Swine**	**POS**	**POS**	**NEG**	**NEG**	**POS**	**POS**	**POS**	**NEG**	**NEG**	**NEG**
